# Glabridin Averts Biofilms Formation in Methicillin-Resistant *Staphylococcus aureus* by Modulation of the Surfaceome

**DOI:** 10.3389/fmicb.2020.01779

**Published:** 2020-09-17

**Authors:** Bhavana Gangwar, Santosh Kumar, Mahendra P. Darokar

**Affiliations:** ^1^Molecular Bioprospection Department, CSIR-Central Institute of Medicinal and Aromatic Plants, Lucknow, India; ^2^Department of Biological Sciences, The University of Texas at Dallas, Richardson, TX, United States

**Keywords:** adhesins, biofilm, cell surface proteome, glabridin, moonlight proteins, MRSA

## Abstract

*Staphylococcus aureus* is an opportunistic bacterium of the human body and a leading cause of nosocomial infections. Methicillin resistant *S. aureus* (MRSA) infections involving biofilm lead to higher mortality and morbidity in patients. Biofilm causes serious clinical issues, as it mitigates entry of antimicrobials to reach the etiological agents. It plays an important role in resilient chronic infections which place an unnecessary burden on antibiotics and the associated costs. To combat drug-resistant infection involving biofilm, there is a need to discover potential anti-biofilm agents. In this study, activity of polyphenolic flavonoid glabridin against biofilm formation of methicillin resistant clinical isolates of *S. aureus* is being reported for the first time. Crystal violet assay and scanning electron microscopy evidences shows that glabridin prevents formation of cells clusters and attachment of methicillin resistant clinical isolate (MRSA 4423) of *S. aureus* to the surface in a dose dependent manner. Gel free proteomic analysis of biofilm matrix by LC-ESI-QTOF confirmed the existence of several proteins known to be involved in cells adhesion. Furthermore, expression analysis of cell surface proteins revealed that glabridin significantly down regulates an abundance of several surface-associated adhesins including fibronectin binding proteins (FnbA, FnbB), serine-aspartate repeat-containing protein D (SdrD), immunoglobulin-binding protein G (Sbi), and other virulence factors which were induced by extracellular glucose in MRSA 4423. In addition, several moonlighting proteins (proteins with multiple functions) such as translation elongation factors (EF-Tu, EF-G), chaperone protein (DnaK), glyceraldehyde 3-phosphate dehydrogenase (GAPDH) and pyruvate kinase (PK) were detected on the cell surface wherein their abundance was inversely proportional to surface-associated adhesins. This study clearly suggests that glabridin prevents biofilm formation in *S. aureus* through modulation of the cell surface proteins.

## Introduction

*Staphylococcus aureus* is a common bacterium of the human body which can cause numerous diseases (Tong et al., 2015). *S. aureus* is considered one of the leading causes of nosocomial and community-acquired infections (Haque et al., 2018). Drug-resistance in *S. aureus* is achieved in several ways, including the acquisition of resistance genes, target alteration, efflux pumps and biofilm (Appelbaum, 2006; Tiwari and Sen, 2006; Foster, 2017; Santajit and Indrawattana, 2016; Gebreyohannes et al., 2019). Like other bacteria, the formation of biofilms in *S. aureus* is an alternative type of microbial growth and a leading cause of resistance to antimicrobials (López et al., 2010; Mah, 2012). Around 80% of chronic and recurrent infections in the human body are associated with bacterial biofilm (Sharma et al., 2019). *S. aureus* cells within biofilms are more resistant to antibiotics than planktonic cells due to the altered environment, multilayered structure, and incomplete penetration of the antibiotics (Mah, 2012; Sharma et al., 2019). Reports suggest that bacteria in the biofilm can be 10–1,000 times more resistant to antimicrobials than their planktonic counterparts (Gebreyohannes et al., 2019). Biofilm growth can prevent entry of the antibiotics during treatment as well as protecting pathogens against host immune defenses. Biofilm formation by MRSA in medical devices, implants, chronic wounds, and host tissue reduces susceptibility to antimicrobial agents (Sharma et al., 2019; Neopane et al., 2018).

The biofilm life cycle of microbes contains four stages, the initial attachment of bacteria, microbial colonies formation, bacterial growth leading to extracellular matrix (ECM) generation and maturation, followed by the dispersal of the bacteria to find new niches (Kostakioti et al., 2013). The ECM components contain extracellular polymeric substances (EPS) including proteins that helps cell to adhere on biotic and abiotic surfaces (Donlan and Costerton, 2002; López et al., 2010; Karygianni et al., 2020). To date, many adhesive proteins have been identified as important components of the attachment process and biofilm matrix development in *S. aureus* (Foster et al., 2014; Jan-Roblero et al., 2016). *S. aureus* cell surface-associated proteins in biofilm are well characterized such as SdrC (BapA homologs), intercellular adhesions (IcaA,D,B,C), fibronectin-binding protein A (FnbA), fibronectin-binding protein B (FnbB), clumping factor B (ClfB), and *S. aureus* surface protein (SasG) (Donlan and Costerton, 2002; Speziale et al., 2014). These surface adhesins are regulated by complex network of transcriptional regulators AlrRS, SarA, Agr, SaeRS and AcrR under various environmental conditions (Paharik and Horswill, 2016). Therefore, study of biofilm formation and finding anti-biofilm agents against pathogens such as *S. aureus* is highly important as it can help choose the right treatment.

Plant derived medicines are gaining interest with regards to controlling antibiotic-resistant bacterial infections and biofilm. Plants are rich in a variety of secondary metabolites such as flavonoids, polyphenol, terpenoids, alkaloids etc. Many plants such as Licorice have been used to cure various bacterial infections in traditional practices by humans since ancient times all over the world (Cowan, 1999; Rohinishree and Negi, 2016; Mamedov and Egamberdieva, 2019). Plant derived phytomolecules have shown antimicrobial and antibiofilm activity against Gram-negative and Gram-positive pathogenic bacteria including *S. aureus* (Sánchez et al., 2016; Rubini et al., 2018). The ethanolic extract of licorice was shown to have antimicrobial and antibiofilm activity against *Porphyromonas gingivalis* (Suwannakul and Chaibenjawong, 2017). *Glycyrrhiza glabra* (Licorice) contains over 20 triterpenoids and nearly 300 flavonoids (Wang et al., 2015). Glabridin (Glb), is a polyphenolic flavonoid compound and one of the most active ingredients present in licorice roots. Glabridin is reported to have antioxidant, anti-inflammatory, anticancer, antiviral, antimicrobial and drug-resistance modifying activity in addition to immunomodulatory, hepatoprotective, and cardioprotective effects (Simmler et al., 2013; Singh et al., 2015). Glabridin has been shown to inhibit Gram-positive and Gram-negative bacteria including biofilm forming pathogens such as *Staphylococcus sp.*, *Pseudomonas aeruginosa*, *Bacillus* sp., *Streptococcus* sp., *Escherichia coli*, *Mycobacterium tuberculosis*, and fungi (Gupta et al., 2013; Gupta et al., 2008; Singh et al., 2015; Irani et al., 2010; Fatima et al., 2009; Wang et al., 2015). Although, glabridin has been reported to possess antimicrobial activity against several pathogens, its detailed mechanism of action is not yet known. In an earlier report, it was found that glabridin in a dose-dependent manner produced reactive oxygen species (ROS) that damages cellular proteins ultimately leading to growth inhibition of *S. aureus* (Singh et al., 2015). This study reports for the first time an antibiofilm activity of glabridin against a glucose-induced biofilm of *S. aureus*. The mechanism of glabridin action has also been determined through the expression of genes/proteins that are involved in biofilm formation in the methicillin resistant clinical isolate MRSA 4423 of *S. aureus*.

## Materials and Methods

### Bacterial Strains, Culture Conditions, Chemicals and Enzymes

The drug-sensitive strain MTCC 96 (ATCC9144) of *S. aureus* was procured from the Microbial Type Culture Collection, CSIR-Institute of Microbial Technology Chandigarh, India. Clinical isolates of *S. aureus* were obtained from Dr. K. N. Prasad (Laboratory of Clinical Microbiology, SGPGIMS, Lucknow, India) (Gupta et al., 2012). Standard Mueller-Hinton agar and cation-adjusted Mueller-Hinton broth media (MHA and MHB, Hi-Media, Mumbai, India) were used to culture bacteria. Tryptic soy broth (TSB, Hi-Media, Mumbai, India) medium was used for Biofilm assays. Glabridin, antibiotics, phosphate-buffered saline, DMSO, glucose and lysostaphin was procured from Sigma-Aldrich (St. Louis, MO, United States). Crystal Violet used in this study was used from Hi-Media (Mumbai, India). Trypsin Gold, Mass Spectrometry Grade was obtained from Promega (Madison, WI, United States). Taq DNA polymerase master mix, c-DNA synthesis kit and SYBR Green qPCR super mix was purchased from Genetix (Thermo, United States).

### Detection of *mecA* Gene and Antimicrobial Susceptibility Testing of *S. aureus* Clinical Isolates

To identify MRSA strains, PCR amplification of the *mecA* gene was performed using the genomic DNA of clinical isolates as template, *mecA* specific primers (Supplementary Table S1) and Taq DNA polymerase master mix as per recommended method in a Mastercycler EP Gradient (Eppendorf, Germany) (Pournajaf et al., 2014). The antibacterial activity of glabridin and antibiotics (belonging to different classes) against *S. aureus* clinical isolates was determined by the broth microdilution assay using 96 ‘U’-bottom micro-titer plates as per CLSI guidelines (CLSI, 2018). *S. aureus* was cultured in 5 mL broth in tubes and incubated overnight at 37 °C in a shaking incubator (Eppendorf, Germany). The initial bacterial inoculum size was standardized to obtain 10^6^ cfu/mL. A series of different concentrations of antibiotics (listed in Supplementary Table S2) was prepared by twofold serial dilution in a 96-well plate, except for the negative control, followed by inoculation with 10^6^ cfu/mL *S. aureus* and 24 h incubation at 37 °C. MIC was determined by finding the lowest concentration which inhibited the growth as per CLSI guidelines (CLSI, 2018).

### Growth Curve Study

To measure the effect of glabridin on the survival of MRSA, three clinical isolates were selected that made strong (MRSA 4423), moderate (MRSA 4627), and weak (MRSA 2071) biofilm along with the sensitive strain MTCC 96. Overnight pre-grown cells were inoculated (10^6^ cfu/ml) in fresh MHB and TSB media and challenged with a range of glabridin (2 MIC to 1/16 MIC). The cultures were grown during shaking (180 rpm) at 37°C for 12 h and optical density was monitored every 1 h time intervals (OD_600__*nm*_) with Multiskan GO Microplate Spectrophotometer (Thermo Scientific, United States). Each test was performed in three culture replicates, averaged and graph were plotted with OD_600__*nm*_ in *Y*-axis and time in *X*-axis.

### Biofilm Formation Assay

The biofilm formation assay was conducted in TSB medium with 1% glucose in three replicates using a 96-well plate (Lade et al., 2019). Suspension of all clinical isolates of MRSA were precultured overnight and ∼10^6^ cfu/mL cell suspension was used to inoculate into each well. For the dose-dependent anti-biofilm assay, glabridin was tested in sub-inhibitory concentrations ranging from 7.8 to 0.78 μg/ml. Control experiments were set with DMSO and without glucose wherever required, TSB medium without cultures was used as a negative control. The plates were incubated at 37 °C for 48 h under static conditions (without shaking). After incubation, the OD was measured at 600 nm then the media was decanted in biohazard collection and wells were carefully rinsed three times with 1X phosphate buffered saline (PBS) to remove non-adherent cells and wash off the media components. The attached biofilm cells in wells were stained with 0.1% crystal violet for 15 min at room temperature (Lade et al., 2019). The excess stain was removed by rinsing with water. The crystal violet bound to attached cells was solubilized with 95% ethanol and quantified by measuring absorbance at OD_595__*nm*_ (Kumar and Spiro, 2017). Absorbance was recorded, and graphs were plotted with values averaged from three biological and three technical replicates in each condition. Effect of glabridin on biofilm formation was calculated by normalizing the absorbance of crystal violet with culture OD (OD_595__*nm*_/OD_600__*nm)*_ of MRSA 4423. The following method described by Stepanović et al. (2000) was used to calculate cut-off OD (OD_*cut*_) value to classify MRSA isolates biofilm formation as weak (+), moderate (++) and strong (+++) based upon the OD_595__*nm.*_

Formula used for biofilm gradation:

OD_*cut*_ = 3 × standard deviation (SD) of ODs above the OD_*avg*_ of negative control.
OD ≤ OD_*cut*_ = Non-biofilm former, OD_*cut*_ < OD ≤ 2 × OD_*cut*_ = Weak biofilm former, 2 × OD_*cut*_ < OD ≤ 4 × OD_*cut*_ = Moderate biofilm former, OD > 4 × OD_*cut*_ = Strong biofilm former.

### Biofilm Dispersal Assay

The biofilm detachment assay was performed as described by Kaplan et al. (2004), Boles and Horswill (2008), and Shukla and Rao (2017). Briefly, after the establishment of MRSA 4423 biofilms in the polystyrene, 96-well microtiter plate, wells were rinsed with 1X PBS. Prior to staining, biofilms were incubated separately with trypsin and proteinase K (1.0 μg/ml each), DNase I and RNase E (10 μg/ml each) for 1 h at 37°C. Microtiter plate wells were washed thrice with deionized water to remove dispersed cells and stained using the 0.1% crystal violet as mentioned above in biofilm assay. The crystal violet dissolved in 95% ethanol was quantified by measuring absorbance at OD_595__*nm*_ using a Multiskan GO Microplate Spectrophotometer (Thermo Scientific, United States). Biofilms with no enzyme treatment were used as a control and medium without bacteria and enzymes was used as a blank. The experiment was set in triplicates and a graph was plotted against averaged values (OD_595__*nm*_) from all sets.

### Scanning Electron Microscopy (SEM) Analysis of MRSA Biofilm

An overnight culture of MRSA 4423 was diluted in TS broth to obtain 10^6^ cfu/ml. Glabridin was tested at 6.25 μg/ml (1/2 MIC), 3.125 μg/ml (1/4 MIC) and 1.56 μg/ml (1/8 MIC). For each concentration, 3 mL of bacterial suspension was supplemented with the desired amount of compound and added into individual wells in a 6-well plate. The plate was incubated at 37 °C for 48 h to allow the formation of biofilm. The bacterial cells were processed for SEM as described elsewhere (Kong et al., 2018). Briefly, the samples were fixed overnight at 4°C in 4% (v/v) glutaraldehyde, rinsed three times with 1X PBS and dehydrated through a graded ethanol series (35, 50, and 70%), followed by 100% propanol. The samples were platinum-coated using a platinum sputtering unit and observed using a scanning electron microscope (JSM-6490, JEOL, Japan).

### ECM-Associated Protein Analysis

The biofilm ECM proteins were extracted using a method as described by Ythier et al. (2012). Briefly, the culture of MRSA 4423 was grown in the presence of glucose for 48 h in static conditions, and non-adherent cells were removed by decanting and rinsing with 1X PBS. To shave off proteins from biofilm ECM, 1 μg/ml trypsin enzyme was used. After 1 h of enzymatic treatment at 37°C, cells were separated by centrifugation at 16,000*g*, 4°C for 15 min, and partially digested proteins in supernatant were transferred into a new tube and the peptide mix was processed for identification by Liquid Chromatography/High Resolution Mass Spectrometry (LC-ESI-QTOF) (Agilent, United States).

### Collection of Differentially Expressed Proteins From the Cell-Surface of *S. aureus*

To identify changes in proteins present on the cell surface of MRSA 4423 due to glucose and glabridin, cultures were grown in presence of 1% glucose and supplemented with glabridin (1/4 MIC). Cells grown only with glucose were used as a biofilm positive control and cells grown without both glucose and glabridin were used as a biofilm negative control. After 48 h of growth under static conditions, 5 ml cells of equal OD were collected from each set grown in triplicates, centrifuged at 4,000*g* for 10 min and supernatant was discarded. Pellets were washed twice with 1X PBS and cells were resuspended in 1 ml of 1X PBS. To collect surface proteins, all cell suspensions were treated with 1 μg/ml trypsin enzyme for 1 h at 37°C (Ythier et al., 2012). Trypsin shaved proteins in supernatants fraction were collected by centrifugation at 16,000*g* for 10 min in 4°C and processed as above for identification by LC-ESI-QTOF (Agilent, United States).

### Identification of Proteins by LC-ESI-QTOF

Trypsin digested proteins were identified by LC-ESI-QTOF as per the standard process described elsewhere (Kaplan et al., 2004; Brun et al., 2020). Briefly, trypsin shaved proteins obtained from cell surface or ECM were treated with DTT and iodoacetamide for 1 h and the samples were digested with trypsin (1 μg/ml) for another 12–16 h at 37°C. The trypsin enzyme was inactivated by adding 5% Tri-Fluro acetic acid (TFA) to the peptide mixtures in tube. Peptide mixtures were pooled from three independent replicates, quantified and purified with C18 matrix column as per recommended protocol (Agilent, United States). Purified peptide mixtures were concentrated using a speed vacuum centrifugation unit at 4°C. Concentrated peptides were resuspended in 1% TFA and 5% acetonitrile (ACN) solution and submitted for identification by LC-ESI-QTOF. The MS/MS data was identified against *S. aureus* protein database (NCBI) using Spectrum Mill software (Agilent, United States) and spectral counts were used to calculate the fold change as described elsewhere (Liu et al., 2004).

### Quantitative Real-Time PCR (qRT-PCR) Analysis

Quantitative real time PCR was performed to check gene expression levels of the differentially expressed proteins found on MRSA 4423 cell-surface under the biofilm condition and in presence of glabridin. Briefly, *S. aureus* cells grown for 48 h were collected by centrifugation at 4°C, 4,000*g* and supernatants were discarded. The cell pellets were washed with 1X PBS twice and stored overnight at −80°C. To extract RNA, pellets were resuspended in a 150 μl lysis buffer containing 50 μg/ml lysostaphin, 10 mM Tris, and 1 mM EDTA in DEPC treated water and incubated for 15 min at 37°C. Total RNA was isolated through the Trizol method and quality was assessed as described previously (Kumar et al., 2012). The DNA free RNA (0.5 μg) was used in reverse transcription and the cDNA was synthesized in a final volume of 20 μl using random hexamer primers as per the manufacturer’s instructions of GeneSure First-Strand cDNA Synthesis Kit for RT-PCR (Thermo, United States). Quantitative RT-PCR (qPCR) was carried out using gene specific primers (Supplementary Table S1) and SYBR Green in a Quantstudio5 real time PCR system (Applied Biosystems, Thermo, United States). The PCR cycling conditions were set according to manufacturer recommendations of the GeneSure^TM^ SYBR Green/ROX Kit (Thermo, United States). Negative controls and genomic DNA contamination controls were included in the experiment. The *S. aureus* 16S rRNA was used as an endogenous control to normalize the expression of each gene. The relative expression (RQ) of target genes of interest were analyzed using ΔΔC_t_ values compared to the untreated control (Atshan et al., 2013; Kong et al., 2018).

### Statistical Analysis

All experiments were performed in three replicates. Data are presented as means ± standard deviation. Statistical analysis was performed using one-way Analysis of Variance (ANOVA) followed by Tukey tests and the difference *p* ≤ 0.05 were considered significant.

## Results

### Assessing Presence of *mecA* Gene and Antimicrobial Susceptibility Profiling of *S. aureus* Clinical Isolates

Clinical isolates of *S. aureus* were tested for the presence of the *mecA* gene known to be a marker for methicillin resistance. All *S. aureus* clinical isolates were verified for the existence of the *mecA* gene in PCR amplification (Supplementary Figure S1). As expected, no amplification of the *mecA* gene was observed in drug sensitive strain MTCC 96 of *S. aureus* (Supplementary Figure S1). All clinical isolates that harbored the *mecA* gene also showed a high level of resistance toward oxacillin and other antibiotics of beta lactam class (Supplementary Table S2). Compared to the sensitive strain MTCC 96, MRSA isolates showed a high level of resistance toward different antibiotics except for daptomycin, bacitracin, tetracycline, linezolid, and teicoplanin to which most of these isolates were found susceptible. In case of glycopeptides, only two clinical isolates (MRSA 2071 and MRSA 1745) showed vancomycin intermediate resistant *S. aureus* (VISA) (Supplementary Table S2).

### Biofilm Formation Ability of MRSA Isolates

**FIGURE 1:**
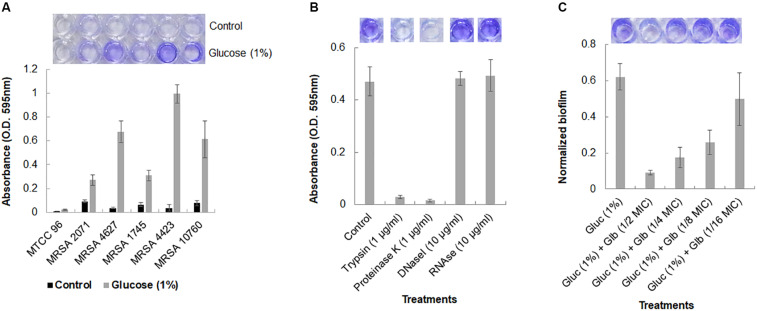
Crystal violet assay for Biofilm formation in MRSA clinical isolates. **(A)** Crystal violet binding assay showing biofilm formation ability of different MRSA isolates to the 96-well polystyrene plate surface (top view) in TSB and TSB supplemented with 1% glucose. *S.* a*ureus* MTCC 96 (first from left) was used as a control. **(B)** Biofilm dispersal assay of MRSA 4423 in 96-well polystyrene surface. Proteases (trypsin and proteinase K) were found to detached pre-formed biofilm on polystyrene surfaces within 1 h of incubation at 37°C (second and third well on the top). Nuclease (DNAse I and RNAse E) were found ineffective to detach cells under the tested conditions. **(C)** An effect of glabridin at different concentration (added in the culture medium) against MRSA 4423. Y-axis showing normalized biofilm absorbance (OD_595nm_/OD_600nm_). More than 50% biofilm reduced biofilm was formed at 1/8 MIC (1.56 μg/ml) of glabridin and 1/2 MIC (6.25 μg/ml) of glabridin treatment almost abolished the attachment of MRSA 4423 cells to a 96-well polystyrene surface. The darkness of the blue color indicates more biofilm formed on the surface. Dye was dissolved in 95% ethanol and quantified by measuring absorbance of crystal violet at 595 nm using spectrophotometer, and the graph was plotted using averaged values (O.D. 595 nm) obtained from three replicates and error bars are showing standard deviations (±SD).

The biofilm formation ability of five MRSA clinical isolates was studied under static conditions. It was observed that MRSA isolates, grown in TSB medium, could not adhere to polystyrene surface. However, the addition of 1% glucose into TSB medium significantly induced the formation of biofilm and cells were found to be adhered on the polystyrene surface as demonstrated by crystal violet staining (Figure 1A). Reference strain MTCC 96 did not form biofilm under either condition. Among all clinical isolates, MRSA 4423 was found to develop a strong biofilm followed by moderate levels of biofilm by MRSA 4627 and MRSA 10760. While the other remaining two MRSA clinical isolates MRSA 2071 and MRSA 1745 were found to form weak biofilm in the presence of 1% glucose (Figure 1A). Therefore, the strong biofilm forming isolate MRSA 4423 was considered for further study.

### Enzymatic Detachment of MRSA Biofilm

The components of the biofilm matrix are usually nucleic acids (eDNA), exopolysaccharide and proteins. It was observed that trypsin and proteinase K treatments were able to remove the attached cells of MRSA 4423 completely from the polystyrene plate (Figure 1B). The selective detachment of biofilm within an hour by low concentration of proteases (trypsin and proteinase K), indicates the involvement of proteins in biofilm extracellular matrix (ECM) attached to polystyrene surfaces.

### Effect of Glabridin on Growth and Biofilm Formation

A strong anti-staphylococcal activity of glabridin was observed with minimal inhibitory concentration (MIC) 12.5 μg/ml against both the drug sensitive strain (MTCC 96) and the MRSA clinical isolate (MRSA 4423) (Supplementary Table S2). To assess the antibiofilm efficacy of glabridin against MRSA 4423 forming strong biofilm, sublethal concentrations of glabridin ranging from 0.78 μg/ml (1/16 MIC) to 6.25 μg/ml (1/2 MIC) were added at the beginning of the experiments in a 96-well plate. Reduced biofilm formation was observed in presence of glabridin at sublethal concentration ranging from 1/4 MIC to 1/16 MIC, while at 1/2 MIC, glabridin was observed to completely prevent the biofilm formation (Figure 1C). To confirm whether reduced biofilm formation in presence of glabridin is independent of its planktonic growth inhibition activity, change in growth under biofilm conditions in the presence and absence of Glabridin was recorded. Thus, a dose dependent effect of Glabridin on biofilm reduction was clearly observed (Figure 1C). Similar observation on biofilm in the presence of glabridin was found against moderate biofilm forming clinical isolate MRSA 4627 (data not shown).

### Growth Curve Study

**FIGURE 2:**
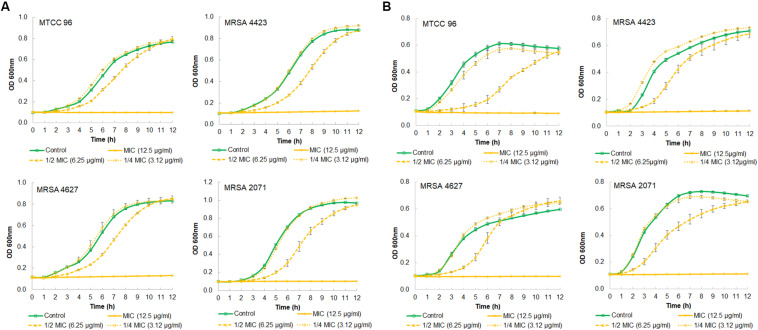
Growth curve showing effect of different concentration of glabridin on viability of *S. aureus.* **(A)** in MHB growth medium of MTCC 96 (top left), MRSA 4423 (MRSA that forms strong biofilm) (top right) MRSA 4627 (MRSA that form moderate biofilm) (lower left) and MRSA 2071 (MRSA that form weak biofilm) (lower right). **(B)** in TSB growth medium of MTCC 96 (top left), MRSA 4423 (MRSA that forms strong biofilm) (top right) MRSA 4627 (lower left) and MRSA 2071 (lower right) in presence of different concentrations of glabridin. Line represents average value obtained from three independent. Graph represents average value from three independent assays and errors bar are showing standard deviation (±SD).

A growth kinetics study, in both TSB and MHB media, showed that glabridin completely inhibited the growth of strong biofilm formation in isolate MRSA 4423 at the concentration of 12.5 μg/ml (MIC value) (Figures 2A,B). Glabridin, at 1/2 MIC concentration, partially inhibited the growth of *S. aureus* which was recovered at around 12 h and reached the optical density close to the control cultures. Similarly, glabridin at 12.5 μg/ml concentration was observed to completely inhibit the growth of other clinical isolates of MRSA (forming moderate and weak biofilm) as well as the drug sensitive strain MTCC 96 (Figures 2A,B). Thus, a dose dependent reduction in growth of MRSA was observed upon exposure to the glabridin and at 12.5 μg/ml (MIC value) concentration.

### Scanning Electron Microscopy (SEM) Analysis of MRSA Biofilm

**FIGURE 3:**
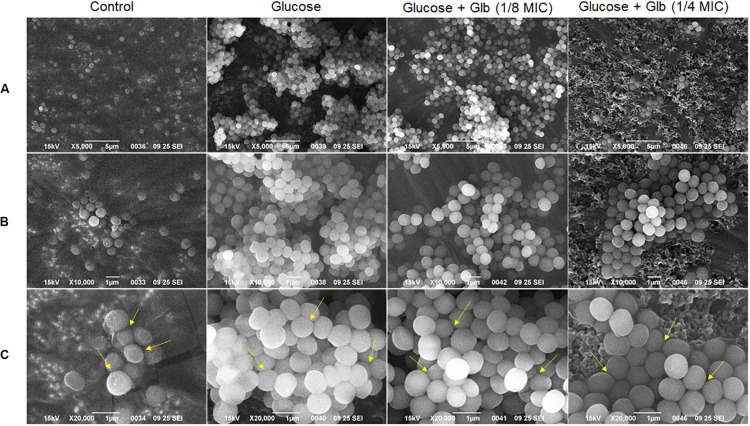
Scanning Electron Microscopy (SEM) micrographs of the MRSA 4423 biofilm. Biofilm formation in control (left), biofilm clusters formed in presence of 1% glucose (second from left), and in presence of glabridin at concentration of 1.56 μg/ml (1/8 MIC) & 3.25 μg/ml (1/4 MIC) (last two row from left to right respectively). **(A)** Top row showing 5,000× magnification. **(B)** Middle row (top to bottom) showing biofilm structures at 10,000×. **(C)** Bottom row showing biofilm cells at 20,000× magnifications. The arrows indicate loosely bound cells or dissociation of cells observed in presence of glabridin compared to clusters formed in only glucose treatment.

Further, the impact of glabridin on biofilm of MRSA 4423 was studied using a scanning electron microscope (SEM) upon the exposure of cells to two different concentrations of glabridin (1/8 and 1/4 MIC). Under the 5,000× magnification, in the control, very few cells were found attached to the surface, but in the presence of 1% glucose, highly organized cell clusters and tunnels were formed between these clusters (Figure 3A). These cell clusters were barely observed when supplemented with glabridin (1/8 MIC), and less tightly bound cells were observed. Furthermore, very less cell clusters compared to biofilm were observed on the surface of cells exposed to 1/4 MIC of glabridin when observed under the 10,000× and 20,000× magnifications (Figures 3B,C).

### Proteomic Analysis of Biofilm Matrix (ECM)

The genome of biofilm forming methicillin-resistant *S. aureus* N315 published at the KEGG database^1^ revealed the presence of surface proteins that are known to play a role in biofilm formation. Since different bacterial strains can express different sets of genes/proteins under similar conditions, therefore, the presence of proteins was checked in the ECM of MRSA 4423 that was formed upon glucose treatment. MS/MS analysis of proteins in ECM and cells imbedded in ECM by LC-ESI-QTOF identified several cell surface proteins involved in adhesions such as mannosyl-glycoprotein endo-beta-*N*-acetylglucosamidase or bifunctional autolysin (Atl), glycerol phosphate lipoteichoic acid synthase of LTA family (ItaS), CHAP domain-containing hypothetical protein (SsaA), immunoglobulin-binding protein (Sbi), *N*-acetylmuramoyl-L-alanine amidase (similar to autolysin), fibronectin-binding proteins (FnbA & FnbB), fibrinogen-binding proteins (Efb) and matrix-binding protein (Ebh). Other proteins including 5′-nucleotidase, gamma-hemolysin subunit B (HlgB), translation elongation factors (EF-Tu & EF-G), glyceraldehyde-3-phosphate dehydrogenase (GAPDH), Peptidase Pbp2a (MecA), and β-lactamase (BlaZ) were also identified in biofilm ECM of MRSA 4423 (Supplementary Table S4). The presence of surface-associated adhesins in ECM correlates well with the protease assay which detached the cells from the polystyrene surface. A lack of cell attachment in the absence of glucose and presence of glabridin suggested these proteins are possibly compromised under these conditions compared to the biofilm forming condition (1% glucose). Therefore, we decided to find differentially expressed proteins on the cell surface due to the presence of glucose and glabridin.

### Analysis of Differentially Expressed Cell Surface Proteins in Presence of Glabridin

Differentially expressed proteins on the cell surface of MRSA 4423 in the biofilm condition were identified with and without the exposure to glabridin. As compared to the control, the supplement of glucose induces expression levels of cell surface-associated proteins such as Atl, FnbA, FnbB, SdrD, 5′-nucleotidase, Sbi, SsaA, *N*-acetylmuramoyl-L-alanine amidase, hypothetical protein with LPXTG-domain (similar to 5′-nucleotidase), and FKLRK-domain that are several-fold higher. Exposure of cells to glabridin downregulated the abundance of many glucose-induced proteins such as FnbAB, SdrD, Sbi, FKLRK-protein, LPXTG-protein, Efb, 5′-nucleotidase and *N*-acetylmuramoyl-L-alanine amidase. However, abundance of other cell surface proteins such as Atl and CHAP-domain proteins did not change significantly.

**FIGURE 4:**
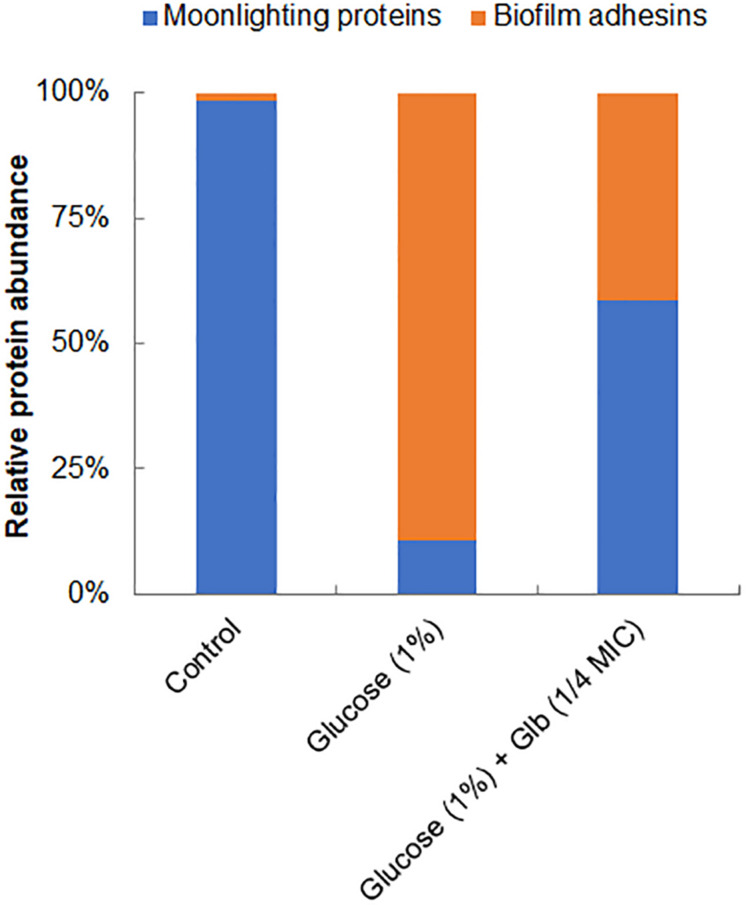
Relative abundance of major proteins identified on cell surface. Graph showing relative ratio of selected biofilm adhesins (FnbAB, Sbi, SdrD, Atl and FKLRK) to moonlight proteins (EF-T, EF-G, DnaK, GAPDH and PK) identified on cell surface of MRSA 4423 under different conditions (Table 1). Percentage distribution was calculated using a fraction of an individual protein’s spectral counts (Ni) divided by total spectral counts (Na) observed in all conditions using formula [(Ni/Na) × 100].

Interestingly, on the cell surface of MRSA 4423, several moonlight proteins (a single protein with multiple functions) such as EF-Tu, EF-G, GAPDH, molecular chaperone (DnaK) and pyruvate kinase (PK) were also identified (Table 1). The abundance of these moonlight proteins was reduced on the surface of cells supplemented with glucose (Table 1). However, treatment of glabridin reversed the effect of glucose. Thus, the abundance of moonlighting proteins was found correlated oppositely to the surface-associated adhesins on the cell surface (Figure 4).

**TABLE 1:**
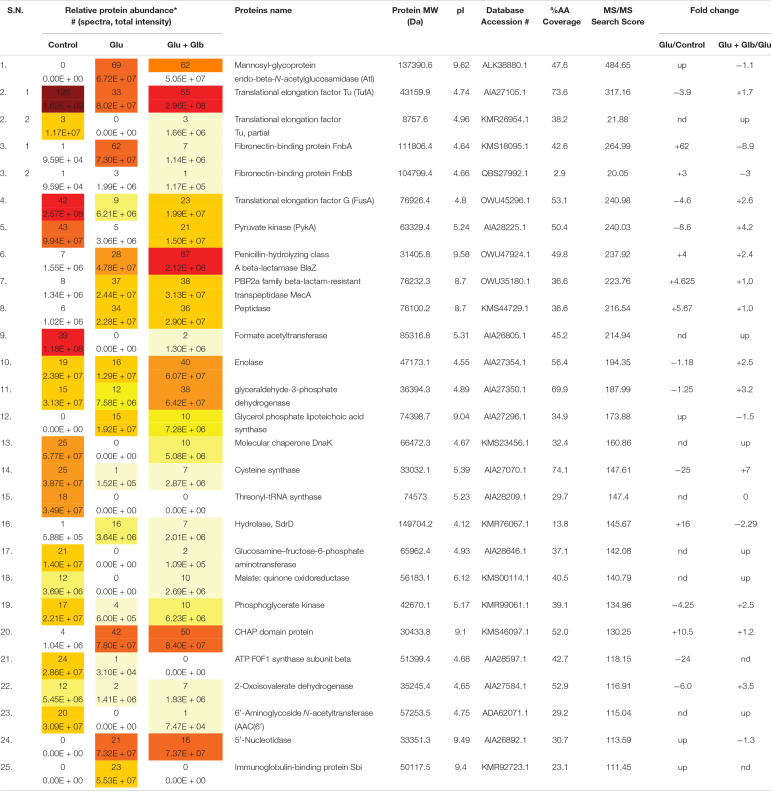
Protein list showing the top 25 hits identified by LC-ESI-QTOF analysis of MRSA 4423 cell surface proteome upon the exposure of glabridin.

*Glu = Glucose, Glb = Glabridin. Glu/Control = shows fold change due to presence of glucose compared to control, and Glu + Glb/Glu = shows fold change due glabridin treatment compared to glucose treatment (biofilm inducing condition). Color intensity shows relative abundance of protein under control and treatments conditions as observed in analysis by Spectrum mill software (Agilent, United States). The darkness of the color (from light yellow to dark red) represents a higher abundance of proteins in the samples. Full list of protein can be accessed in Supplementary Table S5. up = denotes uniquely present proteins, nd = denotes not detected/absence of protein. A probability score of P < 0.05 was used as the criterion for identification. Total protein was pooled from three replicates in each case, quantified, and an equal amount of protein was processed from each set for identification.

### Gene Expression Analysis

**FIGURE 5:**
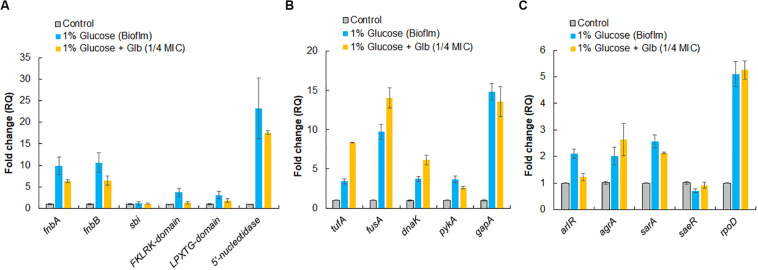
Relative expression analysis of selected genes by qRT-PCR. **(A)** Genes encoding surface-associated adhesins. **(B)** Genes encoding moonlight proteins identified on cell surface of MRSA 4423. **(C)** Master regulator that controls genes involved in adhesion and housekeeping sigma factor, *rpoD*, of *S. aureus.* The RQ values in *Y*-axis are showing fold-changes in genes expression (mRNA) relative to the untreated control. All values shown here are averages (±standard deviation) from three independent samples.

To validate expression of proteins identified by LC-ESI-QTOF on the cell surface, qRT-PCR was performed for both categories of genes encoding surface-associated adhesins and moonlight proteins under the similar growth conditions used for surface protein identification. In comparison to the control, the culture grown under the biofilm inducing condition showed higher expression levels of genes for surface adhesins *fnbA* (9.9-fold), *fnbB* (10.6-fold), *5′-nucleotide* (23-fold), *FKLRK-*domain (3.7-fold) and *LPXTG*-domain (3.1-fold) except gene *sbi* which remained unaffected (Figure 5A). However, glabridin (at 1/4 MIC) was found to significantly down regulate the expression of genes for these surface adhesins *fnbA* (−1.6-fold), *fnbB* (−1.6-fold), *5*′*-nucleotidasee* (−1.3-fold), *FKLRK-*domain (−2.8-fold) and *LPXTG*-domain (−1.7-fold) when compared to the biofilm inducing condition (Figure 5A).

Interestingly, up regulation in expression of genes for moonlight proteins such as *tufA* (3.4-fold), *fusA* (9.7-fold), *dnaK* (3.7-fold), *pykA* (3.7-fold) and *gapA* (14.8-fold) was also observed under the biofilm inducing conditions compared to the control. In the presence of glabridin, further enhancement in expression of *tufA* (2.5-fold), *fusA* (∼1.5-fold) and *dnaK* (1.7-fold) was detected (Figure 5B). It was also observed that genes master regulators of biofilm such as *arlR*, *sarA*, and *agrA* were upregulated more than 2 fold under the biofilm inducing conditions compared to the control and glabridin downregulated expression of *arlR* (−1.7-fold) and *sarA* to some extent (−1.2-fold), while expression of *agrA* was marginally upregulated (1.3-fold) compared to the biofilm inducing conditions. Expression of *saeR* was decreased (−1.4-fold) under the biofilm inducing condition compared to the control and glabridin was able to restore its expression to the level of control. Housekeeping sigma factor, *rpoD*, expression was found to increase about 5-fold under the biofilm inducing condition than control and its expression didn’t change significantly in the presence of glabridin (Figure 5C).

## Discussion

Microorganisms including several pathogenic bacteria produce biofilms as a survival strategy under harsh environments and biofilm embedded bacteria are often a challenge to treat using drugs. Various reports correlate drug resistance with the biofilm formation ability of *S. aureus* and other microorganisms (Awoke et al., 2019; Reffuveille et al., 2017; Sharma et al., 2019). To date, several environmental factors such as glucose, salt (NaCl), ethanol, high temperature, antibiotics stress, and low oxygen have been described to support biofilm formation in *S. aureus* (Jefferson, 2004; Fitzpatrick et al., 2005). Recent studies have focused on the development of antimicrobial drugs to inhibit the formation of biofilm and/or other virulence factors which appear promising with regards to controlling bacterial infections without spreading resistance (Lee et al., 2013, 2014, 2016; Phuong et al., 2017; Kong et al., 2018; Selvaraj et al., 2019).

In crystal violet binding assay, clinical isolate MRSA 4423 was found forming robust biofilms when supplemented with 1% glucose in TSB culture media, which is in accordance with earlier reports (Croes et al., 2009; Lim et al., 2004). Biofilm formation in TSB culture media, supplemented with 1% glucose has been shown to promote the formation of biofilms in *S. aureus* with greater reproducibility (Lade et al., 2019). Beside bactericidal activity, Glabridin was found to prevent the development of glucose-induced biofilm in MRSA 4423 in a concentration dependent manner. The growth curve study suggests that glabridin is capable of inhibiting planktonic growth of MRSA isolates independent of its ability to inhibit biofilm formation, most likely due to its adverse impact on biofilm formation and lack of resistance mechanism toward glabridin in clinical isolates of MRSA. Furthermore, SEM analysis showed that glabridin can effectively inhibit the formation of cell-clusters leading to a biofilm condition.

Exogenously added proteases such as proteinase K and trypsin have often been used as effective biofilm dispersal agents, likely by surface structure degradation (Boles and Horswill, 2008; Chaignon et al., 2007). Proteinase-K cleaves the peptide bonds of aliphatic, aromatic, or hydrophobic amino acids, while trypsin specifically cleaves peptide bonds of lysine and arginine (Chaignon et al., 2007). Identification of highly abundant proteins in the biofilm ECM indicates why the proteases, not nucleases, were capable of detaching the biofilm of MRSA 4423 from the surface presumably because either the DNA contributed insignificantly or was mounted by proteins. Comparative analysis revealed various highly abundant surface-associated adhesins whose expression was enhanced in the presence of glucose as compared to the non-biofilm condition (culture grown without glucose). Upon the exposure of cells to glabridin, down-regulation in the expression of surface proteins like FnbA, FnbB, SdrD, 5′-nucleotidase, Sbi, a hypothetical protein with LPXTG-domain, FKLRK-domain protein, Hld and Efb homolog was observed. Expression of some other cell surface proteins such as Atl, peptidase and CHAP-domain did not change significantly, suggesting that under the tested conditions these are probably not regulated by the response elevated by glabridin in MRSA 4423 (Table 1). Interestingly, most of the proteins abundant in biofilm ECM were basic in nature and on averaged isoelectric point (PI) of most abundant proteins presents in top of the list showed PI > 7 (Supplementary Table S4). However, most of the proteins abundant on the cell surface were acidic in nature with an average PI < 7 (Supplementary Table S5). Since the bacterial cell surface is negative in nature, therefore it possibly favors acidic proteins to attach to. Importantly, down regulation of adhesion proteins in the presence of glabridin correlated well with biofilm formation preventing activity as observed in crystal violet binding assay (Figure 1C). The role of several surface-associated adhesins identified on the cell surface of *S. aureus* have been characterized in detail (Foster et al., 2014; Kwiecinski et al., 2014). The function of some of the most abundant surface-associated proteins identified in this study are described in Supplementary Table S3.

Expression analysis of genes encoding surface proteins by qRT-PCR showed upregulation of adhesins which is in accordance with other studies where surface-associated genes are upregulated in biofilm of *S. aureus* (Speziale et al., 2014; Hiltunen et al., 2019). Glabridin was found to significantly downregulate the expression of surface-associated biofilm adhesins such as *fnbA*, *fnbB*, *sbi* etc. in qRT-PCR analysis which might be an appropriate reason for low abundance of these adhesins on the cell surface in response to glabridin in MRSA 4423. Transcriptional regulatory network of surface-associated adhesins is very complex where multiple gene regulators such as *agrA*, *sarA*, *saeR/S*, and *arlRS* inter-play to control expression of these proteins and fine-tune the formation of biofilm (Paharik and Horswill, 2016; Burgui et al., 2018; Toledo-Arana et al., 2005). Therefore, expression of transcriptional regulators was further confirmed at transcript level.

Indeed, qRT-PCR analysis showed change in expression level of key regulators that are involved in biofilm formation. The expression of *arlR* was found upregulated in the presence of glucose (biofilm inducing conditions) as compared to the control, while in the presence of glabridin, significant down regulation was observed suggesting that *arlR* plays positive role in biofilm formation of MRSA 4423. Activation of quorum sensing regulator, *agrA*, is known to trigger production of exo-proteases responsible for detachment of established biofilms in *S. aureus* (Boles and Horswill, 2008; Yarwood et al., 2004; Vuong et al., 2000). Thus, upregulation of *agrA* in presence of glabridin under biofilm conditions might be able to induce a response which is inhibiting biofilm formation in MRSA 4423.

Transcription regulator *sarA* is known to positively regulate biofilm proteins (FnBPs, PIA, BapA) and inhibits *agrA* activity in *S. aureus* (Wolz et al., 2000; Paharik and Horswill, 2016). Transcriptional regulator *sarA* expression in presence of glucose (biofilm inducing condition) was found to be upregulated, while it was down regulated in presence of glabridin, which is positively correlated to lower level of surface protein adhesins (FnbAB) upon the exposure to glabridin in MRSA 4423. The *saeR* controls both of the factors that are known to promote the biofilm formation and dispersal, and therefore affects the biofilm formation either positively or negatively depending on growth conditions and strain backgrounds of *S. aureus* (Harraghy et al., 2005; Liu et al., 2016). There was no significant change observed in the expression level of *saeR* under the tested conditions. High expression of *rpoD* was found under the biofilm conditions in MRSA 4423 suggesting its requirement for the expression of housekeeping genes and metabolic factors to survive and persist in biofilm which is in accordance with other studies where *rpoD* expression was shown to increase during biofilm formation in *Helicobacter pylori*, and *Xylella fastidiosa* (De la Cruz et al., 2017; de Souza et al., 2004).

**FIGURE 6:**
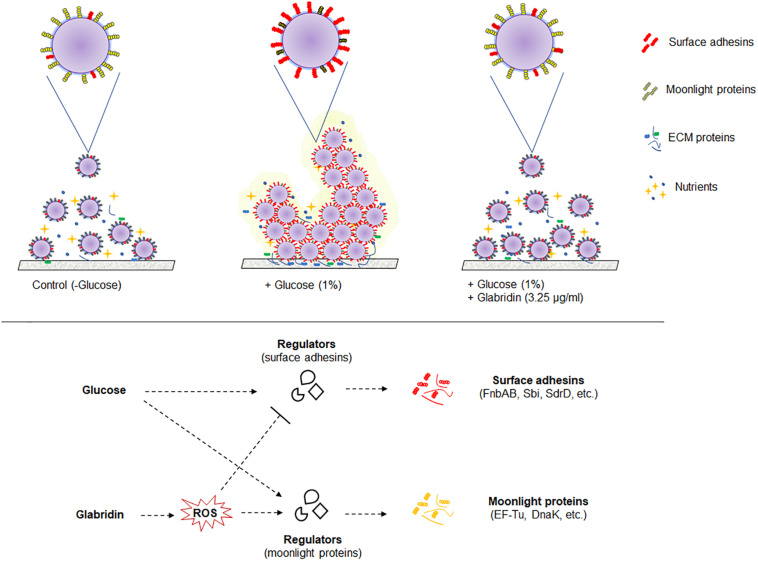
A model of the biofilm’s cell surface and its modulation by glabridin in MRSA 4423. Based on this work model showing regulation of cell surface proteins in biofilm and its modulation by glabridin which induce reactive oxygen species (ROS) in MRSA (Singh et al., 2015).

The study of cell surface proteins of MRSA 4423 led to identification of several other highly abundant proteins such as EF-Tu, EF-G, DnaK, PK and GAPDH etc. which are commonly known as moonlighting proteins. On the cell surface of MRSA 4423, the abundance of many moonlight proteins was found inversely proportional to the biofilm related proteins in the presence of two different treatments, glucose and glabridin. Analysis of their gene expression in presence of glucose showed higher expression level. Similarly, other biofilm studies in bacteria have also showed higher expression of several cellular proteins that play moonlighting functions including EF-Tu, EF-G, and DnaK (Resch et al., 2005; Zimaro et al., 2013). Higher transcript level (mRNA) in the presence of glucose indicating cells are metabolically more active in biofilm conditions than in the control (planktonic culture). Interestingly, glabridin further increased expression of genes encoding EF-Tu, EF-G and DnaK proteins, probably because glabridin triggers ROS generation, leading to a damage response that enhances expression of these protein with chaperones properties (Singh et al., 2015; Caldas et al., 1998; Caldas et al., 2000; Singh et al., 2007). Thus, reduced expression of biofilm-adhesins in presence of glabridin possibly favors the attachment of moonlight proteins to the cell surface leading to alteration in the surfaceome architecture that ultimately influences adherence of MRSA 4423 (Figure 6).

Moonlighting proteins are reported to play important role, as a second function, in interaction with other proteins (Supplementary Table S4). To date, around four hundred moonlight proteins are listed in MoonProt database^2^ which are hypothesized to contribute in bacterial virulence, adhesion and modulation of cell signaling processes (Wang et al., 2014). The moonlighting protein EF-Tu is recognized as pathogen-associated molecular pattern (PAMP) by the pattern recognition receptors (PRRs) present on the surface of host cells (Harvey et al., 2019; Furukawa et al., 2014). Thus, in biofilm conditions, downregulation of moonlighting proteins and upregulation of surface-associated adhesins could be a survival strategy of *S. aureus* to colonize within a host without eliciting an immune response (Zipfel, 2008). The mechanism identified in this study by which glabridin prevents biofilm formation could lead to the development of novel agents against *S. aureus* and other pathogenic bacteria. Reported literature suggests that glabridin is non-cytotoxic and a suitable candidate for drug development as it has been found safe when administered orally (Aoki et al., 2007; Cheema et al., 2014). Further studies may help to understand the association between moonlight and biofilm-related proteins, their role in virulence and to tackle drug resistance in bacteria.

## Conclusion

In the present study, it has been experimentally demonstrated that glabridin, a polyphenolic flavonoid, as a possible agent preventing biofilm formation in methicillin-resistant clinical isolate of *S. aureus*. Proteomics and real-time qPCR expression analysis upon the exposure to glabridin resulted in alteration of surface proteins that are known to be involved in biofilm formation of *S. aureus*. Besides adhesins, proteins known as moonlighting proteins whose expression is inversely proportional to biofilm-associated adhesins were found to be affected in the presence of glabridin. To the best of our knowledge, this is the first experimental evidence demonstrating involvement of glabridin in controlling expression of cell surface proteins and preventing adherence of *S. aureus*. Therefore, glabridin is likely a good candidate to be developed as phytopharmaceutical agent against *S. aureus* preventing biofilm formation.

## Data Availability Statement

All datasets presented in this study are included in the article/Supplementary Material.

## Author Contributions

BG and SK performed the experiments and analyzed the data. BG, SK, and MD conceived the idea and wrote the manuscript. All authors read and approved the manuscript.

## Conflict of Interest

The authors declare that the research was conducted in the absence of any commercial or financial relationships that could be construed as a potential conflict of interest.

## Footnotes

1 https://www.genome.jp/dbget-bin/www_bget?gn:T00051

2 http://www.moonlightingproteins.org/

## References

[B1] Aoki F. Nakagawa K. Kitano M. (2007). Clinical safety of licorice flavonoid oil (LFO) and pharmacokinetics of glabridin in healthy humans. *J. Am. Coll. Nutr.* 26 209-218. 10.1080/07315724.2007.10719603 17634165

[B2] Appelbaum P. C. (2006). The emergence of vancomycin-intermediate and vancomycin-resistant *Staphylococcus aureus*. *Clin. Microbiol. Infect.* 12 16–23. 10.1111/j.1469-0691.2006.01344.x 16445720

[B3] Atshan S. S. Shamsudin M. N. Karunanidhi A. van Belkum A. Lung L. T. Sekawi Z. (2013). Quantitative PCR analysis of genes expressed during biofilm development of methicillin resistant *Staphylococcus aureus* (MRSA). *Infect. Genet. Evol.* 18 106–112. 10.1016/j.meegid.2013.05.002 23669446

[B4] Awoke N. Kassa T. Teshager L. (2019). Magnitude of biofilm formation and antimicrobial resistance pattern of bacteria isolated from urinary catheterized inpatients of jimma university medical center, Southwest Ethiopia. *Int. J. Microbiol.* 2019:5729568. 10.1155/2019/5729568 30881456 PMC6387724

[B5] Boles B. R. Horswill A. R. (2008). Agr-mediated dispersal of *Staphylococcus aureus* biofilms. *PLoS Pathog.* 4:e1000052. 10.1371/journal.ppat.1000052 18437240 PMC2329812

[B6] Brun A. Magallanes M. E. Martínez del Rio C. Barrett-Wilt G. A. Karasov W. H. Caviedes-Vidal E. (2020). A Fast and Accurate Method to Identify and Quantify Enzymes in Brush-Border Membranes: In Situ Hydrolysis Followed by Nano LC-MS/MS. *Methods Protoc.* 3:15. 10.3390/mps3010015 32050538 PMC7189658

[B7] Burgui S. Gil C. Solano C. Lasa I. Valle J. (2018). A systematic evaluation of the two-component systems network reveals that ArlRS is a key regulator of catheter colonization by *Staphylococcus aureus*. *Front. Microbiol.* 9:342. 10.3389/fmicb.2018.00342 29563900 PMC5845881

[B8] Caldas T. Laalami S. Richarme G. (2000). Chaperone properties of bacterial elongation factor EF-G and initiation factor IF2. *J. Biol. Chem.* 275 855–860. 10.1074/jbc.275.2.855 10625618

[B9] Caldas T. D. El Yaagoubi A. Richarme G. (1998). Chaperone properties of bacterial elongation factor EF-Tu. *J. Biol. Chem.* 273 11478–11482. 10.1074/jbc.273.19.11478 9565560

[B10] Chaignon P. Sadovskaya I. Ragunah Ch Ramasubbu N. Kaplan J. B. Jabbouri S. (2007). Susceptibility of staphylococcal biofilms to enzymatic treatments depends on their chemical composition. *Appl. Microbiol. Biotechnol.* 75:125. 10.1007/s00253-006-0790-y 17221196

[B11] Cheema H. S. Prakash O. Pal A. Khan F. Bawankule D. U. Darokar M. P. (2014). Glabridin induces oxidative stress mediated apoptosis like cell death of malaria parasite *Plasmodium falciparum*. *Parasitol. Int.* 63 349-358. 10.1016/j.parint.2013.12.005 24361284

[B12] CLSI (2018). *Methods for Dilution Antimicrobial Susceptibility Tests for Bacteria That Grow Aerobically. 11th ed. CLSI supplement M07.* Wayne, PA: Clinical and Laboratory Standards Institute.

[B13] Cowan M. M. (1999). Plant products as antimicrobial agents. *Clin. Microbiol. Rev.* 12 564–582. 10515903 10.1128/cmr.12.4.564 PMC88925

[B14] Croes S. Deurenberg R. H. Boumans M. L. Beisser P. S. Neef C. Stobberingh E. E. (2009). *Staphylococcus aureus* biofilm formation at the physiologic glucose concentration depends on the *S. aureus* lineage. *BMC Microbiol.* 9:229. 10.1186/1471-2180-9-229 19863820 PMC2774858

[B15] De la Cruz M. A. Ares M. A. von Bargen K. Panunzi L. G. Martínez-Cruz J. Valdez-Salazar H. A. (2017). Gene expression profiling of transcription factors of *Helicobacter pylori* under different environmental conditions. *Front. Microbiol.* 8:615. 10.3389/fmicb.2017.00615 28443084 PMC5385360

[B16] de Souza A. A. Takita M. A. Coletta-Filho H. D. Caldana C. Yanai G. M. Muto N. H. (2004). Gene expression profile of the plant pathogen *Xylella fastidiosa* during biofilm formation in vitro. *FEMS Microbiol. Lett.* 237 341–353. 10.1016/j.femsle.2004.06.055 15321682

[B17] Donlan R. M. Costerton J. W. (2002). Biofilms: survival mechanisms of clinically relevant microorganisms. *Clin. Microbiol. Rev.* 15 167–193. 10.1128/cmr.15.2.167-193.2002 11932229 PMC118068

[B18] Fatima A. Gupta V. K. Luqman S. Negi A. S. Kumar J. K. Shanker K. (2009). Antifungal activity of *Glycyrrhiza glabra* extracts and its active constituent glabridin. *Phytother. Res.* 23 1190–1193. 10.1002/ptr.2726 19170157

[B19] Fitzpatrick F. Humphreys H. O’Gara J. P. (2005). The genetics of staphylococcal biofilm formation–will a greater understanding of pathogenesis lead to better man-agement of device-related infection. *Clin. Microbiol. Infect.* 11 967–973. 10.1111/j.1469-0691.2005.01274.x 16307550

[B20] Foster T. J. (2017). Antibiotic resistance in *Staphylococcus aureus*. Current status and future prospects. *FEMS Microbiol. Res.* 41 430–449. 10.1093/femsre/fux007 28419231

[B21] Foster T. J. Geoghegan J. A. Ganesh V. K. Höök M. (2014). Adhesion, invasion and evasion: the many functions of the surface proteins of *Staphylococcus aureus*. *Nat. Rev. Microbiol.* 12 49–62. 10.1038/nrmicro3161 24336184 PMC5708296

[B22] Furukawa T. Inagaki H. Takai R. Hirai H. Che F. S. (2014). Two distinct EF-Tu epitopes induce immune responses in rice and Arabidopsis. *Mol. Plant Microbe Interact.* 27 113–124. 10.1094/MPMI-10-13-0304-R 24200076

[B23] Gebreyohannes G. Nyerere A. Bii C. Sbhatu D. B. (2019). Challenges of intervention, treatment, and antibiotic resistance of biofilm-forming microorganisms. *Heliyon* 5:e02192. 10.1016/j.heliyon.2019.e02192 31463386 PMC6709409

[B24] Gupta A. Maheshwari D. K. Khandelwal G. (2013). Antibacterial activity of *Glycyrrhiza glabra* roots against certain gram-positive and gram-negative bacterial strains. *J. Appl. Nat. Sci.* 5 459–464. 10.31018/jans.v5i2.354

[B25] Gupta V. K. Fatima A. Faridi U. Negi A. S. Shanker K. Kumar J. K. (2008). Antimicrobial potential of *Glycyrrhiza glabra* roots. *J. Ethnopharmacol.* 5 377–380. 10.1016/j.jep.2007.11.037 18182260

[B26] Gupta V. K. Verma S. Gupta S. Singh A. Pal A. Srivastava S. K. (2012). Membrane-damaging potential of natural L-(−)-usnic acid in *Staphylococcus aureus*. *Eur. J. Clin. Microb. Infect. Dis.* 31 3375–3383. 10.1007/s10096-012-1706-7 22865029

[B27] Haque M. Sartelli M. McKimm J. Abu Bakar M. (2018). Health care-associated infections - an overview. *Infect. and Drug Resist.* 11 2321–2333. 10.2147/IDR.S177247 30532565 PMC6245375

[B28] Harraghy N. Kormanec J. Wolz C. Homerova D. Goerke C. Ohlsen K. (2005). sae is essential for expression of the staphylococcal adhesins Eap and Emp. *Microbiology* 151 1789–1800. 10.1099/mic.0.27902-0 15941988

[B29] Harvey K. L. Jarocki V. M. Charles I. G. Djordjevic S. P. (2019). The diverse functional roles of elongation factor Tu (EF-Tu) in microbial pathogenesis. *Front. Microbiol.* 10:2351. 10.3389/fmicb.2019.02351 31708880 PMC6822514

[B30] Hiltunen A. K. Savijoki K. Nyman T. A. Miettinen I. Ihalainen P. Peltonen J. (2019). Structural and functional dynamics of *Staphylococcus aureus* biofilms and biofilm matrix proteins on different clinical materials. *Microorganisms* 7:584. 10.3390/microorganisms7120584 31756969 PMC6955704

[B31] Irani M. Sarmadi M. Bernard F. Ebrahimi Pour G. H. Shaker Bazarnov H. (2010). Leaves antimicrobial activity of *Glycyrrhiza glabra* L. *Iran. J. Pharm. Res.* 9 425–428. 24381608 PMC3870067

[B32] Jan-Roblero J. Rodriguez-Martinez S. Cancino-Diaz M. E. Candino-Diaz J. C. (2016). “Staphylococcus biofilms,” in *Microbial Biofilms-Importance and Applications*, eds Dhanasekaran D. Thajuddin N. (Rijeka: InTech), 10.5772/62943

[B33] Jefferson K. K. (2004). What drives bacteria to produce a biofilm? *FEMS Microbiol. Lett.* 236 163–173. 10.1016/j.femsle.2004.06.005 15251193

[B34] Kaplan J. B. Velliyagounder K. Ragunath C. Rohde H. Mack D. Knobloch J. K. (2004). Genes involved in the synthesis and degradation of matrix polysaccharide in Actino-bacillus actinomycetemcomitans and *Actinobacillus pleuropneu*-moniae biofilms. *J. Bacteriol.* 186 8213–8220. 10.1128/JB.186.24.8213-8220.2004 15576769 PMC532409

[B35] Karygianni L. Ren Z. Koo H. Thurnheer T. (2020). Biofilm matrixome: extracellular components in structured microbial communities. *Trends Microbiol.* 28 668–681. 10.1016/j.tim.2020.03.016 32663461

[B36] Kong C. Chee C. F. Richter K. Thomas N. Abd Rahman N. Nathan S. (2018). Suppression of *Staphylococcus aureus* biofilm formation and virulence by a benzimidazole derivative. UM-C162. *Sci. Rep.* 8:2758. 10.1038/s41598-018-21141-2 29426873 PMC5807447

[B37] Kostakioti M. Hadjifrangiskou M. Hultgren S. J. (2013). Bacterial biofilms: development, dispersal, and therapeutic strategies in the dawn of the post antibiotic era. *Cold Spring Harb. Perspect. Med.* 3:a010306. 10.1101/cshperspect.a010306 23545571 PMC3683961

[B38] Kumar S. Rai A. K. Mishra M. N. Shukla M. Singh P. K. Tripathi A. K. (2012). RpoH2 sigma factor controls the photooxidative stress response in a non-photosynthetic rhizobacterium. *Azospirillum brasilense* Sp7. *Microbiology* 158 2891–2902. 10.1099/mic.0.062380-0 23023973

[B39] Kumar S. Spiro S. (2017). Environmental and genetic determinants of biofilm formation in *Paracoccus denitrificans*. *mSphere* 2 e00350-17. 10.1128/mSphereDirect.00350-17 28904996 PMC5588039

[B40] Kwiecinski J. Jin T. Josefsson E. (2014). Surface proteins of *Staphylococcus aureus* play an important role in experimental skin infection. *APMIS* 122 1240–1250. 10.1111/apm.12295 25051890

[B41] Lade H. Park J. H. Chung S. H. Kim I. H. Kim J. M. Joo H. S. (2019). Biofilm Formation by *Staphylococcus aureus* clinical isolates is differentially affected by glucose and sodium chloride supplemented culture media. *J. Clin. Med.* 8:1853. 10.3390/jcm8111853 31684101 PMC6912320

[B42] Lee J. H. Kim Y. G. Ryu S. Y. Lee J. (2016). Calcium-chelating alizarin and other anthraquinones inhibit biofilm formation and the hemolytic activity of *Staphylococcus aureus*. *Sci. Rep.* 6:19267. 10.1038/srep19267 26763935 PMC4725881

[B43] Lee J. H. Park J. H. Cho H. S. Joo S. W. Cho M. H. Lee J. (2013). Anti-biofilm activities of quercetin and tannic acid against *Staphylococcus aureus*. *Biofouling* 29 491–499. 10.1080/08927014.2013.788692 23668380

[B44] Lee K. Lee J. H. Ryu S. Y. Cho M. H. Lee J. (2014). Stilbenes reduce *Staphylococcus aureus* hemolysis, biofilm formation, and virulence. *Foodborne Pathog. Dis.* 11 710–717. 10.1089/fpd.2014.1758 25007234

[B45] Lim Y. Jana M. Luong T. T. Lee C. Y. (2004). Control of glucose- and NaCl-induced biofilm formation by rbf in *Staphylococcus aureus*. *J. Bacteriol.* 186 722–729. 10.1128/jb.186.3.722-729.2004 14729698 PMC321492

[B46] Liu H. Sadygov R. G. Yates J. R. I. I. I. (2004). A model for random sampling and estimation of relative protein abundance in shotgun proteomics. *Anal. Chem.* 76 4193–4201. 10.1021/ac0498563 15253663

[B47] Liu Q. Yeo W. S. Bae T. (2016). The SaeRS Two-Component System of *Staphylococcus aureus*. *Genes* 7:81. 10.3390/genes7100081 27706107 PMC5083920

[B48] López D. Vlamakis H. Kolter R. (2010). Biofilms. *Cold Spring Harb. Perspec. Biolo.* 2:a000398. 10.1101/cshperspect.a000398 20519345 PMC2890205

[B49] Mah T.-F. (2012). Biofilm-specific antibiotic resistance. *Future Microbiol.* 7 1061–1072. 10.2217/fmb.12.76 22953707

[B50] Mamedov N. A. Egamberdieva D. (2019). “Phytochemical constituents and pharmacological effects of licorice: a review,” in *Plant and Human Health*, eds Hakeem K. R. Ozturk M. (Berlin: Springer), 1–21. 10.1007/978-3-030-04408-4_1

[B51] Neopane P. Nepal H. P. Shrestha R. Uehara O. Abiko Y. (2018). In vitro biofilm formation by *Staphylococcus aureus* isolated from wounds of hospital-admitted patients and their association with antimicrobial resistance. *Int. J. Gen. Med.* 11 25–32. 10.2147/IJGM.S153268 29403304 PMC5779313

[B52] Paharik A. E. Horswill A. R. (2016). The Staphylococcal biofilm: adhesins. regulation, and host response. *Microbiol. Spectr.* 4:2. 10.1128/microbiolspec.VMBF-0022-2015 27227309 PMC4887152

[B53] Phuong N. T. M. Van Quang N. Mai T. T. Anh N. V. Kuhakarn C. Reutrakul V. (2017). Antibiofilm activity of α-mangostin extracted from *Garcinia mangostana* L. against *Staphylococcus aureus*. *Asian Pac. J. Trop. Med.* 10 1154–1160. 10.1016/j.apjtm.2017.10.022 29268971

[B54] Pournajaf A. Ardebili A. Goudarzi L. Khodabandeh M. Narimani T. Abbaszadeh H. (2014). PCR-based identification of methicillin-resistant *Staphylococcus aureus* strains and their antibiotic resistance profiles. *Asian Pac. J. Trop. Biomed.* 4 S293–S297. 10.12980/APJTB.4.2014C423 25183100 PMC4025288

[B55] Reffuveille F. Josse J. Vallé Q. Gangloff C. M. Gangloff S. C. (2017). “*Staphylococcus aureus* bioflms and their impact on the medical field,” in *The Rise of Virulence and Antibiotic Resistance in Staphylococcus Aureus*, ed. Enany S. (London: IntechOpen), 187–214. 10.5772/66380

[B56] Resch A. Rosenstein R. Nerz C. Götz F. (2005). Differential gene expression profiling of *Staphylococcus aureus* cultivated under biofilm and planktonic conditions. *Appl. Environ. Microbiol.* 71 2663–2676. 10.1128/AEM.71.5.2663-2676.2005 15870358 PMC1087559

[B57] Rohinishree Y. S. Negi P. S. (2016). Effect of licorice extract on cell viability, biofilm formation and exotoxin production by *Staphylococcus aureus*. *J. Food Sci. Technol.* 53 1092–1100. 10.1007/s13197-015-2131-6 27162389 PMC4837708

[B58] Rubini D. Banu S. F. Nisha P. Murugan R. Thamotharan S. Percino M. J. (2018). Essential oils from unexplored aromatic plants quench biofilm formation and virulence of Methicillin resistant *Staphylococcus aureus*. *Microb. Pathog.* 122 162–173. 10.1016/j.micpath.2018.06.028 29920307

[B59] Sánchez E. Rivas Morales C. Castillo S. Leos-Rivas C. García-Becerra L. Mizael Ortiz Martínez D. (2016). Antibacterial and Antibiofilm Activity of Methanolic Plant Extracts against Nosocomial Microorganisms. *Evid. Based Complement. Altern. Med.* 2016 1–8. 10.1155/2016/1572697 27429633 PMC4939345

[B60] Santajit S. Indrawattana N. (2016). Mechanisms of Antimicrobial Resistance in ESKAPE Pathogens. *Biomed Res. Int.* 2016:2475067. 10.1155/2016/2475067 27274985 PMC4871955

[B61] Selvaraj A. Jayasree T. Valliammai A. Pandian S. K. (2019). Myrtenol Attenuates MRSA biofilm and virulence by suppressing sarA expression dynamism. *Front. Microbiol.* 10:2027. 10.3389/fmicb.2019.02027 31551964 PMC6737500

[B62] Sharma D. Misba L. Khan A. U. (2019). Antibiotics versus biofilm: an emerging battleground in microbial communities. *Antimicrob. Resist. Infect. Control* 8:76. 10.1186/s13756-019-0533-3 31131107 PMC6524306

[B63] Shukla S. K. Rao T. S. (2017). *Staphylococcus aureus* biofilm removal by targeting biofilm-associated extracellular proteins. *Indian J. Med. Res.* 146 S1–S8. 10.4103/ijmr.IJMR_410_15 PMC5735565 29205189

[B64] Simmler C. Pauli G. F. Chen S. N. (2013). Phytochemistry and biological properties of glabridin. *Fitoterapia* 90 160–184. 10.1016/j.fitote.2013.07.003 23850540 PMC3795865

[B65] Singh V. Pal A. Darokar M. P. (2015). A polyphenolic flavonoid glabridin: oxidative stress response in multidrug-resistant *Staphylococcus aureus*. *Free Radic. Biol. Med.* 87 48–57. 10.1016/j.freeradbiomed.2015.06.016 26117328

[B66] Singh V. K. Utaida S. Jackson L. S. Jayaswal R. K. Wilkinson B. J. Chamberlain N. R. (2007). Role for dnaK locus in tolerance of multiple stresses in *Staphylococcus aureus*. *Microbiology* 153 3162–3173. 10.1099/mic.0.2007/009506-0 17768259

[B67] Speziale P. Pietrocola G. Foster T. J. Geoghegan J. A. (2014). Protein-Based biofilm matrices in staphylococci. *Front. Cell. Infect. Microbiol.* 4:171. 10.3389/fcimb.2014.00171 25540773 PMC4261907

[B68] Stepanović S. Vuković D. Dakić I. Savić B. Švabić-Vlahović M. (2000). A modified microtiter-plate test for quantification of staphylococcal biofilm formation. *J. Microbiol. Methods* 40 175–179. 10.1016/s0167-7012(00)00122-6 10699673

[B69] Suwannakul S. Chaibenjawong P. (2017). Antibacterial Activities of *Glycyrrhiza gabra* Linn. (Licorice) Root Extract against *Porphyromonas gingivalis* rand its inhibitory effects on cysteine proteases and biofilms. *J. Dent. Indones.* 24 85–92. 10.14693/jdi.v24i3.1075

[B70] Tiwari H. K. Sen M. R. (2006). Emergence of vancomycin resistant *Staphylococcus aureus* (VRSA) from a tertiary care hospital from northern part of India. *BMC Infect. Dis.* 6:156. 10.1186/1471-2334-6-156 17067393 PMC1634751

[B71] Toledo-Arana A. Merino N. Vergara-Irigaray M. Débarbouillé M. Penadés J. R. Lasa I. (2005). *Staphylococcus aureus* Develops an Alternative, ica-Independent Biofilm in the Absence of the arlRS Two-component system. *J. Bacteriol.* 15 5318–5329. 10.1128/JB.187.15.5318-5329.2005 16030226 PMC1196035

[B72] Tong S. Y. Davis J. S. Eichenberger E. Holland T. L. Fowler V. G. (2015). *Staphylococcus aureus* infections: epidemiology, pathophysiology, clinical manifestations, and management. *Clin. Microbiol. Rev.* 28 603–661. 10.1128/CMR.00134-14 26016486 PMC4451395

[B73] Vuong C. Saenz H. L. Gotz F. Otto M. (2000). Impact of the agr quorum sensing system on adherence to polystyrene in *Staphylococcus aureus*. *J. Infect. Dis.* 182 1688–1693. 10.1086/317606 11069241

[B74] Wang L. Yang R. Yuan B. Liu Y. Liu C. (2015). The antiviral and antimicrobial activities of licorice, a widely used Chinese herb. *Acta Pharm. Sin. B* 5 310–315. 10.1016/j.apsb.2015.05.005 26579460 PMC4629407

[B75] Wang Y. Yi L. Zhang Z. Fan H. Cheng X. Lu C. (2014). Biofilm Formation. Host-Cell Adherence, and Virulence Genes Regulation of *Streptococcus suis* in Response to Autoinducer-2 Signaling. *Curr. Microbiol.* 68 575–580. 10.1007/s00284-013-0509-0 24370626

[B76] Wolz C. Pöhlmann-Dietze P. Steinhuber A. Chien Y. T. Manna A. van Wamel W. (2000). Agr-independent regulation of fibronectin binding protein(s) by the regulatory locus sar in *Staphylococcus aureus*. *Mol. Microbiol.* 36 230–243. 10.1046/j.1365-2958.2000.01853.x 10760180

[B77] Yarwood J. M. Bartels D. J. Volper E. M. Greenberg E. P. (2004). Quorum sensing in *Staphylococcus aureus* biofilms. *J. Bacteriol.* 186 1838–1850. 10.1128/jb.186.6.1838-1850.2004 14996815 PMC355980

[B78] Ythier M. Resch G. Waridel P. Panchaud A. Gfeller A. Majcherczyk P. (2012). Proteomic and transcriptomic profiling of *Staphylococcus aureus* surface LPXTG-proteins: correlation with agr genotypes and adherence phenotypes. *Mol. Cell Proteomics* 11 1123–1139. 10.1074/mcp.M111.014191 22843989 PMC3494191

[B79] Zimaro T. Thomas L. Marondedze C. Garavaglia B. S. Gehring C. Ottado J. (2013). Insights into xanthomonas axonopodis pv. citri biofilm through proteomics. *BMC Microbiol.* 13:186. 10.1186/1471-2180-13-186 23924281 PMC3750573

[B80] Zipfel C. (2008). Pattern-recognition receptors in plant innate immunity. *Curr. Opin. Immunol.* 20 10–16. 10.1016/j.coi.2007.11.003 18206360

