# Supplementary material for: Glabridin Averts Biofilms Formation in Methicillin-Resistant Staphylococcus aureus by Modulation of the Surfaceome
Source: Front Microbiol. 2020 Sep 17;11:1779. doi: 10.3389/fmicb.2020.01779 (PMC7534511; doi:10.3389/fmicb.2020.01779)
Supplement: Supplementary file 1 [file Data_Sheet_1.docx]

Supplementary Material


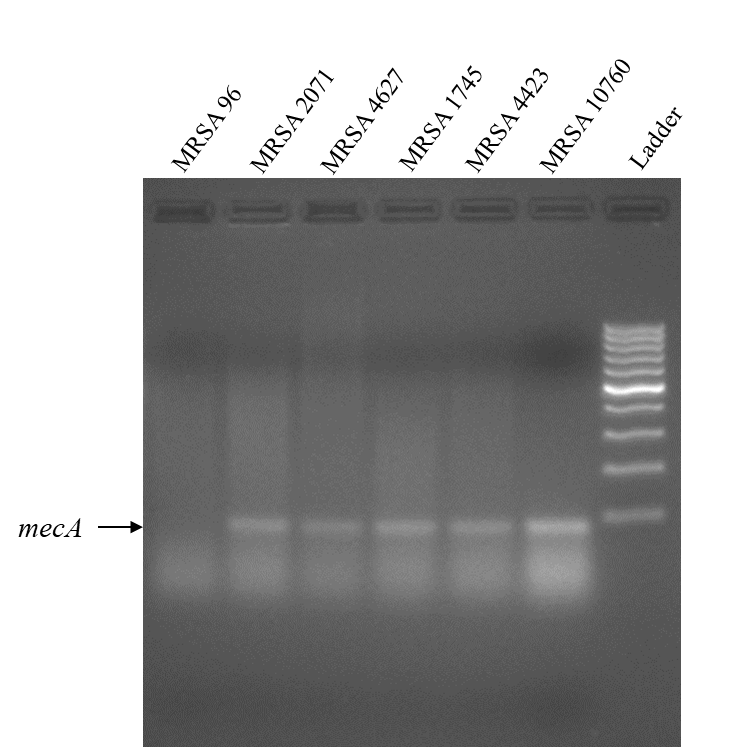


**Figure S1.** 2.5% agarose gel showing *mecA* amplicon (79 bp size) from MRSA clinical isolates (lane 2 to 5). *S. aureus* MTCC 96 was used as a control that lacks the *mecA* gene (lane 1). Last lane is showing a 100bp ladder (GeNei, Invitrogen).

| **Protein Name** | **Gene name/ gene number (*S. aureus* N315)** | **Primer Sequence (5' -3')** |
| --- | --- | --- |
| FnbB | *fnbB*/ SA2290 | F - CCAACACCGCCAACACCAGA  R - TGGTGTTTCCGGCTCACTTGG |
| FnbA | *fnbA*/ SA2291 | F - AACGCCACCAACACCAGAGG  R - GCGGTGTTGGTGTTTCTGGC |
| Sbi | *sbi*/ SA2206 | F - GCACCACACGATAAATCAGCAGC  R – ACACGCTCATCATGACGAACGA |
| FKLRK-domain protein | hypothetical/ SA0394 | F - TCCGATGGTGTTTGGATGCCT  R - TGCTTCATCTTCATCATCAGGTGCA |
| LPXTG-domain protein | hypothetical/ SA0022 | F - CAAGTCTCGAACGCGGCTGA  R - TGGTTGCCGCTTCTTCACCA |
| 5’ Nucleotidase | hypothetical/ SA0295 | F - AGCGGTTACAGTTTCAGCACCA  R - TTTGATACCAAGCCACTGCCA |
| AgrA | *agrA*/ SA1844 | F - TGCCCTCGCAACTGATAATCC  R - CCAACTGGGTCATGCTTACGA |
| SarA | *sarA*/ SA0573 | F - CGTAATGAGCATGATGAAAGAACTG  R - CGTTGTTTGCTTCAGTGATTCGT |
| SaeR | *saeR*/ SA0661 | F - TCCAAGGGAACTCGTTTTACGT  R - CGCATAGGGACTTCGTGACCA |
| ArlR [S] | hypothetical/ SA1248 | F - GCTGGGCTTGATTACGGTGC  R - AAGCGTTCCGTTGACATCGA |
| EF-Tu | *tufA*/ SA0506 | F - ACGTGGTGTTGCTCGTGAAGA  R - GAGTGTGACGTCCACCTTCGT |
| EF-G | *fusA* /SA0505 | F - ACGTAGGTGCTCCAATGGTTTCA  R - CCTGCGCCTGTTTCGTTTGG |
| DnaK | *dnaK*/ SA1409 | F - AGCAATGAAAGACGCTGGCT  R - GCCACTACTTCGTCCGGGTT |
| Pyruvate Kinase | *pykA*/ SA1520 | F - TGCTGGTATGAACGTTGCACGA  R - GCGTACGAATTTCTGGACCTTTCG |
| GAPDH | *gapA*/ SA0727 | F - GGTGACAAACGTCGTGCTCG  R - GGAACACGTTGTGCACCACC |
| RpoD | *rpoD*/ SA1390 | F - AGACAAGCAATCACTCGTGC  R - GGTGCTGGATCTCGACCTAA |
| 16s rRNA | *16S rRNA*/ SArRNA01 | F - AACGCCGCGTGAGTGATGAA  R - ACGTATTACCGCGGCTGCTG |

**Table S1. List of primers used in the qRT-PCR of this study**

**Table S2. Determination of minimum inhibitory concentrations (Micro-broth dilution assay)**

Minimum inhibitory concentrations (µg/ml) of different antibiotics against MRSA clinical isolates through micro-broth dilution assay. As per Clinical and Laboratory Standards Institute guidelines (CLSI-2018).

| **Antibiotics** | | ***S. aureus* strains** | | | | | |
| --- | --- | --- | --- | --- | --- | --- | --- |
|  |  | MTCC 96 | SA 2071 | SA 4627 | SA 1745 | SA 4423 | SA 10760 |
| Polyphenol | Glabridin | 12.5 | 12.5 | 12.5 | 12.5 | 12.5 | 12.5 |
| β-lactam group | Oxacillin | 3.9 | 1000 | 1000 | 1000 | 1000 | 1000 |
|  | Penicillin | 0.195 | 1000 | 500 | 1000 | 500 | 1000 |
|  | Carbenicillin | 3.9 | 500 | 500 | 500 | 500 | 500 |
|  | Ampicillin | 0.195 | 1000 | 1000 | 1000 | 1000 | 500 |
|  | Cephalosporin | 7.8 | 1000 | 1000 | 1000 | 1000 | 1000 |
|  | Cefoxitin | 1.56 | 500 | 250 | 500 | 250 | 500 |
| Glycopeptides | Vancomycin | 0.78 | 6.25 | 3.12 | 6.25 | 3.12 | 3.12 |
|  | Teicoplanin | 0.78 | 3.12 | 1.56 | 3.12 | 3.12 | 6.25 |
| Lipopeptides | Daptomycin | 1.56 | 3.12 | 1.56 | 3.12 | 1.56 | 1.56 |
|  | Colistin | 7.8 | 125 | 250 | 250 | 250 | 125 |
|  | Bacitracin | 7.8 | 125 | 31.25 | 125 | 125 | 125 |
| Aminoglycosides | Amikacin | 1.56 | 250 | 250 | 250 | 250 | 250 |
| Macrolides | Erythromycin | 0.78 | 500 | 500 | 500 | 500 | 250 |
| Tetracyclines | Tetracycline | 0.78 | 62.5 | 31.2 | 62.5 | 62.5 | 62.5 |
| Fluoroquinolones | Ciprofloxacin | 0.78 | 250 | 125 | 250 | 250 | 125 |
|  | Norfloxacin | 0.39 | 500 | 500 | 500 | 500 | 500 |
|  | NalidixicAcid | 1.56 | 250 | 250 | 250 | 500 | 250 |
| Folate inhibitor | Trimethopim | 1.56 | 1000 | 1000 | 1000 | 1000 | 1000 |
| Oxazolidinones | Linezolid | 1.56 | 3.12 | 1.56 | 3.12 | 1.56 | 1.56 |

Values shown in the table are mean of three separate experiments performed in triplicates.

To classify the antibacterial effect we adopted the following scale suggested by Aires et al. (2016): strong if MIC values ≤100 𝜇g⋅mL^−1^, moderate if 100 < MIC ≤ 500 𝜇g⋅mL^−1^, weak (+) if 500 < MIC ≤ 1000 𝜇g⋅mL^−1^, and null if MIC > 1000 𝜇g⋅mL^−1^.

**Table S3. Reported function of some major surface proteins identified in the biofilm-matrix (ECM) of clinical isolate MRSA 4423.**

Full set of proteins identified in ECM are provided below in the Table S3.

| **Biofilm related proteins identified in ECM complex** | | **Moonlighting proteins identified in ECM complex** | |
| --- | --- | --- | --- |
| **Protein name** | **Role in the cell surface of *S. aureus*** | **Protein name** | **Primary function/ Moonlight role** |
| Fibronectins-binding proteins (FnbA, FnbB) | Adherence to human airway epithelium. (Mongodin et al. 2002). | EF-Tu | A translation elongation factor/Adhesion binds to fibronectin protein in *M. pneumoniae.* (Siciliano et al., 2008; Dallo et al., 2002). |
| Immunoglobulin-binding protein (Sbi) | Multifunctional immune evasion factor. (Smith et al., 2012). | EF-G | An elongation factor in protein synthesis/ Adhesin, binds salivary mucin MUC7 in *Streptococcus gordonii.* (Kesimer et al., 2009). |
| SdrD/ hydrolase | Surface proteins that promote adherence of *S. aureus* to human desquamated nasal epithelial cells. (Corrigan et al., 2009). | DnaK | Heat shock protein/ Plasminogen binding protein (Xolalpa et al., 2007). |
| Putative surface protein/ FKLRK-domain Protein | Putative surface protein. | Pyruvate kinase (PK) | Glycolysis enzyme/ Binding to invertase, a hyperglycosylated mannoprotein from *Saccharomyces cerevisiae.* (Katakura et al., 2010) |
| Atl (amidase domain) | Autolysin, Atl, of *S. aureus* plays a variety of roles impaired in biofilm formation. Atl binds with fibronectin, thrombospondin 1, vitronectin and heat shock cognate protein Hsc70. (Oshida, T. and Tomasz, A. 1992). | Phosphoglycerate kinase (PGK) | Carbohydrate degradation, glycolysis/ binds plasminogen (Fulde et al., 2013). |
| MAP domain protein | It is a cell-wall associated protein, capable of binding to different extracellular matrix glycoploteins and plasma proteins to the cell surface of *S. aureus* (Harraghy et al., 2005) | GAPDH | Catalyzes the sixth step of glycolysis/Adhesin for mucin, fibronectin and plasminogen (Alvarez et al., 2003; Kinoshita et al., 2008). |

**Table S4. List of proteins identified by nano-LC-ESI-QTOF in biofilm matrix (ECM) of MRSA clinical isolate MRSA 4423**

| **S.N. (#)** | **Spectra (#)** | **Total Protein Spectral Intensity** | **Protein Name** | **Protein MW (Da)** | **Protein pI** | **Database Accession #** | **% AA Coverage** | **MS/MS Search Score** |
| --- | --- | --- | --- | --- | --- | --- | --- | --- |
| 1 | 77 | 7.13E+07 | bifunctional autolysin | 137420.6 | 9.62 | AKR50887.1 | 32.6 | 344.58 |
| 2 | 50 | 7.41E+07 | glycerol phosphate lipoteichoic acid synthase | 74398.7 | 9.04 | AHZ98610.1 | 31.1 | 191.91 |
| 3 | 22 | 7.54E+06 | Pbp2a | 76101.1 | 8.7 | ADV68968.1 | 22.9 | 144.27 |
| 4 | 25 | 2.74E+07 | penicillin-hydrolyzing class A beta-lactamase BlaZ | 31508.8 | 9.58 | OWU41412.1 | 24.5 | 86.23 |
| 5 | 43 | 3.30E+07 | CHAP domain-containing protein | 29383.7 | 9.1 | AKR52281.1 | 53.5 | 107.18 |
| 6 | 28 | 3.64E+07 | immunoglobulin-binding protein sbi | 50069.4 | 9.4 | AQQ85131.1 | 22.9 | 101.66 |
| 7 | 30 | 5.19E+07 | N-acetylmuramoyl-L-alanine amidase | 69279.1 | 5.96 | AIA29113.1 | 22.4 | 96.43 |
| 8 | 20 | 1.90E+07 | elongation factor Tu | 43159.9 | 4.74 | AHZ98444.1 | 27.4 | 80.39 |
| 9 | 10 | 5.62E+06 | fibronectin-binding protein | 111707.3 | 4.64 | AIA28975.1 | 17.4 | 75.79 |
| 10 | 20 | 2.82E+07 | 5'-nucleotidase | 33351.3 | 9.49 | AIA26892.1 | 27 | 66.4 |
| 11 | 13 | 8.84E+06 | beta-lactam sensor/signal transducer MecR1 | 68901.1 | 8.69 | OWU43946.1 | 12.8 | 62.33 |
| 12 | 10 | 5.55E+06 | glyceraldehyde-3-phosphate dehydrogenase | 36394.3 | 4.89 | AHZ98665.1 | 36.6 | 59.15 |
| 13 | 9 | 4.57E+06 | enolase | 47173.1 | 4.55 | AHZ98669.1 | 20.7 | 49.81 |
| 14 | 19 | 3.00E+07 | N-acetylmuramoyl-L-alanine amidase | 36039.3 | 9.71 | AHZ98361.1 | 21.4 | 48.9 |
| 15 | 7 | 9.77E+06 | succinyl-diaminopimelate desuccinylase | 40346.3 | 9.45 | AHZ99851.1 | 18.2 | 47 |
| 16 | 6 | 3.07E+06 | hypothetical protein EX97_05040 | 45684.4 | 6.02 | AIA27548.1 | 15.5 | 39.72 |
| 17 | 10 | 3.81E+06 | ferrichrome ABC transporter substrate-binding protein | 34067.7 | 9.19 | AIA00102.1 | 16.2 | 38.44 |
| 18 | 12 | 1.36E+07 | fibrinogen-binding protein | 18764.2 | 9.82 | AIA27644.1 | 35.7 | 35.77 |
| 19 | 7 | 2.55E+06 | YSIRK domain-containing triacylglycerol lipase Lip2/Geh | 76416.4 | 9.05 | AIA26905.1 | 7.6 | 30.88 |
| 20 | 4 | 1.71E+06 | fructose-1,6-bisphosphate aldolase | 32996.9 | 4.92 | AIA00424.1 | 18.5 | 29.72 |
| 21 | 6 | 2.36E+06 | superantigen-like protein SSL11 | 25365.2 | 8.53 | AKR45235.1 | 15.5 | 28.12 |
| 22 | 9 | 1.93E+07 | inhibitor | 13066.8 | 9.33 | AHZ99787.1 | 26.7 | 27.4 |
| 23 | 6 | 4.47E+06 | gamma-hemolysin subunit B | 38787.5 | 8.66 | AHZ99850.1 | 10.6 | 26.19 |
| 24 | 3 | 4.94E+05 | gamma-hemolysin subunit B | 36712.3 | 9.04 | AIA00236.1 | 9.2 | 25.88 |
| 25 | 2 | 1.31E+06 | delta-hemolysin | 2978.5 | 8.19 | KMR09253.1 | 80.7 | 23.32 |
| 26 | 3 | 1.27E+06 | alkyl hydroperoxide reductase | 21147.4 | 4.88 | AHZ98297.1 | 19.5 | 20.44 |
| 27 | 3 | 1.12E+06 | signal peptide protein/ FKLRK protein | 56545.7 | 4.79 | AIA26995.1 | 9.3 | 19.96 |
| 28 | 2 | 3.42E+05 | elongation factor G | 76896.3 | 4.8 | AHZ98443.1 | 6.4 | 19.3 |
| 29 | 2 | 5.84E+05 | peroxidase | 18119.1 | 4.73 | AIA28240.1 | 31 | 19.21 |
| 30 | 3 | 5.81E+05 | hypothetical protein ER16_11670/ CHAP-domain | 17455.5 | 5.77 | AIA00120.1 | 28.3 | 17.83 |
| 31 | 2 | 1.29E+06 | matrix-binding protein | 1145089.3 | 5.98 | AHZ99240.1 | 0.2 | 17.53 |
| 32 | 1 | 4.13E+03 | ribulokinase | 61179.9 | 5.80 | ALK41039.1 | 8 | 17.46 |
| 33 | 2 | 7.88E+05 | beta-class phenol-soluble modulin | 4496.1 | 4.78 | AKR47248.1 | 40.9 | 16.46 |
| 34 | 2 | 7.11E+05 | protein map | 65573.1 | 9.85 | AIA28457.1 | 5.3 | 16.43 |
| 35 | 2 | 1.29E+06 | matrix-binding protein/ Ebh | 1122530.7 | 5.87 | AIA27922.1 | 0.2 | 15.87 |
| 36 | 2 | 3.25E+06 | cysteine synthase | 33090.2 | 5.39 | AHZ98409.1 | 16.1 | 15.45 |
| 37 | 3 | 1.35E+06 | superoxide dismutase | 22710.9 | 5.08 | AHZ99416.1 | 10.5 | 14.66 |
| 38 | 2 | 1.50E+06 | 30S ribosomal protein S15 | 10608.1 | 10.5 | AHZ99055.1 | 31.4 | 14.22 |
| 39 | 1 | 1.13E+05 | fibrinogen-binding protein | 12592.4 | 10.41 | AHZ98937.1 | 11.9 | 13.91 |
| 40 | 2 | 9.65E+03 | DUF2648 domain-containing protein | 3846.5 | 9.6 | AUM84643.1 | 27.2 | 13.84 |
| 41 | 1 | 8.84E+05 | glycerate kinase | 40944.9 | 4.96 | AIA00248.1 | 8.4 | 13.71 |
| 42 | 2 | 6.90E+05 | Isoleucyl-tRNA synthetase (plasmid) | 119507.4 | 7.58 | ADA62077.1 | 3.9 | 13.5 |
| 43 | 3 | 1.90E+06 | dihydrolipoamide dehydrogenase | 49621.6 | 4.95 | AHZ98882.1 | 4.4 | 13.36 |
| 44 | 1 | 1.18E+06 | tail protein | 20517.4 | 4.81 | AKK58988.1 | 15.6 | 13.32 |
| 45 | 2 | 8.59E+05 | enterotoxin I | 27806.7 | 6.97 | AIA27393.1 | 10.3 | 12.9 |
| 46 | 1 | 0.00E+00 | Asp-tRNA(Asn)/Glu-tRNA(Gln) amidotransferase GatCAB subunit A | 53019.1 | 5.02 | OWU40549.1 | 8.4 | 12.89 |
| 47 | 1 | 1.04E+04 | ATP-dependent DNA helicase | 141221.2 | 5.16 | AHZ98759.1 | 2.8 | 12.61 |
| 48 | 1 | 4.73E+05 | hypothetical protein EV30_15010 | 8613.2 | 9.77 | KMR25920.1 | 18 | 12.59 |
| 49 | 1 | 8.45E+05 | hypothetical protein EU79_14995, partial | 7800.7 | 9.39 | KMR19606.1 | 42.4 | 12.49 |
| 50 | 1 | 1.67E+05 | 50S ribosomal protein L4 | 22464.3 | 9.9 | AIA00070.1 | 13.5 | 12.48 |
| 51 | 1 | 2.94E+04 | membrane protein | 29765.5 | 9.89 | AHZ98715.1 | 18.9 | 12.44 |
| 52 | 1 | 2.91E+05 | hypothetical protein ER16_13420 | 19595.9 | 9.54 | AIA00455.1 | 6.8 | 12.32 |
| 53 | 2 | 2.73E+06 | alanine dehydrogenase | 40104.5 | 5.58 | AHZ99557.1 | 7.7 | 12.26 |
| 54 | 1 | 1.16E+06 | nitrite reductase | 89553.4 | 5.21 | AIA00215.1 | 3.1 | 12 |
| 55 | 1 | 2.49E+05 | amidophosphoribosyltransferase | 54637.9 | 6.11 | AHZ98858.1 | 2.6 | 11.8 |
| 56 | 3 | 1.09E+06 | phenol-soluble modulin PSM-alpha-4 | 2171.8 |  | AUM84795.1 | 40 | 11.38 |
| 57 | 1 | 2.94E+05 | magnesium transporter MgtE | 51579.7 | 4.1 | KMR72263.1 | 5.8 | 11.34 |
| 58 | 1 | 2.22E+05 | 50S ribosomal protein L25 | 23787.3 | 4.39 | AHZ98397.1 | 7.3 | 11.3 |
| 59 | 1 | 0.00E+00 | XRE family transcriptional regulator (plasmid) | 10414 | 7.81 | AQR26686.1 | 19.7 | 11 |
| 60 | 9 | 1.01E+07 | argininosuccinate lyase, partial | 9272.8 | 8.82 | KMR91728.1 | 16.2 | 10.99 |
| 61 | 1 | 9.08E+05 | transglycosylase | 24233.8 | 6.11 | AIA00385.1 | 16.7 | 10.81 |
| 62 | 1 | 8.78E+04 | dephospho-CoA kinase | 23622.4 | 4.75 | AHZ99537.1 | 10.6 | 10.77 |
| 63 | 1 | 1.06E+05 | restriction endonuclease subunit R (plasmid) | 108954.5 | 5.35 | ALK37966.1 | 2 | 10.72 |

**Table S5: List of proteins identified by nano-LC-ESI-QTOF on the cell surface of clinical isolates MRSA 4423.** Spectral count/or intensity represents an abundance of a particular protein under different conditions; control (A), 1% glucose treatment (B) and 1% glucose + glabridin (1/4 MIC= 3.25 ug/ml) (C).

| **S.N.** | **A** | **B** | **C** | **Protein name** | **Protein MW (Da)** | **Protein pI** | **Database Accession #** | **%AA Coverage** | **MS/MS Search Score** | A |
| --- | --- | --- | --- | --- | --- | --- | --- | --- | --- | --- |
|  |  |  |  |  |  |  |  |  |  |  |
|  | **# spectra/ total intensity** | **# spectra/ total intensity** | **# spectra/ total intensity** |  |  |  |  |  |  |  |
|  |  |  |  |  |  |  |  |  |  |  |
| 1 | 0 | 69 | 62 | mannosyl-glycoprotein endo-beta-N-acetylglucosamidase | 137391 | 9.62 | [ALK38880.1](http://www.ncbi.nlm.nih.gov/protein/ALK38880.1) | [47.6](http://desktop-0t1rtqn/millbin/msdigest.cgi?missed_cleavages=2&msparams_dir=msparams_mill/&hide_protein_sequence=2&database=NCBIgb_SA300sequence.fasta&seqdb_dir=D:\SeqDB\&enzyme=Trypsin&access_method=Accession+Number&accession_num=ALK38880.1&coverage_map=0+56+25+48+30+57+14+8+11+22+135+7+54+24+12+31+10+24+11+6+51+29+12+10+16+34+11+6+10+63+34+12+49+23+9+20+24+111+29+16+20+37+23+14+8) | 484.65 | 0 |
|  | 0.00E+00 | 6.72E+07 | 5.05E+07 |  |  |  |  |  |  |  |
| 2.1 | 128 | 33 | 55 | elongation factor Tu | 43159.9 | 4.74 | [AIA27105.1](http://www.ncbi.nlm.nih.gov/protein/AIA27105.1) | [73.6](http://desktop-0t1rtqn/millbin/msdigest.cgi?missed_cleavages=2&msparams_dir=msparams_mill/&hide_protein_sequence=2&database=NCBIgb_SA300sequence.fasta&seqdb_dir=D:\SeqDB\&enzyme=Trypsin&access_method=Accession+Number&accession_num=AIA27105.1&coverage_map=0+25+50+15+27+7+53+11+36+14+42+9+30+15+40+8+12) | 317.16 | 128 |
|  | 1.62E+09 | 8.02E+07 | 2.96E+08 |  |  |  |  |  |  |  |
| 2.2 | 3 | 0 | 3 | elongation factor Tu, partial | 8757.6 | 4.96 | [KMR26954.1](http://www.ncbi.nlm.nih.gov/protein/KMR26954.1) | [38.2](http://desktop-0t1rtqn/millbin/msdigest.cgi?missed_cleavages=2&msparams_dir=msparams_mill/&hide_protein_sequence=2&database=NCBIgb_SA300sequence.fasta&seqdb_dir=D:\SeqDB\&enzyme=Trypsin&access_method=Accession+Number&accession_num=KMR26954.1&coverage_map=0+11+15+11+16+28) | 21.88 | 3 |
|  | 1.17E+07 | 0.00E+00 | 1.66E+06 |  |  |  |  |  |  |  |
| 3.1 | 1 | 62 | 7 | fibronectin-binding protein A | 111806 | 4.64 | [KMS18095.1](http://www.ncbi.nlm.nih.gov/protein/KMS18095.1) | [42.6](http://desktop-0t1rtqn/millbin/msdigest.cgi?missed_cleavages=2&msparams_dir=msparams_mill/&hide_protein_sequence=2&database=NCBIgb_SA300sequence.fasta&seqdb_dir=D:\SeqDB\&enzyme=Trypsin&access_method=Accession+Number&accession_num=KMS18095.1&coverage_map=0+56+71+20+18+65+30+6+16+28+21+5+21+17+12+12+13+11+49+17+16+117+51+65+49+15+56+97+11+53) | 264.99 | 1 |
|  | 9.59E+04 | 7.30E+07 | 1.14E+06 |  |  |  |  |  |  |  |
| 3.2 | 1 | 3 | 1 | fibronectin-binding protein B | 104799 | 4.66 | [QBS27992.1](http://www.ncbi.nlm.nih.gov/protein/QBS27992.1) | [2.9](http://desktop-0t1rtqn/millbin/msdigest.cgi?missed_cleavages=2&msparams_dir=msparams_mill/&hide_protein_sequence=2&database=NCBIgb_SA300sequence.fasta&seqdb_dir=D:\SeqDB\&enzyme=Trypsin&access_method=Accession+Number&accession_num=QBS27992.1&coverage_map=0+478+17+381+11+63) | 20.05 | 1 |
|  | 9.59E+04 | 1.99E+06 | 1.17E+05 |  |  |  |  |  |  |  |
| 4 | 42 | 9 | 23 | elongation factor G | 76926.4 | 4.8 | [OWU45296.1](http://www.ncbi.nlm.nih.gov/protein/OWU45296.1) | [53.1](http://desktop-0t1rtqn/millbin/msdigest.cgi?missed_cleavages=2&msparams_dir=msparams_mill/&hide_protein_sequence=2&database=NCBIgb_SA300sequence.fasta&seqdb_dir=D:\SeqDB\&enzyme=Trypsin&access_method=Accession+Number&accession_num=OWU45296.1&coverage_map=0+11+12+16+20+17+18+3+24+33+28+3+21+5+17+17+18+2+26+11+13+48+52+52+16+20+60+43+20+10+23+34) | 240.98 | 42 |
|  | 2.57E+08 | 6.21E+06 | 1.99E+07 |  |  |  |  |  |  |  |
| 5 | 43 | 5 | 21 | pyruvate kinase | 63329.4 | 5.24 | [AIA28225.1](http://www.ncbi.nlm.nih.gov/protein/AIA28225.1) | [50.4](http://desktop-0t1rtqn/millbin/msdigest.cgi?missed_cleavages=2&msparams_dir=msparams_mill/&hide_protein_sequence=2&database=NCBIgb_SA300sequence.fasta&seqdb_dir=D:\SeqDB\&enzyme=Trypsin&access_method=Accession+Number&accession_num=AIA28225.1&coverage_map=0+22+22+15+14+13+17+76+16+24+24+48+34+3+14+7+24+51+60+19+54+3+16+9) | 240.03 | 43 |
|  | 9.94E+07 | 3.06E+06 | 1.50E+07 |  |  |  |  |  |  |  |
| 6 | 7 | 28 | 67 | penicillin-hydrolyzing class A beta-lactamase BlaZ | 31405.8 | 9.58 | [OWU47924.1](http://www.ncbi.nlm.nih.gov/protein/OWU47924.1) | [49.8](http://desktop-0t1rtqn/millbin/msdigest.cgi?missed_cleavages=2&msparams_dir=msparams_mill/&hide_protein_sequence=2&database=NCBIgb_SA300sequence.fasta&seqdb_dir=D:\SeqDB\&enzyme=Trypsin&access_method=Accession+Number&accession_num=OWU47924.1&coverage_map=0+33+13+20+36+9+17+16+22+29+26+14+26+20) | 237.92 | 7 |
|  | 1.55E+06 | 4.78E+07 | 2.12E+08 |  |  |  |  |  |  |  |
| 7 | 8 | 37 | 38 | PBP2a family beta-lactam-resistant peptidoglycan transpeptidase MecA | 76232.3 | 8.7 | [OWU35180.1](http://www.ncbi.nlm.nih.gov/protein/OWU35180.1) | [36.6](http://desktop-0t1rtqn/millbin/msdigest.cgi?missed_cleavages=2&msparams_dir=msparams_mill/&hide_protein_sequence=2&database=NCBIgb_SA300sequence.fasta&seqdb_dir=D:\SeqDB\&enzyme=Trypsin&access_method=Accession+Number&accession_num=OWU35180.1&coverage_map=0+111+8+6+14+15+23+12+10+21+10+18+18+33+18+6+9+10+41+12+23+19+33+15+22+59+16+87) | 223.76 | 8 |
|  | 1.34E+06 | 2.44E+07 | 3.13E+07 |  |  |  |  |  |  |  |
| 8 | 6 | 34 | 36 | peptidase | 76100.2 | 8.7 | [KMS44729.1](http://www.ncbi.nlm.nih.gov/protein/KMS44729.1) | [36.6](http://desktop-0t1rtqn/millbin/msdigest.cgi?missed_cleavages=2&msparams_dir=msparams_mill/&hide_protein_sequence=2&database=NCBIgb_SA300sequence.fasta&seqdb_dir=D:\SeqDB\&enzyme=Trypsin&access_method=Accession+Number&accession_num=KMS44729.1&coverage_map=0+110+8+6+14+15+23+12+10+21+10+18+18+33+18+6+9+10+41+12+23+19+33+15+22+59+16+87) | 216.54 | 6 |
|  | 1.02E+06 | 2.28E+07 | 2.90E+07 |  |  |  |  |  |  |  |
| 9 | 39 | 0 | 2 | formate acetyltransferase | 85316.8 | 5.31 | [AIA26805.1](http://www.ncbi.nlm.nih.gov/protein/AIA26805.1) | [45.2](http://desktop-0t1rtqn/millbin/msdigest.cgi?missed_cleavages=2&msparams_dir=msparams_mill/&hide_protein_sequence=2&database=NCBIgb_SA300sequence.fasta&seqdb_dir=D:\SeqDB\&enzyme=Trypsin&access_method=Accession+Number&accession_num=AIA26805.1&coverage_map=0+27+26+15+9+85+13+9+30+21+50+3+19+8+32+4+23+28+20+13+37+43+20+25+18+12+19+28+11+7+12+82) | 214.94 | 39 |
|  | 1.18E+08 | 0.00E+00 | 1.30E+06 |  |  |  |  |  |  |  |
| 10 | 19 | 16 | 40 | enolase | 47173.1 | 4.55 | [AIA27354.1](http://www.ncbi.nlm.nih.gov/protein/AIA27354.1) | [56.4](http://desktop-0t1rtqn/millbin/msdigest.cgi?missed_cleavages=2&msparams_dir=msparams_mill/&hide_protein_sequence=2&database=NCBIgb_SA300sequence.fasta&seqdb_dir=D:\SeqDB\&enzyme=Trypsin&access_method=Accession+Number&accession_num=AIA27354.1&coverage_map=0+16+36+14+39+17+12+8+36+20+15+55+35+9+50+10+22+40) | 194.35 | 19 |
|  | 2.39E+07 | 1.29E+07 | 6.07E+07 |  |  |  |  |  |  |  |
| 11 | 15 | 12 | 38 | glyceraldehyde-3-phosphate dehydrogenase | 36394.3 | 4.89 | [AIA27350.1](http://www.ncbi.nlm.nih.gov/protein/AIA27350.1) | [69.9](http://desktop-0t1rtqn/millbin/msdigest.cgi?missed_cleavages=2&msparams_dir=msparams_mill/&hide_protein_sequence=2&database=NCBIgb_SA300sequence.fasta&seqdb_dir=D:\SeqDB\&enzyme=Trypsin&access_method=Accession+Number&accession_num=AIA27350.1&coverage_map=0+20+26+7+13+7+13+30+77+7+15+19+64+11+27) | 187.99 | 15 |
|  | 3.13E+07 | 7.58E+06 | 6.42E+07 |  |  |  |  |  |  |  |
| 12 | 0 | 15 | 10 | glycerol phosphate lipoteichoic acid synthase | 74398.7 | 9.04 | [AIA27296.1](http://www.ncbi.nlm.nih.gov/protein/AIA27296.1) | [34.9](http://desktop-0t1rtqn/millbin/msdigest.cgi?missed_cleavages=2&msparams_dir=msparams_mill/&hide_protein_sequence=2&database=NCBIgb_SA300sequence.fasta&seqdb_dir=D:\SeqDB\&enzyme=Trypsin&access_method=Accession+Number&accession_num=AIA27296.1&coverage_map=0+232+13+37+56+65+58+1+27+26+39+29+14+3+19+27) | 173.88 | 0 |
|  | 0.00E+00 | 1.92E+07 | 7.28E+06 |  |  |  |  |  |  |  |
| 13 | 25 | 0 | 10 | molecular chaperone DnaK | 66472.3 | 4.67 | [KMS23456.1](http://www.ncbi.nlm.nih.gov/protein/KMS23456.1) | [32.4](http://desktop-0t1rtqn/millbin/msdigest.cgi?missed_cleavages=2&msparams_dir=msparams_mill/&hide_protein_sequence=2&database=NCBIgb_SA300sequence.fasta&seqdb_dir=D:\SeqDB\&enzyme=Trypsin&access_method=Accession+Number&accession_num=KMS23456.1&coverage_map=0+3+22+30+13+14+16+43+16+39+20+24+32+53+52+49+11+31+16+126) | 160.86 | 25 |
|  | 5.77E+07 | 0.00E+00 | 5.08E+06 |  |  |  |  |  |  |  |
| 14 | 25 | 1 | 7 | cysteine synthase | 33032.1 | 5.39 | [AIA27070.1](http://www.ncbi.nlm.nih.gov/protein/AIA27070.1) | [74.1](http://desktop-0t1rtqn/millbin/msdigest.cgi?missed_cleavages=2&msparams_dir=msparams_mill/&hide_protein_sequence=2&database=NCBIgb_SA300sequence.fasta&seqdb_dir=D:\SeqDB\&enzyme=Trypsin&access_method=Accession+Number&accession_num=AIA27070.1&coverage_map=0+22+13+13+8+6+26+3+13+5+16+7+58+4+50+12+23+8+23) | 147.61 | 25 |
|  | 3.87E+07 | 1.52E+05 | 2.87E+06 |  |  |  |  |  |  |  |
| 15 | 18 | 0 | 0 | threonyl-tRNA synthase | 74573 | 5.23 | [AIA28209.1](http://www.ncbi.nlm.nih.gov/protein/AIA28209.1) | [29.7](http://desktop-0t1rtqn/millbin/msdigest.cgi?missed_cleavages=2&msparams_dir=msparams_mill/&hide_protein_sequence=2&database=NCBIgb_SA300sequence.fasta&seqdb_dir=D:\SeqDB\&enzyme=Trypsin&access_method=Accession+Number&accession_num=AIA28209.1&coverage_map=0+14+20+7+33+171+29+91+12+24+20+27+23+6+21+28+10+80+24+5) | 147.4 | 18 |
|  | 3.49E+07 | 0.00E+00 | 0.00E+00 |  |  |  |  |  |  |  |
| 16 | 1 | 16 | 7 | hydrolase | 149704 | 4.12 | [KMR76067.1](http://www.ncbi.nlm.nih.gov/protein/KMR76067.1) | [13.8](http://desktop-0t1rtqn/millbin/msdigest.cgi?missed_cleavages=2&msparams_dir=msparams_mill/&hide_protein_sequence=2&database=NCBIgb_SA300sequence.fasta&seqdb_dir=D:\SeqDB\&enzyme=Trypsin&access_method=Accession+Number&accession_num=KMR76067.1&coverage_map=0+147+17+81+22+21+24+74+32+67+62+217+25+135+10+449) | 145.67 | 1 |
|  | 5.88E+05 | 3.64E+06 | 2.01E+06 |  |  |  |  |  |  |  |
| 17 | 21 | 0 | 2 | glucosamine--fructose-6-phosphate aminotransferase | 65962.4 | 4.93 | [AIA28646.1](http://www.ncbi.nlm.nih.gov/protein/AIA28646.1) | [37.1](http://desktop-0t1rtqn/millbin/msdigest.cgi?missed_cleavages=2&msparams_dir=msparams_mill/&hide_protein_sequence=2&database=NCBIgb_SA300sequence.fasta&seqdb_dir=D:\SeqDB\&enzyme=Trypsin&access_method=Accession+Number&accession_num=AIA28646.1&coverage_map=0+55+39+54+57+27+14+69+40+12+34+81+39+80) | 142.08 | 21 |
|  | 1.40E+07 | 0.00E+00 | 1.09E+05 |  |  |  |  |  |  |  |
| 18 | 12 | 0 | 10 | malate:quinone oxidoreductase | 56183.1 | 6.12 | [KMS00114.1](http://www.ncbi.nlm.nih.gov/protein/KMS00114.1) | [40.5](http://desktop-0t1rtqn/millbin/msdigest.cgi?missed_cleavages=2&msparams_dir=msparams_mill/&hide_protein_sequence=2&database=NCBIgb_SA300sequence.fasta&seqdb_dir=D:\SeqDB\&enzyme=Trypsin&access_method=Accession+Number&accession_num=KMS00114.1&coverage_map=0+3+36+68+13+55+14+4+32+9+20+7+26+26+13+15+13+6+10+15+11+68+14+20) | 140.79 | 12 |
|  | 3.69E+06 | 0.00E+00 | 2.69E+06 |  |  |  |  |  |  |  |
| 19 | 17 | 4 | 10 | phosphoglycerate kinase | 42670.1 | 5.17 | [KMR99061.1](http://www.ncbi.nlm.nih.gov/protein/KMR99061.1) | [39.1](http://desktop-0t1rtqn/millbin/msdigest.cgi?missed_cleavages=2&msparams_dir=msparams_mill/&hide_protein_sequence=2&database=NCBIgb_SA300sequence.fasta&seqdb_dir=D:\SeqDB\&enzyme=Trypsin&access_method=Accession+Number&accession_num=KMR99061.1&coverage_map=0+19+34+18+12+4+9+34+26+25+21+34+21+4+8+10+24+93) | 134.96 | 17 |
|  | 2.21E+07 | 6.00E+05 | 6.23E+06 |  |  |  |  |  |  |  |
| 20 | 4 | 42 | 50 | CHAP-domain containing protein | 30433.8 | 9.1 | [KMS46097.1](http://www.ncbi.nlm.nih.gov/protein/KMS46097.1) | [52](http://desktop-0t1rtqn/millbin/msdigest.cgi?missed_cleavages=2&msparams_dir=msparams_mill/&hide_protein_sequence=2&database=NCBIgb_SA300sequence.fasta&seqdb_dir=D:\SeqDB\&enzyme=Trypsin&access_method=Accession+Number&accession_num=KMS46097.1&coverage_map=0+108+53+10+15+4+17+10+58) | 130.25 | 4 |
|  | 1.04E+06 | 7.80E+07 | 8.40E+07 |  |  |  |  |  |  |  |
| 21 | 24 | 1 | 0 | ATP F0F1 synthase subunit beta | 51399.4 | 4.68 | [AIA28597.1](http://www.ncbi.nlm.nih.gov/protein/AIA28597.1) | [42.7](http://desktop-0t1rtqn/millbin/msdigest.cgi?missed_cleavages=2&msparams_dir=msparams_mill/&hide_protein_sequence=2&database=NCBIgb_SA300sequence.fasta&seqdb_dir=D:\SeqDB\&enzyme=Trypsin&access_method=Accession+Number&accession_num=AIA28597.1&coverage_map=0+5+12+57+17+26+30+14+27+19+15+2+64+6+36+140) | 118.15 | 24 |
|  | 2.86E+07 | 3.10E+04 | 0.00E+00 |  |  |  |  |  |  |  |
| 22 | 12 | 2 | 7 | 2-oxoisovalerate dehydrogenase | 35245.4 | 4.65 | [AIA27584.1](http://www.ncbi.nlm.nih.gov/protein/AIA27584.1) | [52.9](http://desktop-0t1rtqn/millbin/msdigest.cgi?missed_cleavages=2&msparams_dir=msparams_mill/&hide_protein_sequence=2&database=NCBIgb_SA300sequence.fasta&seqdb_dir=D:\SeqDB\&enzyme=Trypsin&access_method=Accession+Number&accession_num=AIA27584.1&coverage_map=0+15+24+77+28+19+12+3+17+5+53+27+38+7) | 116.91 | 12 |
|  | 5.45E+06 | 1.41E+06 | 1.83E+06 |  |  |  |  |  |  |  |
| 23 | 20 | 0 | 1 | 6'-aminoglycoside N-acetyltransferase (AAC(6')) / 2''-aminoglycoside phosphotransferase (plasmid) | 57253.5 | 4.75 | [ADA62071.1](http://www.ncbi.nlm.nih.gov/protein/ADA62071.1) | [29.2](http://desktop-0t1rtqn/millbin/msdigest.cgi?missed_cleavages=2&msparams_dir=msparams_mill/&hide_protein_sequence=2&database=NCBIgb_SA300sequence.fasta&seqdb_dir=D:\SeqDB\&enzyme=Trypsin&access_method=Accession+Number&accession_num=ADA62071.1&coverage_map=0+11+12+6+8+40+33+91+25+30+19+61+21+79+22+21) | 115.04 | 20 |
|  | 3.09E+07 | 0.00E+00 | 7.47E+04 |  |  |  |  |  |  |  |
| 24 | 0 | 21 | 16 | 5'-nucleotidase | 33351.3 | 9.49 | [AIA26892.1](http://www.ncbi.nlm.nih.gov/protein/AIA26892.1) | [30.7](http://desktop-0t1rtqn/millbin/msdigest.cgi?missed_cleavages=2&msparams_dir=msparams_mill/&hide_protein_sequence=2&database=NCBIgb_SA300sequence.fasta&seqdb_dir=D:\SeqDB\&enzyme=Trypsin&access_method=Accession+Number&accession_num=AIA26892.1&coverage_map=0+50+32+72+14+44+23+14+22+25) | 113.59 | 0 |
|  | 0.00E+00 | 7.32E+07 | 7.37E+07 |  |  |  |  |  |  |  |
| 25 | 0 | 23 | 0  0.00E+00 | immunoglobulin-binding protein sbi | 50117.5 | 9.4 | [KMR92723.1](http://www.ncbi.nlm.nih.gov/protein/KMR92723.1) | [23.1](http://desktop-0t1rtqn/millbin/msdigest.cgi?missed_cleavages=2&msparams_dir=msparams_mill/&hide_protein_sequence=2&database=NCBIgb_SA300sequence.fasta&seqdb_dir=D:\SeqDB\&enzyme=Trypsin&access_method=Accession+Number&accession_num=KMR92723.1&coverage_map=0+53+9+20+10+20+17+5+13+141+10+49+18+34+13+13+11) | 111.45 | 0 |
|  | 0.00E+00 | 5.53E+07 |  |  |  |  |  |  |  |  |
| 26 | 21 | 2 | 9 | cell division protein FtsZ | 41036.2 | 4.87 | [AIA27672.1](http://www.ncbi.nlm.nih.gov/protein/AIA27672.1) | [39.7](http://desktop-0t1rtqn/millbin/msdigest.cgi?missed_cleavages=2&msparams_dir=msparams_mill/&hide_protein_sequence=2&database=NCBIgb_SA300sequence.fasta&seqdb_dir=D:\SeqDB\&enzyme=Trypsin&access_method=Accession+Number&accession_num=AIA27672.1&coverage_map=1+15+14+25+26+39+3+19+14+13+23+45+154) | 109.88 | 21 |
|  | 2.78E+07 | 2.46E+05 | 2.98E+06 |  |  |  |  |  |  |  |
| 27 | 16 | 0 | 1 | 30S ribosomal protein S1 | 43286.5 | 4.51 | [AIA27961.1](http://www.ncbi.nlm.nih.gov/protein/AIA27961.1) | [32.9](http://desktop-0t1rtqn/millbin/msdigest.cgi?missed_cleavages=2&msparams_dir=msparams_mill/&hide_protein_sequence=2&database=NCBIgb_SA300sequence.fasta&seqdb_dir=D:\SeqDB\&enzyme=Trypsin&access_method=Accession+Number&accession_num=AIA27961.1&coverage_map=0+120+35+28+17+56+32+26+28+6+17+26) | 109.23 | 16 |
|  | 1.57E+07 | 0.00E+00 | 7.32E+04 |  |  |  |  |  |  |  |
| 28 | 12 | 2 | 6 | pyruvate dehydrogenase | 41381.9 | 4.9 | [AIA27583.1](http://www.ncbi.nlm.nih.gov/protein/AIA27583.1) | [45.6](http://desktop-0t1rtqn/millbin/msdigest.cgi?missed_cleavages=2&msparams_dir=msparams_mill/&hide_protein_sequence=2&database=NCBIgb_SA300sequence.fasta&seqdb_dir=D:\SeqDB\&enzyme=Trypsin&access_method=Accession+Number&accession_num=AIA27583.1&coverage_map=0+21+33+19+52+69+18+12+24+60+14+17+28+3) | 108.43 | 12 |
|  | 1.82E+07 | 9.36E+05 | 3.17E+06 |  |  |  |  |  |  |  |
| 29 | 18 | 0 | 3 | acetate kinase | 44098.8 | 5.65 | [AIA28238.1](http://www.ncbi.nlm.nih.gov/protein/AIA28238.1) | [37](http://desktop-0t1rtqn/millbin/msdigest.cgi?missed_cleavages=2&msparams_dir=msparams_mill/&hide_protein_sequence=2&database=NCBIgb_SA300sequence.fasta&seqdb_dir=D:\SeqDB\&enzyme=Trypsin&access_method=Accession+Number&accession_num=AIA28238.1&coverage_map=0+35+16+46+34+90+34+14+15+12+11+34+15+21+23) | 100.96 | 18 |
|  | 1.53E+07 | 0.00E+00 | 2.28E+06 |  |  |  |  |  |  |  |
| 30 | 19 | 0 | 3 | thymidine phosphorylase | 46307.2 | 4.95 | [AIA28630.1](http://www.ncbi.nlm.nih.gov/protein/AIA28630.1) | [34.6](http://desktop-0t1rtqn/millbin/msdigest.cgi?missed_cleavages=2&msparams_dir=msparams_mill/&hide_protein_sequence=2&database=NCBIgb_SA300sequence.fasta&seqdb_dir=D:\SeqDB\&enzyme=Trypsin&access_method=Accession+Number&accession_num=AIA28630.1&coverage_map=0+81+27+80+35+7+31+5+19+8+15+34+23+68) | 92.96 | 19 |
|  | 2.27E+07 | 0.00E+00 | 5.70E+05 |  |  |  |  |  |  |  |
| 31 | 17 | 1 | 8 | fructose-1,6-bisphosphate aldolase | 33054 | 4.92 | [AIA29076.1](http://www.ncbi.nlm.nih.gov/protein/AIA29076.1) | [37.1](http://desktop-0t1rtqn/millbin/msdigest.cgi?missed_cleavages=2&msparams_dir=msparams_mill/&hide_protein_sequence=2&database=NCBIgb_SA300sequence.fasta&seqdb_dir=D:\SeqDB\&enzyme=Trypsin&access_method=Accession+Number&accession_num=AIA29076.1&coverage_map=0+13+15+3+23+86+23+5+15+62+17+17+17) | 92.31 | 17 |
|  | 2.38E+07 | 2.48E+05 | 5.33E+06 |  |  |  |  |  |  |  |
| 32 | 12 | 0 | 3 | DNA-directed RNA polymerase subunit beta | 135977 | 6.53 | [AKK57709.1](http://www.ncbi.nlm.nih.gov/protein/AKK57709.1) | [12](http://desktop-0t1rtqn/millbin/msdigest.cgi?missed_cleavages=2&msparams_dir=msparams_mill/&hide_protein_sequence=2&database=NCBIgb_SA300sequence.fasta&seqdb_dir=D:\SeqDB\&enzyme=Trypsin&access_method=Accession+Number&accession_num=AKK57709.1&coverage_map=0+169+20+97+15+119+14+3+26+101+24+238+20+124+16+27+11+183) | 88.51 | 12 |
|  | 1.02E+07 | 0.00E+00 | 1.14E+06 |  |  |  |  |  |  |  |
| 33 | 5 | 9 | 13 | DNA-binding protein | 9625.8 | 9.52 | [AIA27958.1](http://www.ncbi.nlm.nih.gov/protein/AIA27958.1) | [54.4](http://desktop-0t1rtqn/millbin/msdigest.cgi?missed_cleavages=2&msparams_dir=msparams_mill/&hide_protein_sequence=2&database=NCBIgb_SA300sequence.fasta&seqdb_dir=D:\SeqDB\&enzyme=Trypsin&access_method=Accession+Number&accession_num=AIA27958.1&coverage_map=1+38+3+12+37) | 83.13 | 5 |
|  | 1.17E+07 | 1.13E+07 | 3.20E+07 |  |  |  |  |  |  |  |
| 34 | 11 | 0 | 0 | ribonucleotide-diphosphate reductase subunit alpha | 80409.6 | 5.22 | [AIA27308.1](http://www.ncbi.nlm.nih.gov/protein/AIA27308.1) | [15.9](http://desktop-0t1rtqn/millbin/msdigest.cgi?missed_cleavages=2&msparams_dir=msparams_mill/&hide_protein_sequence=2&database=NCBIgb_SA300sequence.fasta&seqdb_dir=D:\SeqDB\&enzyme=Trypsin&access_method=Accession+Number&accession_num=AIA27308.1&coverage_map=0+46+10+56+23+60+11+69+13+139+26+123+29+96) | 81 | 11 |
|  | 8.97E+06 | 0.00E+00 | 0.00E+00 |  |  |  |  |  |  |  |
| 35 | 11 | 0 | 3 | hypothetical protein EX97_08030 | 35180.9 | 5.6 | [AIA28113.1](http://www.ncbi.nlm.nih.gov/protein/AIA28113.1) | [33.4](http://desktop-0t1rtqn/millbin/msdigest.cgi?missed_cleavages=2&msparams_dir=msparams_mill/&hide_protein_sequence=2&database=NCBIgb_SA300sequence.fasta&seqdb_dir=D:\SeqDB\&enzyme=Trypsin&access_method=Accession+Number&accession_num=AIA28113.1&coverage_map=0+66+22+10+49+58+22+46+17+39) | 80.38 | 11 |
|  | 3.64E+06 | 0.00E+00 | 6.39E+05 |  |  |  |  |  |  |  |
| 36 | 12 | 0 | 0 | acetolactate synthase | 61255.2 | 4.79 | [KMR42075.1](http://www.ncbi.nlm.nih.gov/protein/KMR42075.1) | [26.7](http://desktop-0t1rtqn/millbin/msdigest.cgi?missed_cleavages=2&msparams_dir=msparams_mill/&hide_protein_sequence=2&database=NCBIgb_SA300sequence.fasta&seqdb_dir=D:\SeqDB\&enzyme=Trypsin&access_method=Accession+Number&accession_num=KMR42075.1&coverage_map=0+31+19+14+41+69+26+28+17+23+45+241) | 78.71 | 12 |
|  | 1.13E+07 | 0.00E+00 | 0.00E+00 |  |  |  |  |  |  |  |
| 37 | 9 | 5 | 7 | dihydrolipoamide dehydrogenase | 49621.6 | 4.95 | [AKK58161.1](http://www.ncbi.nlm.nih.gov/protein/AKK58161.1) | [22.4](http://desktop-0t1rtqn/millbin/msdigest.cgi?missed_cleavages=2&msparams_dir=msparams_mill/&hide_protein_sequence=2&database=NCBIgb_SA300sequence.fasta&seqdb_dir=D:\SeqDB\&enzyme=Trypsin&access_method=Accession+Number&accession_num=AKK58161.1&coverage_map=0+64+23+11+10+122+11+20+28+14+21+63+12+69) | 77.6 | 9 |
|  | 3.08E+06 | 2.96E+06 | 6.45E+06 |  |  |  |  |  |  |  |
| 38 | 13 | 1 | 4 | methenyltetrahydrofolate cyclohydrolase | 30900.1 | 5.38 | [AIA27555.1](http://www.ncbi.nlm.nih.gov/protein/AIA27555.1) | [49.3](http://desktop-0t1rtqn/millbin/msdigest.cgi?missed_cleavages=2&msparams_dir=msparams_mill/&hide_protein_sequence=2&database=NCBIgb_SA300sequence.fasta&seqdb_dir=D:\SeqDB\&enzyme=Trypsin&access_method=Accession+Number&accession_num=AIA27555.1&coverage_map=0+59+43+6+68+73+30+7) | 75.91 | 13 |
|  | 3.63E+07 | 3.00E+05 | 2.69E+06 |  |  |  |  |  |  |  |
| 39 | 13 | 0 | 0 | hypothetical protein EX97_04600 | 33169.8 | 4.84 | [AIA27466.1](http://www.ncbi.nlm.nih.gov/protein/AIA27466.1) | [41](http://desktop-0t1rtqn/millbin/msdigest.cgi?missed_cleavages=2&msparams_dir=msparams_mill/&hide_protein_sequence=2&database=NCBIgb_SA300sequence.fasta&seqdb_dir=D:\SeqDB\&enzyme=Trypsin&access_method=Accession+Number&accession_num=AIA27466.1&coverage_map=0+62+38+62+20+12+29+17+36+24) | 74.46 | 13 |
|  | 2.26E+07 | 0.00E+00 | 0.00E+00 |  |  |  |  |  |  |  |
| 40 | 0 | 15 | 7 | N-acetylmuramoyl-L-alanine amidase | 69309.1 | 5.96 | [OWU45547.1](http://www.ncbi.nlm.nih.gov/protein/OWU45547.1) | [21.1](http://desktop-0t1rtqn/millbin/msdigest.cgi?missed_cleavages=2&msparams_dir=msparams_mill/&hide_protein_sequence=2&database=NCBIgb_SA300sequence.fasta&seqdb_dir=D:\SeqDB\&enzyme=Trypsin&access_method=Accession+Number&accession_num=OWU45547.1&coverage_map=0+79+18+195+26+15+24+15+28+122+19+62+16) | 73.32 | 0 |
|  | 0.00E+00 | 1.72E+07 | 5.26E+06 |  |  |  |  |  |  |  |
| 41 | 9 | 0 | 2 | DNA-directed RNA polymerase subunit beta | 133588 | 4.91 | [ALK38466.1](http://www.ncbi.nlm.nih.gov/protein/ALK38466.1) | [12](http://desktop-0t1rtqn/millbin/msdigest.cgi?missed_cleavages=2&msparams_dir=msparams_mill/&hide_protein_sequence=2&database=NCBIgb_SA300sequence.fasta&seqdb_dir=D:\SeqDB\&enzyme=Trypsin&access_method=Accession+Number&accession_num=ALK38466.1&coverage_map=0+19+16+66+39+57+21+317+12+434+30+63+25+84) | 72.91 | 9 |
|  | 8.27E+06 | 0.00E+00 | 6.40E+05 |  |  |  |  |  |  |  |
| 42 | 12 | 0 | 1 | cell division protein FtsA | 53104.9 | 4.49 | [AIA27671.1](http://www.ncbi.nlm.nih.gov/protein/AIA27671.1) | [18.9](http://desktop-0t1rtqn/millbin/msdigest.cgi?missed_cleavages=2&msparams_dir=msparams_mill/&hide_protein_sequence=2&database=NCBIgb_SA300sequence.fasta&seqdb_dir=D:\SeqDB\&enzyme=Trypsin&access_method=Accession+Number&accession_num=AIA27671.1&coverage_map=0+42+11+145+21+14+19+35+28+18+10+127) | 72.61 | 12 |
|  | 3.57E+07 | 0.00E+00 | 6.91E+05 |  |  |  |  |  |  |  |
| 43 | 9 | 0 | 2 | inorganic pyrophosphatase | 34269.4 | 4.69 | [KMR20623.1](http://www.ncbi.nlm.nih.gov/protein/KMR20623.1) | [32.3](http://desktop-0t1rtqn/millbin/msdigest.cgi?missed_cleavages=2&msparams_dir=msparams_mill/&hide_protein_sequence=2&database=NCBIgb_SA300sequence.fasta&seqdb_dir=D:\SeqDB\&enzyme=Trypsin&access_method=Accession+Number&accession_num=KMR20623.1&coverage_map=0+10+20+69+13+82+11+10+15+16+17+8+24+14) | 71.35 | 9 |
|  | 5.31E+06 | 0.00E+00 | 5.62E+05 |  |  |  |  |  |  |  |
| 44 | 1 | 6 | 10 | adhesin | 70929 | 8.79 | [AIA00463.1](http://www.ncbi.nlm.nih.gov/protein/AIA00463.1) | [18.5](http://desktop-0t1rtqn/millbin/msdigest.cgi?missed_cleavages=2&msparams_dir=msparams_mill/&hide_protein_sequence=2&database=NCBIgb_SA300sequence.fasta&seqdb_dir=D:\SeqDB\&enzyme=Trypsin&access_method=Accession+Number&accession_num=AIA00463.1&coverage_map=0+88+10+120+27+33+10+71+46+144+25+61) | 71.28 | 1 |
|  | 5.03E+06 | 3.91E+06 | 1.04E+07 |  |  |  |  |  |  |  |
| 45 | 10 | 0 | 0 | glycerol-3-phosphate dehydrogenase | 62515.9 | 6.39 | [KMS22514.1](http://www.ncbi.nlm.nih.gov/protein/KMS22514.1) | [14.3](http://desktop-0t1rtqn/millbin/msdigest.cgi?missed_cleavages=2&msparams_dir=msparams_mill/&hide_protein_sequence=2&database=NCBIgb_SA300sequence.fasta&seqdb_dir=D:\SeqDB\&enzyme=Trypsin&access_method=Accession+Number&accession_num=KMS22514.1&coverage_map=0+13+28+3+16+448+36+13) | 70.69 | 10 |
|  | 1.19E+07 | 0.00E+00 | 0.00E+00 |  |  |  |  |  |  |  |
| 46 | 0 | 12 | 0 | FKLRK protein | 56557.8 | 4.79 | [QCV67068.1](http://www.ncbi.nlm.nih.gov/protein/QCV67068.1) | [24.1](http://desktop-0t1rtqn/millbin/msdigest.cgi?missed_cleavages=2&msparams_dir=msparams_mill/&hide_protein_sequence=2&database=NCBIgb_SA300sequence.fasta&seqdb_dir=D:\SeqDB\&enzyme=Trypsin&access_method=Accession+Number&accession_num=QCV67068.1&coverage_map=0+76+28+160+8+10+19+78+19+13+27+33+20+11) | 70.54 | 0 |
|  | 0.00E+00 | 1.40E+07 | 0.00E+00 |  |  |  |  |  |  |  |
| 47 | 0 | 12 | 7 | N-acetylmuramoyl-L-alanine amidase | 35892.2 | 9.71 | [AIA27022.1](http://www.ncbi.nlm.nih.gov/protein/AIA27022.1) | [32.9](http://desktop-0t1rtqn/millbin/msdigest.cgi?missed_cleavages=2&msparams_dir=msparams_mill/&hide_protein_sequence=2&database=NCBIgb_SA300sequence.fasta&seqdb_dir=D:\SeqDB\&enzyme=Trypsin&access_method=Accession+Number&accession_num=AIA27022.1&coverage_map=0+52+55+10+14+50+17+98+24+14) | 70.29 | 0 |
|  | 0.00E+00 | 1.90E+07 | 8.94E+06 |  |  |  |  |  |  |  |
| 48 | 13 | 1 | 2 | ATP F0F1 synthase subunit alpha | 54640.1 | 4.91 | [AIA28599.1](http://www.ncbi.nlm.nih.gov/protein/AIA28599.1) | [16.7](http://desktop-0t1rtqn/millbin/msdigest.cgi?missed_cleavages=2&msparams_dir=msparams_mill/&hide_protein_sequence=2&database=NCBIgb_SA300sequence.fasta&seqdb_dir=D:\SeqDB\&enzyme=Trypsin&access_method=Accession+Number&accession_num=AIA28599.1&coverage_map=0+4+9+93+19+26+10+14+12+255+34+26) | 70.09 | 13 |
|  | 1.19E+07 | 4.21E+05 | 1.18E+06 |  |  |  |  |  |  |  |
| 49 | 2 | 0 | 9 | inosine-5-monophosphate dehydrogenase | 53021.8 | 5.54 | [KMS24688.1](http://www.ncbi.nlm.nih.gov/protein/KMS24688.1) | [30.1](http://desktop-0t1rtqn/millbin/msdigest.cgi?missed_cleavages=2&msparams_dir=msparams_mill/&hide_protein_sequence=2&database=NCBIgb_SA300sequence.fasta&seqdb_dir=D:\SeqDB\&enzyme=Trypsin&access_method=Accession+Number&accession_num=KMS24688.1&coverage_map=0+8+20+62+27+15+9+11+24+134+21+20+31+44+15+47) | 68.95 | 2 |
|  | 5.21E+05 | 0.00E+00 | 4.96E+06 |  |  |  |  |  |  |  |
| 50 | 14 | 3 | 5 | universal stress protein UspA | 18531.6 | 5.6 | [AIA28237.1](http://www.ncbi.nlm.nih.gov/protein/AIA28237.1) | [52.4](http://desktop-0t1rtqn/millbin/msdigest.cgi?missed_cleavages=2&msparams_dir=msparams_mill/&hide_protein_sequence=2&database=NCBIgb_SA300sequence.fasta&seqdb_dir=D:\SeqDB\&enzyme=Trypsin&access_method=Accession+Number&accession_num=AIA28237.1&coverage_map=0+34+25+40+62+5) | 66.03 | 14 |
|  | 1.07E+07 | 1.66E+05 | 1.16E+06 |  |  |  |  |  |  |  |
| 51 | 5 | 0 | 3 | glycyl-tRNA ligase | 53904.6 | 4.99 | [AIA28105.1](http://www.ncbi.nlm.nih.gov/protein/AIA28105.1) | [24.1](http://desktop-0t1rtqn/millbin/msdigest.cgi?missed_cleavages=2&msparams_dir=msparams_mill/&hide_protein_sequence=2&database=NCBIgb_SA300sequence.fasta&seqdb_dir=D:\SeqDB\&enzyme=Trypsin&access_method=Accession+Number&accession_num=AIA28105.1&coverage_map=0+15+27+59+26+1+13+15+28+59+18+202) | 63.07 | 5 |
|  | 5.15E+06 | 0.00E+00 | 6.86E+05 |  |  |  |  |  |  |  |
| 52 | 6 | 0 | 3 | molecular chaperone GroEL | 57629.2 | 4.55 | [AIA28527.1](http://www.ncbi.nlm.nih.gov/protein/AIA28527.1) | [24.5](http://desktop-0t1rtqn/millbin/msdigest.cgi?missed_cleavages=2&msparams_dir=msparams_mill/&hide_protein_sequence=2&database=NCBIgb_SA300sequence.fasta&seqdb_dir=D:\SeqDB\&enzyme=Trypsin&access_method=Accession+Number&accession_num=AIA28527.1&coverage_map=0+35+22+22+25+17+15+88+40+201+30+43) | 62.01 | 6 |
|  | 3.41E+06 | 0.00E+00 | 1.08E+06 |  |  |  |  |  |  |  |
| 53 | 7 | 0 | 1 | 30S ribosomal protein S2 | 29150.9 | 5.44 | [AIA27742.1](http://www.ncbi.nlm.nih.gov/protein/AIA27742.1) | [34.1](http://desktop-0t1rtqn/millbin/msdigest.cgi?missed_cleavages=2&msparams_dir=msparams_mill/&hide_protein_sequence=2&database=NCBIgb_SA300sequence.fasta&seqdb_dir=D:\SeqDB\&enzyme=Trypsin&access_method=Accession+Number&accession_num=AIA27742.1&coverage_map=0+35+10+13+16+39+18+24+12+10+31+47) | 60.81 | 7 |
|  | 6.89E+06 | 0.00E+00 | 7.41E+05 |  |  |  |  |  |  |  |
| 54 | 6 | 0 | 7 | 50S ribosomal protein L2 | 30211.5 | 10.85 | [AIA28734.1](http://www.ncbi.nlm.nih.gov/protein/AIA28734.1) | [31.4](http://desktop-0t1rtqn/millbin/msdigest.cgi?missed_cleavages=2&msparams_dir=msparams_mill/&hide_protein_sequence=2&database=NCBIgb_SA300sequence.fasta&seqdb_dir=D:\SeqDB\&enzyme=Trypsin&access_method=Accession+Number&accession_num=AIA28734.1&coverage_map=0+14+12+61+15+6+42+39+18+70) | 60.63 | 6 |
|  | 2.64E+06 | 0.00E+00 | 3.14E+06 |  |  |  |  |  |  |  |
| 55 | 7 | 0 | 1 | carbamoyl phosphate synthase large subunit | 117670 | 4.87 | [KMR30550.1](http://www.ncbi.nlm.nih.gov/protein/KMR30550.1) | [11.2](http://desktop-0t1rtqn/millbin/msdigest.cgi?missed_cleavages=2&msparams_dir=msparams_mill/&hide_protein_sequence=2&database=NCBIgb_SA300sequence.fasta&seqdb_dir=D:\SeqDB\&enzyme=Trypsin&access_method=Accession+Number&accession_num=KMR30550.1&coverage_map=0+133+24+167+32+419+31+217+32+2) | 59.98 | 7 |
|  | 5.65E+06 | 0.00E+00 | 7.67E+04 |  |  |  |  |  |  |  |
| 56 | 9 | 0 | 0 | succinyl-CoA synthetase subunit beta | 42325.8 | 4.91 | [KMS26052.1](http://www.ncbi.nlm.nih.gov/protein/KMS26052.1) | [19](http://desktop-0t1rtqn/millbin/msdigest.cgi?missed_cleavages=2&msparams_dir=msparams_mill/&hide_protein_sequence=2&database=NCBIgb_SA300sequence.fasta&seqdb_dir=D:\SeqDB\&enzyme=Trypsin&access_method=Accession+Number&accession_num=KMS26052.1&coverage_map=0+23+11+61+21+99+10+78+21+13+11+40) | 59.69 | 9 |
|  | 1.54E+06 | 0.00E+00 | 0.00E+00 |  |  |  |  |  |  |  |
| 57 | 4 | 0 | 1 | Clp protease ClpX | 77965 | 4.88 | [KMR77579.1](http://www.ncbi.nlm.nih.gov/protein/KMR77579.1) | [14.8](http://desktop-0t1rtqn/millbin/msdigest.cgi?missed_cleavages=2&msparams_dir=msparams_mill/&hide_protein_sequence=2&database=NCBIgb_SA300sequence.fasta&seqdb_dir=D:\SeqDB\&enzyme=Trypsin&access_method=Accession+Number&accession_num=KMR77579.1&coverage_map=0+93+10+84+28+225+23+71+29+122+14+2) | 59.45 | 4 |
|  | 5.50E+05 | 0.00E+00 | 5.62E+05 |  |  |  |  |  |  |  |
| 58 | 7 | 0 | 0 | polynucleotide phosphorylase | 77504.8 | 4.89 | [KMS43492.1](http://www.ncbi.nlm.nih.gov/protein/KMS43492.1) | [12.8](http://desktop-0t1rtqn/millbin/msdigest.cgi?missed_cleavages=2&msparams_dir=msparams_mill/&hide_protein_sequence=2&database=NCBIgb_SA300sequence.fasta&seqdb_dir=D:\SeqDB\&enzyme=Trypsin&access_method=Accession+Number&accession_num=KMS43492.1&coverage_map=0+35+17+215+39+169+23+22+11+167) | 57.8 | 7 |
|  | 7.42E+06 | 0.00E+00 | 0.00E+00 |  |  |  |  |  |  |  |
| 59 | 8 | 0 | 0 | ATP-dependent protease | 46581.9 | 4.54 | [AIA28201.1](http://www.ncbi.nlm.nih.gov/protein/AIA28201.1) | [28](http://desktop-0t1rtqn/millbin/msdigest.cgi?missed_cleavages=2&msparams_dir=msparams_mill/&hide_protein_sequence=2&database=NCBIgb_SA300sequence.fasta&seqdb_dir=D:\SeqDB\&enzyme=Trypsin&access_method=Accession+Number&accession_num=AIA28201.1&coverage_map=0+132+30+101+38+32+22+34+28+3) | 57.47 | 8 |
|  | 1.01E+07 | 0.00E+00 | 0.00E+00 |  |  |  |  |  |  |  |
| 60 | 8 | 0 | 0 | 50S ribosomal protein L5 | 20266.3 | 9.32 | [AIA28725.1](http://www.ncbi.nlm.nih.gov/protein/AIA28725.1) | [41.8](http://desktop-0t1rtqn/millbin/msdigest.cgi?missed_cleavages=2&msparams_dir=msparams_mill/&hide_protein_sequence=2&database=NCBIgb_SA300sequence.fasta&seqdb_dir=D:\SeqDB\&enzyme=Trypsin&access_method=Accession+Number&accession_num=AIA28725.1&coverage_map=0+7+11+15+36+81+28+1) | 57.44 | 8 |
|  | 6.45E+06 | 0.00E+00 | 0.00E+00 |  |  |  |  |  |  |  |
| 61 | 5 | 0 | 0 | NADH dehydrogenase | 55006.1 | 4.66 | [ALK35704.1](http://www.ncbi.nlm.nih.gov/protein/ALK35704.1) | [25.2](http://desktop-0t1rtqn/millbin/msdigest.cgi?missed_cleavages=2&msparams_dir=msparams_mill/&hide_protein_sequence=2&database=NCBIgb_SA300sequence.fasta&seqdb_dir=D:\SeqDB\&enzyme=Trypsin&access_method=Accession+Number&accession_num=ALK35704.1&coverage_map=0+59+39+92+36+177+53+51) | 56.81 | 5 |
|  | 3.86E+05 | 0.00E+00 | 0.00E+00 |  |  |  |  |  |  |  |
| 62 | 3 | 1 | 0 | NADH dehydrogenase | 54965 | 4.68 | [AHZ98296.1](http://www.ncbi.nlm.nih.gov/protein/AHZ98296.1) | [20.1](http://desktop-0t1rtqn/millbin/msdigest.cgi?missed_cleavages=2&msparams_dir=msparams_mill/&hide_protein_sequence=2&database=NCBIgb_SA300sequence.fasta&seqdb_dir=D:\SeqDB\&enzyme=Trypsin&access_method=Accession+Number&accession_num=AHZ98296.1&coverage_map=0+190+36+4+52+121+14+90) | 32.46 | 3 |
|  | 2.21E+05 | 1.25E+07 | 0.00E+00 |  |  |  |  |  |  |  |
| 63 | 3 | 0 | 6 | transketolase | 72293.5 | 4.94 | [OWU45249.1](http://www.ncbi.nlm.nih.gov/protein/OWU45249.1) | [15.1](http://desktop-0t1rtqn/millbin/msdigest.cgi?missed_cleavages=2&msparams_dir=msparams_mill/&hide_protein_sequence=2&database=NCBIgb_SA300sequence.fasta&seqdb_dir=D:\SeqDB\&enzyme=Trypsin&access_method=Accession+Number&accession_num=OWU45249.1&coverage_map=0+105+32+68+12+67+17+198+20+109+19+15) | 56.53 | 3 |
|  | 1.14E+06 | 0.00E+00 | 2.57E+06 |  |  |  |  |  |  |  |
| 64 | 7 | 0 | 0 | UDP-N-acetylglucosamine 1-carboxyvinyltransferase | 45359.4 | 5.49 | [AIA28618.1](http://www.ncbi.nlm.nih.gov/protein/AIA28618.1) | [23.1](http://desktop-0t1rtqn/millbin/msdigest.cgi?missed_cleavages=2&msparams_dir=msparams_mill/&hide_protein_sequence=2&database=NCBIgb_SA300sequence.fasta&seqdb_dir=D:\SeqDB\&enzyme=Trypsin&access_method=Accession+Number&accession_num=AIA28618.1&coverage_map=0+53+12+125+19+88+36+9+30+47) | 56.22 | 7 |
|  | 5.20E+06 | 0.00E+00 | 0.00E+00 |  |  |  |  |  |  |  |
| 65 | 6 | 1 | 0 | 30S ribosomal protein S7 | 17794.2 | 9.98 | [AIA27103.1](http://www.ncbi.nlm.nih.gov/protein/AIA27103.1) | [33.3](http://desktop-0t1rtqn/millbin/msdigest.cgi?missed_cleavages=2&msparams_dir=msparams_mill/&hide_protein_sequence=2&database=NCBIgb_SA300sequence.fasta&seqdb_dir=D:\SeqDB\&enzyme=Trypsin&access_method=Accession+Number&accession_num=AIA27103.1&coverage_map=0+41+35+43+17+20) | 55.99 | 6 |
|  | 9.41E+06 | 3.37E+04 | 0.00E+00 |  |  |  |  |  |  |  |
| 66 | 0 | 8 | 4 | MAP domain-containing protein | 77083.1 | 9.85 | [QCV68621.1](http://www.ncbi.nlm.nih.gov/protein/QCV68621.1) | [10.9](http://desktop-0t1rtqn/millbin/msdigest.cgi?missed_cleavages=2&msparams_dir=msparams_mill/&hide_protein_sequence=2&database=NCBIgb_SA300sequence.fasta&seqdb_dir=D:\SeqDB\&enzyme=Trypsin&access_method=Accession+Number&accession_num=QCV68621.1&coverage_map=0+48+24+51+15+96+15+193+21+221) | 54.4 | 0 |
|  | 0.00E+00 | 5.02E+06 | 1.27E+06 |  |  |  |  |  |  |  |
| 67 | 7 | 1 | 7 | 50S ribosomal protein L1 | 24707.8 | 9 | [AIA27095.1](http://www.ncbi.nlm.nih.gov/protein/AIA27095.1) | [34.7](http://desktop-0t1rtqn/millbin/msdigest.cgi?missed_cleavages=2&msparams_dir=msparams_mill/&hide_protein_sequence=2&database=NCBIgb_SA300sequence.fasta&seqdb_dir=D:\SeqDB\&enzyme=Trypsin&access_method=Accession+Number&accession_num=AIA27095.1&coverage_map=0+80+46+51+21+12+13+7) | 54.24 | 7 |
|  | 2.00E+07 | 3.02E+04 | 4.96E+06 |  |  |  |  |  |  |  |
| 68 | 8 | 0 | 5 | 50S ribosomal protein L10 | 17709.8 | 4.82 | [AIA27096.1](http://www.ncbi.nlm.nih.gov/protein/AIA27096.1) | [45.7](http://desktop-0t1rtqn/millbin/msdigest.cgi?missed_cleavages=2&msparams_dir=msparams_mill/&hide_protein_sequence=2&database=NCBIgb_SA300sequence.fasta&seqdb_dir=D:\SeqDB\&enzyme=Trypsin&access_method=Accession+Number&accession_num=AIA27096.1&coverage_map=0+9+33+24+28+15+15+42) | 52.84 | 8 |
|  | 4.07E+06 | 0.00E+00 | 1.06E+05 |  |  |  |  |  |  |  |
| 69 | 5 | 2 | 1 | 30S ribosomal protein S9 | 14829.7 | 10.56 | [AIA28704.1](http://www.ncbi.nlm.nih.gov/protein/AIA28704.1) | [42.4](http://desktop-0t1rtqn/millbin/msdigest.cgi?missed_cleavages=2&msparams_dir=msparams_mill/&hide_protein_sequence=2&database=NCBIgb_SA300sequence.fasta&seqdb_dir=D:\SeqDB\&enzyme=Trypsin&access_method=Accession+Number&accession_num=AIA28704.1&coverage_map=0+22+13+3+22+27+10+5+11+19) | 51.94 | 5 |
|  | 3.70E+06 | 1.06E+06 | 2.22E+04 |  |  |  |  |  |  |  |
| 70 | 2 | 0 | 7 | triosephosphate isomerase | 27432.4 | 4.8 | [AIA27352.1](http://www.ncbi.nlm.nih.gov/protein/AIA27352.1) | [35.9](http://desktop-0t1rtqn/millbin/msdigest.cgi?missed_cleavages=2&msparams_dir=msparams_mill/&hide_protein_sequence=2&database=NCBIgb_SA300sequence.fasta&seqdb_dir=D:\SeqDB\&enzyme=Trypsin&access_method=Accession+Number&accession_num=AIA27352.1&coverage_map=0+20+34+3+34+60+11+79+12) | 51.87 | 2 |
|  | 8.51E+04 | 0.00E+00 | 5.69E+06 |  |  |  |  |  |  |  |
| 71 | 9 | 0 | 2 | ribose-phosphate pyrophosphokinase | 35625.5 | 5.88 | [AIA27057.1](http://www.ncbi.nlm.nih.gov/protein/AIA27057.1) | [26.7](http://desktop-0t1rtqn/millbin/msdigest.cgi?missed_cleavages=2&msparams_dir=msparams_mill/&hide_protein_sequence=2&database=NCBIgb_SA300sequence.fasta&seqdb_dir=D:\SeqDB\&enzyme=Trypsin&access_method=Accession+Number&accession_num=AIA27057.1&coverage_map=0+17+19+94+30+62+23+50+14+12) | 51.12 | 9 |
|  | 1.97E+07 | 0.00E+00 | 1.89E+05 |  |  |  |  |  |  |  |
| 72 | 6 | 0 | 1 | aldehyde dehydrogenase | 52072.9 | 5.08 | [KMS32361.1](http://www.ncbi.nlm.nih.gov/protein/KMS32361.1) | [17.8](http://desktop-0t1rtqn/millbin/msdigest.cgi?missed_cleavages=2&msparams_dir=msparams_mill/&hide_protein_sequence=2&database=NCBIgb_SA300sequence.fasta&seqdb_dir=D:\SeqDB\&enzyme=Trypsin&access_method=Accession+Number&accession_num=KMS32361.1&coverage_map=0+41+18+21+21+55+32+266+14+7) | 49.42 | 6 |
|  | 2.87E+06 | 0.00E+00 | 4.89E+05 |  |  |  |  |  |  |  |
| 73 | 6 | 0 | 0 | PTS glucose transporter subunit IIA | 17960.2 | 4.52 | [AIA27911.1](http://www.ncbi.nlm.nih.gov/protein/AIA27911.1) | [35.5](http://desktop-0t1rtqn/millbin/msdigest.cgi?missed_cleavages=2&msparams_dir=msparams_mill/&hide_protein_sequence=2&database=NCBIgb_SA300sequence.fasta&seqdb_dir=D:\SeqDB\&enzyme=Trypsin&access_method=Accession+Number&accession_num=AIA27911.1&coverage_map=0+14+48+55+11+38) | 48.95 | 6 |
|  | 3.85E+06 | 0.00E+00 | 0.00E+00 |  |  |  |  |  |  |  |
| 74 | 1 | 5 | 2 | delta-hemolysin | 2978.5 | 8.19 | [KMR09253.1](http://www.ncbi.nlm.nih.gov/protein/KMR09253.1) | [92.3](http://desktop-0t1rtqn/millbin/msdigest.cgi?missed_cleavages=2&msparams_dir=msparams_mill/&hide_protein_sequence=2&database=NCBIgb_SA300sequence.fasta&seqdb_dir=D:\SeqDB\&enzyme=Trypsin&access_method=Accession+Number&accession_num=KMR09253.1&coverage_map=1+25+1) | 48.67 | 1 |
|  | 4.65E+05 | 3.02E+07 | 5.62E+05 |  |  |  |  |  |  |  |
| 75 | 6 | 0 | 0 | 6-phosphofructokinase | 35124.3 | 5.63 | [AIA28226.1](http://www.ncbi.nlm.nih.gov/protein/AIA28226.1) | [27.3](http://desktop-0t1rtqn/millbin/msdigest.cgi?missed_cleavages=2&msparams_dir=msparams_mill/&hide_protein_sequence=2&database=NCBIgb_SA300sequence.fasta&seqdb_dir=D:\SeqDB\&enzyme=Trypsin&access_method=Accession+Number&accession_num=AIA28226.1&coverage_map=0+92+15+10+37+10+9+61+11+23+16+38) | 48.5 | 6 |
|  | 4.22E+06 | 0.00E+00 | 0.00E+00 |  |  |  |  |  |  |  |
| 76 | 0 | 2 | 7 | glutamine synthetase | 51139.1 | 5.08 | [AKK58369.1](http://www.ncbi.nlm.nih.gov/protein/AKK58369.1) | [20.1](http://desktop-0t1rtqn/millbin/msdigest.cgi?missed_cleavages=2&msparams_dir=msparams_mill/&hide_protein_sequence=2&database=NCBIgb_SA300sequence.fasta&seqdb_dir=D:\SeqDB\&enzyme=Trypsin&access_method=Accession+Number&accession_num=AKK58369.1&coverage_map=0+119+22+113+28+18+16+21+24+85) | 48.18 | 0 |
|  | 0.00E+00 | 1.17E+06 | 6.11E+06 |  |  |  |  |  |  |  |
| 77 | 8 | 0 | 5 | 50S ribosomal protein L6 | 19786.2 | 9.54 | [AIA28722.1](http://www.ncbi.nlm.nih.gov/protein/AIA28722.1) | [35.3](http://desktop-0t1rtqn/millbin/msdigest.cgi?missed_cleavages=2&msparams_dir=msparams_mill/&hide_protein_sequence=2&database=NCBIgb_SA300sequence.fasta&seqdb_dir=D:\SeqDB\&enzyme=Trypsin&access_method=Accession+Number&accession_num=AIA28722.1&coverage_map=0+69+26+6+26+11+11+29) | 48.14 | 8 |
|  | 2.20E+06 | 0.00E+00 | 3.17E+05 |  |  |  |  |  |  |  |
| 78 | 6 | 0 | 0 | alcohol dehydrogenase | 36511.1 | 5.27 | [AIA28680.1](http://www.ncbi.nlm.nih.gov/protein/AIA28680.1) | [29.6](http://desktop-0t1rtqn/millbin/msdigest.cgi?missed_cleavages=2&msparams_dir=msparams_mill/&hide_protein_sequence=2&database=NCBIgb_SA300sequence.fasta&seqdb_dir=D:\SeqDB\&enzyme=Trypsin&access_method=Accession+Number&accession_num=AIA28680.1&coverage_map=0+77+10+24+56+32+33+102) | 48.11 | 6 |
|  | 2.60E+06 | 0.00E+00 | 0.00E+00 |  |  |  |  |  |  |  |
| 79 | 7 | 0 | 0 | chromosome partitioning protein ParA | 38361.6 | 5.29 | [OWU43255.1](http://www.ncbi.nlm.nih.gov/protein/OWU43255.1) | [17.2](http://desktop-0t1rtqn/millbin/msdigest.cgi?missed_cleavages=2&msparams_dir=msparams_mill/&hide_protein_sequence=2&database=NCBIgb_SA300sequence.fasta&seqdb_dir=D:\SeqDB\&enzyme=Trypsin&access_method=Accession+Number&accession_num=OWU43255.1&coverage_map=0+98+21+61+17+104+23+30) | 47.72 | 7 |
|  | 4.75E+06 | 0.00E+00 | 0.00E+00 |  |  |  |  |  |  |  |
| 80 | 10 | 2 | 5 | alkaline shock protein 23 | 19191 | 5.13 | [AIA28675.1](http://www.ncbi.nlm.nih.gov/protein/AIA28675.1) | [41.4](http://desktop-0t1rtqn/millbin/msdigest.cgi?missed_cleavages=2&msparams_dir=msparams_mill/&hide_protein_sequence=2&database=NCBIgb_SA300sequence.fasta&seqdb_dir=D:\SeqDB\&enzyme=Trypsin&access_method=Accession+Number&accession_num=AIA28675.1&coverage_map=0+41+10+16+28+7+11+14+21+21) | 47.65 | 10 |
|  | 8.80E+06 | 6.56E+05 | 4.48E+06 |  |  |  |  |  |  |  |
| 81 | 8 | 1 | 2 | uracil phosphoribosyltransferase | 23106.5 | 6.08 | [AIA28606.1](http://www.ncbi.nlm.nih.gov/protein/AIA28606.1) | [33](http://desktop-0t1rtqn/millbin/msdigest.cgi?missed_cleavages=2&msparams_dir=msparams_mill/&hide_protein_sequence=2&database=NCBIgb_SA300sequence.fasta&seqdb_dir=D:\SeqDB\&enzyme=Trypsin&access_method=Accession+Number&accession_num=AIA28606.1&coverage_map=0+30+16+33+17+29+23+8+13+40) | 46.65 | 8 |
|  | 1.67E+07 | 5.83E+04 | 8.07E+05 |  |  |  |  |  |  |  |
| 82 | 7 | 0 | 4 | 30S ribosomal protein S9 | 23012.9 | 10.02 | [AIA28246.1](http://www.ncbi.nlm.nih.gov/protein/AIA28246.1) | [34](http://desktop-0t1rtqn/millbin/msdigest.cgi?missed_cleavages=2&msparams_dir=msparams_mill/&hide_protein_sequence=2&database=NCBIgb_SA300sequence.fasta&seqdb_dir=D:\SeqDB\&enzyme=Trypsin&access_method=Accession+Number&accession_num=AIA28246.1&coverage_map=0+14+10+19+9+95+31+4+18) | 45.17 | 7 |
|  | 8.97E+06 | 0.00E+00 | 1.39E+06 |  |  |  |  |  |  |  |
| 83 | 5 | 0 | 0 | glyoxal reductase | 32530.4 | 5.16 | [AIA27281.1](http://www.ncbi.nlm.nih.gov/protein/AIA27281.1) | [26.1](http://desktop-0t1rtqn/millbin/msdigest.cgi?missed_cleavages=2&msparams_dir=msparams_mill/&hide_protein_sequence=2&database=NCBIgb_SA300sequence.fasta&seqdb_dir=D:\SeqDB\&enzyme=Trypsin&access_method=Accession+Number&accession_num=AIA27281.1&coverage_map=1+23+8+11+50+30+72+10+75) | 44.21 | 5 |
|  | 4.83E+05 | 0.00E+00 | 0.00E+00 |  |  |  |  |  |  |  |
| 84 | 6 | 1 | 2 | 3-ketoacyl-ACP reductase | 25943.2 | 5.33 | [AIA27717.1](http://www.ncbi.nlm.nih.gov/protein/AIA27717.1) | [31.5](http://desktop-0t1rtqn/millbin/msdigest.cgi?missed_cleavages=2&msparams_dir=msparams_mill/&hide_protein_sequence=2&database=NCBIgb_SA300sequence.fasta&seqdb_dir=D:\SeqDB\&enzyme=Trypsin&access_method=Accession+Number&accession_num=AIA27717.1&coverage_map=0+16+21+35+20+81+36+35) | 44 | 6 |
|  | 5.28E+06 | 2.81E+05 | 1.04E+06 |  |  |  |  |  |  |  |
| 85 | 5 | 0 | 0 | D-alanine--D-alanine ligase | 40415.1 | 4.95 | [QEA19612.1](http://www.ncbi.nlm.nih.gov/protein/QEA19612.1) | [16.8](http://desktop-0t1rtqn/millbin/msdigest.cgi?missed_cleavages=2&msparams_dir=msparams_mill/&hide_protein_sequence=2&database=NCBIgb_SA300sequence.fasta&seqdb_dir=D:\SeqDB\&enzyme=Trypsin&access_method=Accession+Number&accession_num=QEA19612.1&coverage_map=0+60+20+139+22+15+18+82) | 43.74 | 5 |
|  | 3.45E+06 | 0.00E+00 | 0.00E+00 |  |  |  |  |  |  |  |
| 86 | 7 | 0 | 0 | aminoglycoside phosphotransferase | 31415 | 4.57 | [KMS09981.1](http://www.ncbi.nlm.nih.gov/protein/KMS09981.1) | [31.4](http://desktop-0t1rtqn/millbin/msdigest.cgi?missed_cleavages=2&msparams_dir=msparams_mill/&hide_protein_sequence=2&database=NCBIgb_SA300sequence.fasta&seqdb_dir=D:\SeqDB\&enzyme=Trypsin&access_method=Accession+Number&accession_num=KMS09981.1&coverage_map=0+110+59+60+24+11) | 43.62 | 7 |
|  | 4.24E+06 | 0.00E+00 | 0.00E+00 |  |  |  |  |  |  |  |
| 87 | 7 | 0 | 0 | alanine dehydrogenase | 40348.3 | 5.15 | [AIA27926.1](http://www.ncbi.nlm.nih.gov/protein/AIA27926.1) | [14.7](http://desktop-0t1rtqn/millbin/msdigest.cgi?missed_cleavages=2&msparams_dir=msparams_mill/&hide_protein_sequence=2&database=NCBIgb_SA300sequence.fasta&seqdb_dir=D:\SeqDB\&enzyme=Trypsin&access_method=Accession+Number&accession_num=AIA27926.1&coverage_map=0+56+15+33+16+3+14+56+10+169) | 43.47 | 7 |
|  | 8.75E+05 | 0.00E+00 | 0.00E+00 |  |  |  |  |  |  |  |
| 88 | 4 | 2 | 3 | branched-chain alpha-keto acid dehydrogenase subunit E2 | 46438.6 | 4.9 | [AIA27585.1](http://www.ncbi.nlm.nih.gov/protein/AIA27585.1) | [12.7](http://desktop-0t1rtqn/millbin/msdigest.cgi?missed_cleavages=2&msparams_dir=msparams_mill/&hide_protein_sequence=2&database=NCBIgb_SA300sequence.fasta&seqdb_dir=D:\SeqDB\&enzyme=Trypsin&access_method=Accession+Number&accession_num=AIA27585.1&coverage_map=0+220+21+74+15+53+19+28) | 43.15 | 4 |
|  | 2.42E+06 | 8.55E+05 | 1.10E+06 |  |  |  |  |  |  |  |
| 89 | 0 | 4 | 5 | hypothetical protein EX97_03135 | 18651 | 9.24 | [AIA27178.1](http://www.ncbi.nlm.nih.gov/protein/AIA27178.1) | [31.5](http://desktop-0t1rtqn/millbin/msdigest.cgi?missed_cleavages=2&msparams_dir=msparams_mill/&hide_protein_sequence=2&database=NCBIgb_SA300sequence.fasta&seqdb_dir=D:\SeqDB\&enzyme=Trypsin&access_method=Accession+Number&accession_num=AIA27178.1&coverage_map=0+37+13+49+20+14+10+2+10+13) | 41.99 | 0 |
|  | 0.00E+00 | 1.02E+06 | 1.03E+06 |  |  |  |  |  |  |  |
| 90 | 5 | 0 | 3 | ethanol-active dehydrogenase/acetaldehyde-active reductase | 36446.3 | 5.35 | [AIA27170.1](http://www.ncbi.nlm.nih.gov/protein/AIA27170.1) | [17.2](http://desktop-0t1rtqn/millbin/msdigest.cgi?missed_cleavages=2&msparams_dir=msparams_mill/&hide_protein_sequence=2&database=NCBIgb_SA300sequence.fasta&seqdb_dir=D:\SeqDB\&enzyme=Trypsin&access_method=Accession+Number&accession_num=AIA27170.1&coverage_map=0+229+12+15+10+7+16+22+20+5) | 41.75 | 5 |
|  | 1.62E+06 | 0.00E+00 | 1.58E+06 |  |  |  |  |  |  |  |
| 91 | 8 | 0 | 0 | threonine dehydratase | 37389.3 | 6.38 | [KMR78773.1](http://www.ncbi.nlm.nih.gov/protein/KMR78773.1) | [19.3](http://desktop-0t1rtqn/millbin/msdigest.cgi?missed_cleavages=2&msparams_dir=msparams_mill/&hide_protein_sequence=2&database=NCBIgb_SA300sequence.fasta&seqdb_dir=D:\SeqDB\&enzyme=Trypsin&access_method=Accession+Number&accession_num=KMR78773.1&coverage_map=0+101+18+165+18+9+31+4) | 41.46 | 8 |
|  | 3.84E+06 | 0.00E+00 | 0.00E+00 |  |  |  |  |  |  |  |
| 92 | 4 | 0 | 1 | phosphoglycerate mutase (2,3-diphosphoglycerate-independent) | 56510.1 | 4.74 | [OWU54794.1](http://www.ncbi.nlm.nih.gov/protein/OWU54794.1) | [18.2](http://desktop-0t1rtqn/millbin/msdigest.cgi?missed_cleavages=2&msparams_dir=msparams_mill/&hide_protein_sequence=2&database=NCBIgb_SA300sequence.fasta&seqdb_dir=D:\SeqDB\&enzyme=Trypsin&access_method=Accession+Number&accession_num=OWU54794.1&coverage_map=0+291+13+70+51+52+28) | 40.82 | 4 |
|  | 4.55E+06 | 0.00E+00 | 1.18E+06 |  |  |  |  |  |  |  |
| 93 | 8 | 0 | 0 | 30S ribosomal protein S10 | 11576.3 | 9.74 | [AIA28738.1](http://www.ncbi.nlm.nih.gov/protein/AIA28738.1) | [44.1](http://desktop-0t1rtqn/millbin/msdigest.cgi?missed_cleavages=2&msparams_dir=msparams_mill/&hide_protein_sequence=2&database=NCBIgb_SA300sequence.fasta&seqdb_dir=D:\SeqDB\&enzyme=Trypsin&access_method=Accession+Number&accession_num=AIA28738.1&coverage_map=0+31+15+26+30) | 40.72 | 8 |
|  | 1.28E+07 | 0.00E+00 | 0.00E+00 |  |  |  |  |  |  |  |
| 94 | 2 | 2 | 5 | 50S ribosomal protein L4 | 22464.3 | 9.9 | [AIA28736.1](http://www.ncbi.nlm.nih.gov/protein/AIA28736.1) | [33.8](http://desktop-0t1rtqn/millbin/msdigest.cgi?missed_cleavages=2&msparams_dir=msparams_mill/&hide_protein_sequence=2&database=NCBIgb_SA300sequence.fasta&seqdb_dir=D:\SeqDB\&enzyme=Trypsin&access_method=Accession+Number&accession_num=AIA28736.1&coverage_map=0+13+28+99+10+18+32+7) | 40.64 | 2 |
|  | 3.22E+06 | 7.68E+05 | 5.85E+06 |  |  |  |  |  |  |  |
| 95 | 6 | 0 | 0 | lysyl-tRNA synthetase | 56732.2 | 5.11 | [ALK35810.1](http://www.ncbi.nlm.nih.gov/protein/ALK35810.1) | [18.3](http://desktop-0t1rtqn/millbin/msdigest.cgi?missed_cleavages=2&msparams_dir=msparams_mill/&hide_protein_sequence=2&database=NCBIgb_SA300sequence.fasta&seqdb_dir=D:\SeqDB\&enzyme=Trypsin&access_method=Accession+Number&accession_num=ALK35810.1&coverage_map=0+106+16+140+29+35+11+21+35+102) | 40.62 | 6 |
|  | 5.33E+06 | 0.00E+00 | 0.00E+00 |  |  |  |  |  |  |  |
| 96 | 8 | 0 | 1 | isocitrate dehydrogenase | 46508.9 | 4.84 | [KMS49865.1](http://www.ncbi.nlm.nih.gov/protein/KMS49865.1) | [18.4](http://desktop-0t1rtqn/millbin/msdigest.cgi?missed_cleavages=2&msparams_dir=msparams_mill/&hide_protein_sequence=2&database=NCBIgb_SA300sequence.fasta&seqdb_dir=D:\SeqDB\&enzyme=Trypsin&access_method=Accession+Number&accession_num=KMS49865.1&coverage_map=0+5+30+30+18+149+30+160) | 40.59 | 8 |
|  | 2.59E+07 | 0.00E+00 | 8.34E+05 |  |  |  |  |  |  |  |
| 97 | 4 | 1 | 1 | DNA gyrase subunit A | 99441.4 | 5.06 | [QCV66696.1](http://www.ncbi.nlm.nih.gov/protein/QCV66696.1) | [6.2](http://desktop-0t1rtqn/millbin/msdigest.cgi?missed_cleavages=2&msparams_dir=msparams_mill/&hide_protein_sequence=2&database=NCBIgb_SA300sequence.fasta&seqdb_dir=D:\SeqDB\&enzyme=Trypsin&access_method=Accession+Number&accession_num=QCV66696.1&coverage_map=0+19+28+153+27+660) | 38.77 | 4 |
|  | 1.61E+06 | 1.26E+03 | 6.04E+04 |  |  |  |  |  |  |  |
| 98 | 4 | 0 | 2 | NADH dehydrogenase | 44388.8 | 5.36 | [AIA27438.1](http://www.ncbi.nlm.nih.gov/protein/AIA27438.1) | [17.4](http://desktop-0t1rtqn/millbin/msdigest.cgi?missed_cleavages=2&msparams_dir=msparams_mill/&hide_protein_sequence=2&database=NCBIgb_SA300sequence.fasta&seqdb_dir=D:\SeqDB\&enzyme=Trypsin&access_method=Accession+Number&accession_num=AIA27438.1&coverage_map=0+26+14+165+20+16+25+3+11+122) | 38.72 | 4 |
|  | 4.90E+06 | 0.00E+00 | 1.43E+05 |  |  |  |  |  |  |  |
| 99 | 9 | 0 | 0 | glycerol-3-phosphate dehydrogenase | 37149.7 | 5.95 | [AIA28213.1](http://www.ncbi.nlm.nih.gov/protein/AIA28213.1) | [17](http://desktop-0t1rtqn/millbin/msdigest.cgi?missed_cleavages=2&msparams_dir=msparams_mill/&hide_protein_sequence=2&database=NCBIgb_SA300sequence.fasta&seqdb_dir=D:\SeqDB\&enzyme=Trypsin&access_method=Accession+Number&accession_num=AIA28213.1&coverage_map=0+133+30+75+13+69+15+6) | 38.44 | 9 |
|  | 2.64E+07 | 0.00E+00 | 0.00E+00 |  |  |  |  |  |  |  |
| 100 | 4 | 0 | 0 | lactoylglutathione lyase | 30199.2 | 4.32 | [KMR82918.1](http://www.ncbi.nlm.nih.gov/protein/KMR82918.1) | [28.6](http://desktop-0t1rtqn/millbin/msdigest.cgi?missed_cleavages=2&msparams_dir=msparams_mill/&hide_protein_sequence=2&database=NCBIgb_SA300sequence.fasta&seqdb_dir=D:\SeqDB\&enzyme=Trypsin&access_method=Accession+Number&accession_num=KMR82918.1&coverage_map=0+18+20+11+22+117+35+46) | 37.76 | 4 |
|  | 2.70E+06 | 0.00E+00 | 0.00E+00 |  |  |  |  |  |  |  |
| 101 | 6 | 0 | 0 | citrate synthase | 42706.7 | 5.41 | [AIA28221.1](http://www.ncbi.nlm.nih.gov/protein/AIA28221.1) | [24.6](http://desktop-0t1rtqn/millbin/msdigest.cgi?missed_cleavages=2&msparams_dir=msparams_mill/&hide_protein_sequence=2&database=NCBIgb_SA300sequence.fasta&seqdb_dir=D:\SeqDB\&enzyme=Trypsin&access_method=Accession+Number&accession_num=AIA28221.1&coverage_map=0+6+47+109+14+39+31+127) | 37.71 | 6 |
|  | 4.80E+06 | 0.00E+00 | 0.00E+00 |  |  |  |  |  |  |  |
| 102 | 5 | 0 | 0 | dihydroxynaphthoic acid synthetase | 30653.3 | 5.41 | [AIA27538.1](http://www.ncbi.nlm.nih.gov/protein/AIA27538.1) | [26.7](http://desktop-0t1rtqn/millbin/msdigest.cgi?missed_cleavages=2&msparams_dir=msparams_mill/&hide_protein_sequence=2&database=NCBIgb_SA300sequence.fasta&seqdb_dir=D:\SeqDB\&enzyme=Trypsin&access_method=Accession+Number&accession_num=AIA27538.1&coverage_map=0+40+11+64+31+75+31+21) | 37.68 | 5 |
|  | 1.41E+07 | 0.00E+00 | 0.00E+00 |  |  |  |  |  |  |  |
| 103 | 3 | 0 | 0 | succinyl-CoA synthetase subsunit alpha | 31769.9 | 5.47 | [AIA27732.1](http://www.ncbi.nlm.nih.gov/protein/AIA27732.1) | [20.1](http://desktop-0t1rtqn/millbin/msdigest.cgi?missed_cleavages=2&msparams_dir=msparams_mill/&hide_protein_sequence=2&database=NCBIgb_SA300sequence.fasta&seqdb_dir=D:\SeqDB\&enzyme=Trypsin&access_method=Accession+Number&accession_num=AIA27732.1&coverage_map=0+153+40+9+21+79) | 36.67 | 3 |
|  | 6.69E+06 | 0.00E+00 | 0.00E+00 |  |  |  |  |  |  |  |
| 104 | 5 | 0 | 0 | RNA helicase | 56941.3 | 9.48 | [AIA28578.1](http://www.ncbi.nlm.nih.gov/protein/AIA28578.1) | [14.6](http://desktop-0t1rtqn/millbin/msdigest.cgi?missed_cleavages=2&msparams_dir=msparams_mill/&hide_protein_sequence=2&database=NCBIgb_SA300sequence.fasta&seqdb_dir=D:\SeqDB\&enzyme=Trypsin&access_method=Accession+Number&accession_num=AIA28578.1&coverage_map=0+52+11+77+25+62+21+88+17+153) | 36.51 | 5 |
|  | 5.20E+06 | 0.00E+00 | 0.00E+00 |  |  |  |  |  |  |  |
| 105 | 5 | 0 | 0 | septation ring formation regulator EzrA | 66312.4 | 4.78 | [AIA28244.1](http://www.ncbi.nlm.nih.gov/protein/AIA28244.1) | [11.5](http://desktop-0t1rtqn/millbin/msdigest.cgi?missed_cleavages=2&msparams_dir=msparams_mill/&hide_protein_sequence=2&database=NCBIgb_SA300sequence.fasta&seqdb_dir=D:\SeqDB\&enzyme=Trypsin&access_method=Accession+Number&accession_num=AIA28244.1&coverage_map=0+211+11+40+12+206+24+33+18+9) | 36.25 | 5 |
|  | 2.27E+06 | 0.00E+00 | 0.00E+00 |  |  |  |  |  |  |  |
| 106 | 8 | 0 | 0 | 30S ribosomal protein S11 | 13938.7 | 11.4 | [AIA28712.1](http://www.ncbi.nlm.nih.gov/protein/AIA28712.1) | [44.1](http://desktop-0t1rtqn/millbin/msdigest.cgi?missed_cleavages=2&msparams_dir=msparams_mill/&hide_protein_sequence=2&database=NCBIgb_SA300sequence.fasta&seqdb_dir=D:\SeqDB\&enzyme=Trypsin&access_method=Accession+Number&accession_num=AIA28712.1&coverage_map=0+24+29+4+15+26+13+18) | 36.09 | 8 |
|  | 1.00E+07 | 0.00E+00 | 0.00E+00 |  |  |  |  |  |  |  |
| 107 | 4 | 0 | 0 | phosphoribosylaminoimidazolecarboxamide formyltransferase | 54434 | 5.65 | [AIA27565.1](http://www.ncbi.nlm.nih.gov/protein/AIA27565.1) | [12.3](http://desktop-0t1rtqn/millbin/msdigest.cgi?missed_cleavages=2&msparams_dir=msparams_mill/&hide_protein_sequence=2&database=NCBIgb_SA300sequence.fasta&seqdb_dir=D:\SeqDB\&enzyme=Trypsin&access_method=Accession+Number&accession_num=AIA27565.1&coverage_map=0+36+10+322+20+38+31+35) | 35.74 | 4 |
|  | 2.69E+06 | 0.00E+00 | 0.00E+00 |  |  |  |  |  |  |  |
| 108 | 7 | 0 | 0 | acetaldehyde dehydrogenase | 95399.7 | 5.68 | [AIA26738.1](http://www.ncbi.nlm.nih.gov/protein/AIA26738.1) | [8.6](http://desktop-0t1rtqn/millbin/msdigest.cgi?missed_cleavages=2&msparams_dir=msparams_mill/&hide_protein_sequence=2&database=NCBIgb_SA300sequence.fasta&seqdb_dir=D:\SeqDB\&enzyme=Trypsin&access_method=Accession+Number&accession_num=AIA26738.1&coverage_map=0+160+11+160+17+304+28+170+19) | 35.62 | 7 |
|  | 2.72E+06 | 0.00E+00 | 0.00E+00 |  |  |  |  |  |  |  |
| 109 | 4 | 0 | 0 | ribitol-5-phosphate dehydrogenase | 38565.2 | 4.95 | [AIA26835.1](http://www.ncbi.nlm.nih.gov/protein/AIA26835.1) | [24.6](http://desktop-0t1rtqn/millbin/msdigest.cgi?missed_cleavages=2&msparams_dir=msparams_mill/&hide_protein_sequence=2&database=NCBIgb_SA300sequence.fasta&seqdb_dir=D:\SeqDB\&enzyme=Trypsin&access_method=Accession+Number&accession_num=AIA26835.1&coverage_map=0+127+27+79+34+18+23+33) | 34.96 | 4 |
|  | 5.12E+06 | 0.00E+00 | 0.00E+00 |  |  |  |  |  |  |  |
| 110 | 5 | 0 | 0 | lipoate--protein ligase | 37998.1 | 5.2 | [AQR01276.1](http://www.ncbi.nlm.nih.gov/protein/AQR01276.1) | [19.5](http://desktop-0t1rtqn/millbin/msdigest.cgi?missed_cleavages=2&msparams_dir=msparams_mill/&hide_protein_sequence=2&database=NCBIgb_SA300sequence.fasta&seqdb_dir=D:\SeqDB\&enzyme=Trypsin&access_method=Accession+Number&accession_num=AQR01276.1&coverage_map=0+2+22+77+21+21+21+164) | 34.73 | 5 |
|  | 1.95E+06 | 0.00E+00 | 0.00E+00 |  |  |  |  |  |  |  |
| 111 | 1 | 3 | 0 | LPXTG cell wall anchor domain-containing protein | 83451.1 | 9.43 | [QEA17654.1](http://www.ncbi.nlm.nih.gov/protein/QEA17654.1) | [9.9](http://desktop-0t1rtqn/millbin/msdigest.cgi?missed_cleavages=2&msparams_dir=msparams_mill/&hide_protein_sequence=2&database=NCBIgb_SA300sequence.fasta&seqdb_dir=D:\SeqDB\&enzyme=Trypsin&access_method=Accession+Number&accession_num=QEA17654.1&coverage_map=0+125+12+153+17+191+19+22+29+204) | 34.68 | 1 |
|  | 4.12E+04 | 1.69E+06 | 0.00E+00 |  |  |  |  |  |  |  |
| 112 | 3 | 1 | 1 | phosphoglyceromutase | 26736.5 | 5.23 | [AIA28897.1](http://www.ncbi.nlm.nih.gov/protein/AIA28897.1) | [31.1](http://desktop-0t1rtqn/millbin/msdigest.cgi?missed_cleavages=2&msparams_dir=msparams_mill/&hide_protein_sequence=2&database=NCBIgb_SA300sequence.fasta&seqdb_dir=D:\SeqDB\&enzyme=Trypsin&access_method=Accession+Number&accession_num=AIA28897.1&coverage_map=0+17+21+120+29+20+21) | 34.33 | 3 |
|  | 6.46E+05 | 3.18E+03 | 8.73E+05 |  |  |  |  |  |  |  |
| 113 | 5 | 0 | 0 | octopine dehydrogenase | 40946.5 | 4.83 | [KMR33176.1](http://www.ncbi.nlm.nih.gov/protein/KMR33176.1) | [15.5](http://desktop-0t1rtqn/millbin/msdigest.cgi?missed_cleavages=2&msparams_dir=msparams_mill/&hide_protein_sequence=2&database=NCBIgb_SA300sequence.fasta&seqdb_dir=D:\SeqDB\&enzyme=Trypsin&access_method=Accession+Number&accession_num=KMR33176.1&coverage_map=0+59+13+242+43+3) | 34.28 | 5 |
|  | 3.76E+06 | 0.00E+00 | 0.00E+00 |  |  |  |  |  |  |  |
| 114 | 2 | 4 | 7 | 30S ribosomal protein S5 | 17742.2 | 9.88 | [AIA28720.1](http://www.ncbi.nlm.nih.gov/protein/AIA28720.1) | [28.9](http://desktop-0t1rtqn/millbin/msdigest.cgi?missed_cleavages=2&msparams_dir=msparams_mill/&hide_protein_sequence=2&database=NCBIgb_SA300sequence.fasta&seqdb_dir=D:\SeqDB\&enzyme=Trypsin&access_method=Accession+Number&accession_num=AIA28720.1&coverage_map=0+88+38+12+10+18) | 34.24 | 2 |
|  | 8.49E+06 | 3.78E+06 | 7.25E+06 |  |  |  |  |  |  |  |
| 115 | 0 | 8 | 3 | inhibitor | 13066.8 | 9.33 | [AIA28458.1](http://www.ncbi.nlm.nih.gov/protein/AIA28458.1) | [26.7](http://desktop-0t1rtqn/millbin/msdigest.cgi?missed_cleavages=2&msparams_dir=msparams_mill/&hide_protein_sequence=2&database=NCBIgb_SA300sequence.fasta&seqdb_dir=D:\SeqDB\&enzyme=Trypsin&access_method=Accession+Number&accession_num=AIA28458.1&coverage_map=0+51+21+31+10+3) | 34.12 | 0 |
|  | 0.00E+00 | 2.69E+07 | 1.87E+06 |  |  |  |  |  |  |  |
| 116 | 2 | 4 | 6 | transglycosylase | 24202.8 | 6.11 | [AIA29042.1](http://www.ncbi.nlm.nih.gov/protein/AIA29042.1) | [25.7](http://desktop-0t1rtqn/millbin/msdigest.cgi?missed_cleavages=2&msparams_dir=msparams_mill/&hide_protein_sequence=2&database=NCBIgb_SA300sequence.fasta&seqdb_dir=D:\SeqDB\&enzyme=Trypsin&access_method=Accession+Number&accession_num=AIA29042.1&coverage_map=0+147+21+14+39+12) | 34.11 | 2 |
|  | 4.35E+06 | 2.29E+07 | 2.80E+07 |  |  |  |  |  |  |  |
| 117 | 6 | 0 | 2 | GTP-binding protein TypA | 69308.4 | 4.94 | [AIA27599.1](http://www.ncbi.nlm.nih.gov/protein/AIA27599.1) | [7.3](http://desktop-0t1rtqn/millbin/msdigest.cgi?missed_cleavages=2&msparams_dir=msparams_mill/&hide_protein_sequence=2&database=NCBIgb_SA300sequence.fasta&seqdb_dir=D:\SeqDB\&enzyme=Trypsin&access_method=Accession+Number&accession_num=AIA27599.1&coverage_map=0+93+20+451+25+26) | 33.59 | 6 |
|  | 8.66E+06 | 0.00E+00 | 1.17E+05 |  |  |  |  |  |  |  |
| 118 | 5 | 0 | 0 | Asp-tRNA(Asn)/Glu-tRNA(Gln) amidotransferase GatCAB subunit A | 53019.1 | 5.02 | [OWU40549.1](http://www.ncbi.nlm.nih.gov/protein/OWU40549.1) | [9.2](http://desktop-0t1rtqn/millbin/msdigest.cgi?missed_cleavages=2&msparams_dir=msparams_mill/&hide_protein_sequence=2&database=NCBIgb_SA300sequence.fasta&seqdb_dir=D:\SeqDB\&enzyme=Trypsin&access_method=Accession+Number&accession_num=OWU40549.1&coverage_map=0+27+14+53+16+178+15+182) | 33.56 | 5 |
|  | 1.58E+06 | 0.00E+00 | 0.00E+00 |  |  |  |  |  |  |  |
| 119 | 0 | 4 | 0 | fibrinogen-binding protein | 12626.5 | 10.41 | [ALK38976.1](http://www.ncbi.nlm.nih.gov/protein/ALK38976.1) | [23.8](http://desktop-0t1rtqn/millbin/msdigest.cgi?missed_cleavages=2&msparams_dir=msparams_mill/&hide_protein_sequence=2&database=NCBIgb_SA300sequence.fasta&seqdb_dir=D:\SeqDB\&enzyme=Trypsin&access_method=Accession+Number&accession_num=ALK38976.1&coverage_map=0+60+6+13+13+5+7+5) | 33.39 | 0 |
|  | 0.00E+00 | 2.07E+06 | 0.00E+00 |  |  |  |  |  |  |  |
| 120 | 3 | 0 | 2 | 50S ribosomal protein L13 | 16333.1 | 9.3 | [AIA28705.1](http://www.ncbi.nlm.nih.gov/protein/AIA28705.1) | [29.6](http://desktop-0t1rtqn/millbin/msdigest.cgi?missed_cleavages=2&msparams_dir=msparams_mill/&hide_protein_sequence=2&database=NCBIgb_SA300sequence.fasta&seqdb_dir=D:\SeqDB\&enzyme=Trypsin&access_method=Accession+Number&accession_num=AIA28705.1&coverage_map=0+15+23+4+20+83) | 33.38 | 3 |
|  | 1.64E+06 | 0.00E+00 | 2.74E+05 |  |  |  |  |  |  |  |
| 121 | 4 | 0 | 0 | dihydroxyacetone kinase | 21260.6 | 4.77 | [AIA27229.1](http://www.ncbi.nlm.nih.gov/protein/AIA27229.1) | [26.8](http://desktop-0t1rtqn/millbin/msdigest.cgi?missed_cleavages=2&msparams_dir=msparams_mill/&hide_protein_sequence=2&database=NCBIgb_SA300sequence.fasta&seqdb_dir=D:\SeqDB\&enzyme=Trypsin&access_method=Accession+Number&accession_num=AIA27229.1&coverage_map=0+85+28+57+24) | 33.32 | 4 |
|  | 6.31E+05 | 0.00E+00 | 0.00E+00 |  |  |  |  |  |  |  |
| 122 | 4 | 0 | 2 | 50S ribosomal protein L21 | 11332.8 | 9.84 | [AIA28183.1](http://www.ncbi.nlm.nih.gov/protein/AIA28183.1) | [35.2](http://desktop-0t1rtqn/millbin/msdigest.cgi?missed_cleavages=2&msparams_dir=msparams_mill/&hide_protein_sequence=2&database=NCBIgb_SA300sequence.fasta&seqdb_dir=D:\SeqDB\&enzyme=Trypsin&access_method=Accession+Number&accession_num=AIA28183.1&coverage_map=1+10+27+27+38) | 33.01 | 4 |
|  | 7.02E+05 | 0.00E+00 | 5.84E+04 |  |  |  |  |  |  |  |
| 123 | 4 | 0 | 1 | zinc metalloprotease | 77954.2 | 5.37 | [AIA27068.1](http://www.ncbi.nlm.nih.gov/protein/AIA27068.1) | [7.3](http://desktop-0t1rtqn/millbin/msdigest.cgi?missed_cleavages=2&msparams_dir=msparams_mill/&hide_protein_sequence=2&database=NCBIgb_SA300sequence.fasta&seqdb_dir=D:\SeqDB\&enzyme=Trypsin&access_method=Accession+Number&accession_num=AIA27068.1&coverage_map=0+365+22+192+29+89) | 32.98 | 4 |
|  | 3.62E+06 | 0.00E+00 | 5.69E+05 |  |  |  |  |  |  |  |
| 124 | 6 | 0 | 0 | translation initiation factor IF-2 | 78041.5 | 5.07 | [KMR41288.1](http://www.ncbi.nlm.nih.gov/protein/KMR41288.1) | [7](http://desktop-0t1rtqn/millbin/msdigest.cgi?missed_cleavages=2&msparams_dir=msparams_mill/&hide_protein_sequence=2&database=NCBIgb_SA300sequence.fasta&seqdb_dir=D:\SeqDB\&enzyme=Trypsin&access_method=Accession+Number&accession_num=KMR41288.1&coverage_map=0+396+27+105+9+50+14+104) | 32.98 | 6 |
|  | 9.59E+06 | 0.00E+00 | 0.00E+00 |  |  |  |  |  |  |  |
| 125 | 5 | 0 | 0 | GTP pyrophosphokinase | 84735.7 | 8.49 | [AIA28170.1](http://www.ncbi.nlm.nih.gov/protein/AIA28170.1) | [8.9](http://desktop-0t1rtqn/millbin/msdigest.cgi?missed_cleavages=2&msparams_dir=msparams_mill/&hide_protein_sequence=2&database=NCBIgb_SA300sequence.fasta&seqdb_dir=D:\SeqDB\&enzyme=Trypsin&access_method=Accession+Number&accession_num=AIA28170.1&coverage_map=1+22+557+25+66+20+46) | 32.37 | 5 |
|  | 1.80E+06 | 0.00E+00 | 0.00E+00 |  |  |  |  |  |  |  |
| 126 | 0 | 3 | 3 | secretory antigen precursor | 17758.8 | 5.77 | [ALK42678.1](http://www.ncbi.nlm.nih.gov/protein/ALK42678.1) | [34.3](http://desktop-0t1rtqn/millbin/msdigest.cgi?missed_cleavages=2&msparams_dir=msparams_mill/&hide_protein_sequence=2&database=NCBIgb_SA300sequence.fasta&seqdb_dir=D:\SeqDB\&enzyme=Trypsin&access_method=Accession+Number&accession_num=ALK42678.1&coverage_map=0+111+58) | 32.06 | 0 |
|  | 0.00E+00 | 6.59E+06 | 1.80E+06 |  |  |  |  |  |  |  |
| 127 | 2 | 0 | 1 | glycine C-acetyltransferase | 43004.9 | 5.14 | [OWU55298.1](http://www.ncbi.nlm.nih.gov/protein/OWU55298.1) | [25.3](http://desktop-0t1rtqn/millbin/msdigest.cgi?missed_cleavages=2&msparams_dir=msparams_mill/&hide_protein_sequence=2&database=NCBIgb_SA300sequence.fasta&seqdb_dir=D:\SeqDB\&enzyme=Trypsin&access_method=Accession+Number&accession_num=OWU55298.1&coverage_map=0+79+45+68+34+87+21+61) | 32.04 | 2 |
|  | 2.63E+04 | 0.00E+00 | 8.04E+03 |  |  |  |  |  |  |  |
| 128 | 0 | 5 | 2 | fibrinogen-binding protein | 18764.2 | 9.82 | [AIA27644.1](http://www.ncbi.nlm.nih.gov/protein/AIA27644.1) | [29](http://desktop-0t1rtqn/millbin/msdigest.cgi?missed_cleavages=2&msparams_dir=msparams_mill/&hide_protein_sequence=2&database=NCBIgb_SA300sequence.fasta&seqdb_dir=D:\SeqDB\&enzyme=Trypsin&access_method=Accession+Number&accession_num=AIA27644.1&coverage_map=0+71+29+10+9+16+10+20) | 31.44 | 0 |
|  | 0.00E+00 | 4.88E+06 | 3.20E+05 |  |  |  |  |  |  |  |
| 129 | 0 | 3 | 0 | type VII secretion protein EsaA | 114882 | 6.08 | [KMS16730.1](http://www.ncbi.nlm.nih.gov/protein/KMS16730.1) | [6](http://desktop-0t1rtqn/millbin/msdigest.cgi?missed_cleavages=2&msparams_dir=msparams_mill/&hide_protein_sequence=2&database=NCBIgb_SA300sequence.fasta&seqdb_dir=D:\SeqDB\&enzyme=Trypsin&access_method=Accession+Number&accession_num=KMS16730.1&coverage_map=0+192+28+11+11+229+22+516) | 30.9 | 0 |
|  | 0.00E+00 | 1.02E+05 | 0.00E+00 |  |  |  |  |  |  |  |
| 130 | 4 | 0 | 2 | deoxyribose-phosphate aldolase | 23616.2 | 4.68 | [AIA26726.1](http://www.ncbi.nlm.nih.gov/protein/AIA26726.1) | [28.1](http://desktop-0t1rtqn/millbin/msdigest.cgi?missed_cleavages=2&msparams_dir=msparams_mill/&hide_protein_sequence=2&database=NCBIgb_SA300sequence.fasta&seqdb_dir=D:\SeqDB\&enzyme=Trypsin&access_method=Accession+Number&accession_num=AIA26726.1&coverage_map=0+49+49+3+13+106) | 30.75 | 4 |
|  | 2.42E+06 | 0.00E+00 | 2.10E+06 |  |  |  |  |  |  |  |
| 131 | 0 | 5 | 1 | succinyl-diaminopimelate desuccinylase | 40490.4 | 9.45 | [AIA28518.1](http://www.ncbi.nlm.nih.gov/protein/AIA28518.1) | [9.9](http://desktop-0t1rtqn/millbin/msdigest.cgi?missed_cleavages=2&msparams_dir=msparams_mill/&hide_protein_sequence=2&database=NCBIgb_SA300sequence.fasta&seqdb_dir=D:\SeqDB\&enzyme=Trypsin&access_method=Accession+Number&accession_num=AIA28518.1&coverage_map=0+234+25+62+10+20) | 30.37 | 0 |
|  | 0.00E+00 | 2.50E+06 | 6.88E+04 |  |  |  |  |  |  |  |
| 132 | 0 | 1 | 4 | 50S ribosomal protein L15 | 15626.6 | 10.28 | [KMR74388.1](http://www.ncbi.nlm.nih.gov/protein/KMR74388.1) | [41.7](http://desktop-0t1rtqn/millbin/msdigest.cgi?missed_cleavages=2&msparams_dir=msparams_mill/&hide_protein_sequence=2&database=NCBIgb_SA300sequence.fasta&seqdb_dir=D:\SeqDB\&enzyme=Trypsin&access_method=Accession+Number&accession_num=KMR74388.1&coverage_map=0+41+18+13+31+24+12+7) | 30.27 | 0 |
|  | 0.00E+00 | 5.50E+05 | 3.12E+06 |  |  |  |  |  |  |  |
| 133 | 4 | 0 | 0 | DNA polymerase III subunit beta | 41969.9 | 4.66 | [AIA26594.1](http://www.ncbi.nlm.nih.gov/protein/AIA26594.1) | [15.1](http://desktop-0t1rtqn/millbin/msdigest.cgi?missed_cleavages=2&msparams_dir=msparams_mill/&hide_protein_sequence=2&database=NCBIgb_SA300sequence.fasta&seqdb_dir=D:\SeqDB\&enzyme=Trypsin&access_method=Accession+Number&accession_num=AIA26594.1&coverage_map=0+213+21+105+36+2) | 29.96 | 4 |
|  | 1.91E+06 | 0.00E+00 | 0.00E+00 |  |  |  |  |  |  |  |
| 134 | 4 | 0 | 0 | phosphoglucosamine mutase | 49426.8 | 4.65 | [AQQ90729.1](http://www.ncbi.nlm.nih.gov/protein/AQQ90729.1) | [9.7](http://desktop-0t1rtqn/millbin/msdigest.cgi?missed_cleavages=2&msparams_dir=msparams_mill/&hide_protein_sequence=2&database=NCBIgb_SA300sequence.fasta&seqdb_dir=D:\SeqDB\&enzyme=Trypsin&access_method=Accession+Number&accession_num=AQQ90729.1&coverage_map=0+166+11+96+21+140+12+5) | 29.88 | 4 |
|  | 1.65E+05 | 0.00E+00 | 0.00E+00 |  |  |  |  |  |  |  |
| 135 | 6 | 0 | 0 | nucleoside-triphosphate diphosphatase | 21459.8 | 4.89 | [AIA27639.1](http://www.ncbi.nlm.nih.gov/protein/AIA27639.1) | [25.6](http://desktop-0t1rtqn/millbin/msdigest.cgi?missed_cleavages=2&msparams_dir=msparams_mill/&hide_protein_sequence=2&database=NCBIgb_SA300sequence.fasta&seqdb_dir=D:\SeqDB\&enzyme=Trypsin&access_method=Accession+Number&accession_num=AIA27639.1&coverage_map=0+18+34+127+16) | 29.71 | 6 |
|  | 3.43E+06 | 0.00E+00 | 0.00E+00 |  |  |  |  |  |  |  |
| 136 | 3 | 1 | 3 | universal stress protein UspA | 15229.4 | 9.52 | [AQQ90263.1](http://www.ncbi.nlm.nih.gov/protein/AQQ90263.1) | [36.4](http://desktop-0t1rtqn/millbin/msdigest.cgi?missed_cleavages=2&msparams_dir=msparams_mill/&hide_protein_sequence=2&database=NCBIgb_SA300sequence.fasta&seqdb_dir=D:\SeqDB\&enzyme=Trypsin&access_method=Accession+Number&accession_num=AQQ90263.1&coverage_map=0+3+11+10+24+21+15+53) | 29.47 | 3 |
|  | 4.30E+06 | 6.68E+04 | 2.76E+06 |  |  |  |  |  |  |  |
| 137 | 2 | 0 | 1 | hyperosmolarity resistance protein Ebh | 1095432 | 5.87 | [QCV67987.1](http://www.ncbi.nlm.nih.gov/protein/QCV67987.1) | [0.8](http://desktop-0t1rtqn/millbin/msdigest.cgi?missed_cleavages=2&msparams_dir=msparams_mill/&hide_protein_sequence=2&database=NCBIgb_SA300sequence.fasta&seqdb_dir=D:\SeqDB\&enzyme=Trypsin&access_method=Accession+Number&accession_num=QCV67987.1&coverage_map=0+2640+29+524+24+3814+32+3106) | 28.93 | 2 |
|  | 7.46E+05 | 0.00E+00 | 1.33E+04 |  |  |  |  |  |  |  |
| 138 | 4 | 0 | 1 | peptidylprolyl isomerase | 21618.8 | 4.57 | [AIA27451.1](http://www.ncbi.nlm.nih.gov/protein/AIA27451.1) | [34](http://desktop-0t1rtqn/millbin/msdigest.cgi?missed_cleavages=2&msparams_dir=msparams_mill/&hide_protein_sequence=2&database=NCBIgb_SA300sequence.fasta&seqdb_dir=D:\SeqDB\&enzyme=Trypsin&access_method=Accession+Number&accession_num=AIA27451.1&coverage_map=0+121+26+9+41) | 28.82 | 4 |
|  | 3.51E+06 | 0.00E+00 | 3.80E+05 |  |  |  |  |  |  |  |
| 139 | 1 | 1 | 2 | ferritin | 19645.3 | 4.67 | [AIA28414.1](http://www.ncbi.nlm.nih.gov/protein/AIA28414.1) | [13.8](http://desktop-0t1rtqn/millbin/msdigest.cgi?missed_cleavages=2&msparams_dir=msparams_mill/&hide_protein_sequence=2&database=NCBIgb_SA300sequence.fasta&seqdb_dir=D:\SeqDB\&enzyme=Trypsin&access_method=Accession+Number&accession_num=AIA28414.1&coverage_map=0+77+23+66) | 28.61 | 1 |
|  | 7.35E+05 | 9.57E+05 | 1.37E+06 |  |  |  |  |  |  |  |
| 140 | 1 | 0 | 2 | 50S ribosomal protein L3 | 23717.8 | 9.8 | [AIA28737.1](http://www.ncbi.nlm.nih.gov/protein/AIA28737.1) | [10.4](http://desktop-0t1rtqn/millbin/msdigest.cgi?missed_cleavages=2&msparams_dir=msparams_mill/&hide_protein_sequence=2&database=NCBIgb_SA300sequence.fasta&seqdb_dir=D:\SeqDB\&enzyme=Trypsin&access_method=Accession+Number&accession_num=AIA28737.1&coverage_map=0+8+23+189) | 28.45 | 1 |
|  | 2.47E+05 | 0.00E+00 | 5.26E+05 |  |  |  |  |  |  |  |
| 141 | 4 | 0 | 0 | transcription elongation factor NusA | 43807.4 | 4.59 | [AKK60956.1](http://www.ncbi.nlm.nih.gov/protein/AKK60956.1) | [13.2](http://desktop-0t1rtqn/millbin/msdigest.cgi?missed_cleavages=2&msparams_dir=msparams_mill/&hide_protein_sequence=2&database=NCBIgb_SA300sequence.fasta&seqdb_dir=D:\SeqDB\&enzyme=Trypsin&access_method=Accession+Number&accession_num=AKK60956.1&coverage_map=0+21+17+247+35+71) | 28.23 | 4 |
|  | 2.31E+06 | 0.00E+00 | 0.00E+00 |  |  |  |  |  |  |  |
| 142 | 5 | 0 | 3 | peroxidase | 18731.8 | 4.73 | [KMR43288.1](http://www.ncbi.nlm.nih.gov/protein/KMR43288.1) | [36](http://desktop-0t1rtqn/millbin/msdigest.cgi?missed_cleavages=2&msparams_dir=msparams_mill/&hide_protein_sequence=2&database=NCBIgb_SA300sequence.fasta&seqdb_dir=D:\SeqDB\&enzyme=Trypsin&access_method=Accession+Number&accession_num=KMR43288.1&coverage_map=0+14+31+83+10+4+20+7) | 28.09 | 5 |
|  | 4.08E+06 | 0.00E+00 | 2.32E+06 |  |  |  |  |  |  |  |
| 143 | 2 | 0 | 0 | sodium:proton antiporter | 47446.7 | 6.21 | [AHZ98735.1](http://www.ncbi.nlm.nih.gov/protein/AHZ98735.1) | [20.5](http://desktop-0t1rtqn/millbin/msdigest.cgi?missed_cleavages=2&msparams_dir=msparams_mill/&hide_protein_sequence=2&database=NCBIgb_SA300sequence.fasta&seqdb_dir=D:\SeqDB\&enzyme=Trypsin&access_method=Accession+Number&accession_num=AHZ98735.1&coverage_map=1+43+102+48+245) | 27.85 | 2 |
|  | 8.25E+04 | 0.00E+00 | 0.00E+00 |  |  |  |  |  |  |  |
| 144 | 4 | 0 | 0 | acyl--CoA ligase | 60090.1 | 5.49 | [AKK59716.1](http://www.ncbi.nlm.nih.gov/protein/AKK59716.1) | [9](http://desktop-0t1rtqn/millbin/msdigest.cgi?missed_cleavages=2&msparams_dir=msparams_mill/&hide_protein_sequence=2&database=NCBIgb_SA300sequence.fasta&seqdb_dir=D:\SeqDB\&enzyme=Trypsin&access_method=Accession+Number&accession_num=AKK59716.1&coverage_map=0+395+21+45+17+17+10+27) | 27.81 | 4 |
|  | 6.67E+05 | 0.00E+00 | 0.00E+00 |  |  |  |  |  |  |  |
| 145 | 3 | 0 | 0 | uridylate kinase | 26316.1 | 5.99 | [AIA27744.1](http://www.ncbi.nlm.nih.gov/protein/AIA27744.1) | [22](http://desktop-0t1rtqn/millbin/msdigest.cgi?missed_cleavages=2&msparams_dir=msparams_mill/&hide_protein_sequence=2&database=NCBIgb_SA300sequence.fasta&seqdb_dir=D:\SeqDB\&enzyme=Trypsin&access_method=Accession+Number&accession_num=AIA27744.1&coverage_map=0+23+10+38+30+49+13+77) | 27.75 | 3 |
|  | 6.96E+05 | 0.00E+00 | 0.00E+00 |  |  |  |  |  |  |  |
| 146 | 5 | 0 | 1 | cell division protein DivIVA | 23513.6 | 4.91 | [AIA27678.1](http://www.ncbi.nlm.nih.gov/protein/AIA27678.1) | [11.2](http://desktop-0t1rtqn/millbin/msdigest.cgi?missed_cleavages=2&msparams_dir=msparams_mill/&hide_protein_sequence=2&database=NCBIgb_SA300sequence.fasta&seqdb_dir=D:\SeqDB\&enzyme=Trypsin&access_method=Accession+Number&accession_num=AIA27678.1&coverage_map=0+15+23+167) | 27.4 | 5 |
|  | 1.07E+07 | 0.00E+00 | 3.24E+05 |  |  |  |  |  |  |  |
| 147 | 2 | 0 | 2 | dihydrolipoamide succinyltransferase | 46729.7 | 4.85 | [AIA27900.1](http://www.ncbi.nlm.nih.gov/protein/AIA27900.1) | [10.9](http://desktop-0t1rtqn/millbin/msdigest.cgi?missed_cleavages=2&msparams_dir=msparams_mill/&hide_protein_sequence=2&database=NCBIgb_SA300sequence.fasta&seqdb_dir=D:\SeqDB\&enzyme=Trypsin&access_method=Accession+Number&accession_num=AIA27900.1&coverage_map=0+201+31+74+15+101) | 26.98 | 2 |
|  | 9.86E+05 | 0.00E+00 | 4.45E+04 |  |  |  |  |  |  |  |
| 148 | 4 | 0 | 2 | enoyl-ACP reductase | 28039.4 | 5.63 | [AKK60711.1](http://www.ncbi.nlm.nih.gov/protein/AKK60711.1) | [17.1](http://desktop-0t1rtqn/millbin/msdigest.cgi?missed_cleavages=2&msparams_dir=msparams_mill/&hide_protein_sequence=2&database=NCBIgb_SA300sequence.fasta&seqdb_dir=D:\SeqDB\&enzyme=Trypsin&access_method=Accession+Number&accession_num=AKK60711.1&coverage_map=0+50+32+90+12+72) | 26.95 | 4 |
|  | 3.64E+06 | 0.00E+00 | 1.10E+06 |  |  |  |  |  |  |  |
| 149 | 4 | 0 | 0 | NADH-dependent flavin oxidoreductase | 42126.7 | 5.24 | [QCV67503.1](http://www.ncbi.nlm.nih.gov/protein/QCV67503.1) | [11.2](http://desktop-0t1rtqn/millbin/msdigest.cgi?missed_cleavages=2&msparams_dir=msparams_mill/&hide_protein_sequence=2&database=NCBIgb_SA300sequence.fasta&seqdb_dir=D:\SeqDB\&enzyme=Trypsin&access_method=Accession+Number&accession_num=QCV67503.1&coverage_map=0+290+42+43) | 26.92 | 4 |
|  | 4.57E+06 | 0.00E+00 | 0.00E+00 |  |  |  |  |  |  |  |
| 150 | 4 | 0 | 0 | pyridoxal biosynthesis protein | 32106.3 | 5.1 | [AIA27076.1](http://www.ncbi.nlm.nih.gov/protein/AIA27076.1) | [14.2](http://desktop-0t1rtqn/millbin/msdigest.cgi?missed_cleavages=2&msparams_dir=msparams_mill/&hide_protein_sequence=2&database=NCBIgb_SA300sequence.fasta&seqdb_dir=D:\SeqDB\&enzyme=Trypsin&access_method=Accession+Number&accession_num=AIA27076.1&coverage_map=0+19+14+33+16+195+12+6) | 26.79 | 4 |
|  | 4.89E+05 | 0.00E+00 | 0.00E+00 |  |  |  |  |  |  |  |
| 151 | 3 | 0 | 0 | DNA-directed RNA polymerase subunit alpha | 35068.1 | 4.69 | [AIA28711.1](http://www.ncbi.nlm.nih.gov/protein/AIA28711.1) | [16.8](http://desktop-0t1rtqn/millbin/msdigest.cgi?missed_cleavages=2&msparams_dir=msparams_mill/&hide_protein_sequence=2&database=NCBIgb_SA300sequence.fasta&seqdb_dir=D:\SeqDB\&enzyme=Trypsin&access_method=Accession+Number&accession_num=AIA28711.1&coverage_map=0+22+8+154+31+32+14+53) | 26.67 | 3 |
|  | 1.18E+06 | 0.00E+00 | 0.00E+00 |  |  |  |  |  |  |  |
| 152 | 2 | 0 | 0 | 2-C-methyl-D-erythritol 4-phosphate cytidylyltransferase | 26723.2 | 5.43 | [KMS12028.1](http://www.ncbi.nlm.nih.gov/protein/KMS12028.1) | [12.1](http://desktop-0t1rtqn/millbin/msdigest.cgi?missed_cleavages=2&msparams_dir=msparams_mill/&hide_protein_sequence=2&database=NCBIgb_SA300sequence.fasta&seqdb_dir=D:\SeqDB\&enzyme=Trypsin&access_method=Accession+Number&accession_num=KMS12028.1&coverage_map=0+120+29+89) | 26.57 | 2 |
|  | 5.14E+05 | 0.00E+00 | 0.00E+00 |  |  |  |  |  |  |  |
| 153 | 3 | 0 | 0 | ATP-dependent Clp protease ATP-binding subunit | 91150.8 | 5.51 | [OWU42251.1](http://www.ncbi.nlm.nih.gov/protein/OWU42251.1) | [5.2](http://desktop-0t1rtqn/millbin/msdigest.cgi?missed_cleavages=2&msparams_dir=msparams_mill/&hide_protein_sequence=2&database=NCBIgb_SA300sequence.fasta&seqdb_dir=D:\SeqDB\&enzyme=Trypsin&access_method=Accession+Number&accession_num=OWU42251.1&coverage_map=0+238+14+209+29+328) | 26.13 | 3 |
|  | 3.81E+06 | 0.00E+00 | 0.00E+00 |  |  |  |  |  |  |  |
| 154 | 3 | 0 | 0 | serine/threonine protein phosphatase | 38596.6 | 5.44 | [AIA28565.1](http://www.ncbi.nlm.nih.gov/protein/AIA28565.1) | [15.3](http://desktop-0t1rtqn/millbin/msdigest.cgi?missed_cleavages=2&msparams_dir=msparams_mill/&hide_protein_sequence=2&database=NCBIgb_SA300sequence.fasta&seqdb_dir=D:\SeqDB\&enzyme=Trypsin&access_method=Accession+Number&accession_num=AIA28565.1&coverage_map=0+130+26+96+25+56) | 26.05 | 3 |
|  | 4.27E+06 | 0.00E+00 | 0.00E+00 |  |  |  |  |  |  |  |
| 155 | 1 | 0 | 2 | type VII secretion protein EsxA | 11040.1 | 4.48 | [AQQ82892.1](http://www.ncbi.nlm.nih.gov/protein/AQQ82892.1) | [37.1](http://desktop-0t1rtqn/millbin/msdigest.cgi?missed_cleavages=2&msparams_dir=msparams_mill/&hide_protein_sequence=2&database=NCBIgb_SA300sequence.fasta&seqdb_dir=D:\SeqDB\&enzyme=Trypsin&access_method=Accession+Number&accession_num=AQQ82892.1&coverage_map=0+61+36) | 26.03 | 1 |
|  | 1.91E+05 | 0.00E+00 | 1.54E+05 |  |  |  |  |  |  |  |
| 156 | 4 | 0 | 0 | ribonucleotide-diphosphate reductase subunit beta | 37512.7 | 4.77 | [AIA27309.1](http://www.ncbi.nlm.nih.gov/protein/AIA27309.1) | [14.5](http://desktop-0t1rtqn/millbin/msdigest.cgi?missed_cleavages=2&msparams_dir=msparams_mill/&hide_protein_sequence=2&database=NCBIgb_SA300sequence.fasta&seqdb_dir=D:\SeqDB\&enzyme=Trypsin&access_method=Accession+Number&accession_num=AIA27309.1&coverage_map=0+97+30+114+17+65) | 25.8 | 4 |
|  | 9.15E+06 | 0.00E+00 | 0.00E+00 |  |  |  |  |  |  |  |
| 157 | 2 | 0 | 0 | lactate dehydrogenase | 36738 | 5.1 | [AIA28997.1](http://www.ncbi.nlm.nih.gov/protein/AIA28997.1) | [12.1](http://desktop-0t1rtqn/millbin/msdigest.cgi?missed_cleavages=2&msparams_dir=msparams_mill/&hide_protein_sequence=2&database=NCBIgb_SA300sequence.fasta&seqdb_dir=D:\SeqDB\&enzyme=Trypsin&access_method=Accession+Number&accession_num=AIA28997.1&coverage_map=0+90+29+26+11+174) | 25.43 | 2 |
|  | 3.86E+04 | 0.00E+00 | 0.00E+00 |  |  |  |  |  |  |  |
| 158 | 4 | 1 | 0 | iron ABC transporter ATP-binding protein | 28317 | 4.9 | [KMS47512.1](http://www.ncbi.nlm.nih.gov/protein/KMS47512.1) | [13.8](http://desktop-0t1rtqn/millbin/msdigest.cgi?missed_cleavages=2&msparams_dir=msparams_mill/&hide_protein_sequence=2&database=NCBIgb_SA300sequence.fasta&seqdb_dir=D:\SeqDB\&enzyme=Trypsin&access_method=Accession+Number&accession_num=KMS47512.1&coverage_map=0+60+18+88+17+70) | 25.4 | 4 |
|  | 6.72E+06 | 1.24E+06 | 0.00E+00 |  |  |  |  |  |  |  |
| 159 | 4 | 0 | 1 | pyruvate oxidase | 63955.4 | 6.91 | [OWU41042.1](http://www.ncbi.nlm.nih.gov/protein/OWU41042.1) | [9.6](http://desktop-0t1rtqn/millbin/msdigest.cgi?missed_cleavages=2&msparams_dir=msparams_mill/&hide_protein_sequence=2&database=NCBIgb_SA300sequence.fasta&seqdb_dir=D:\SeqDB\&enzyme=Trypsin&access_method=Accession+Number&accession_num=OWU41042.1&coverage_map=0+91+21+393+35+39) | 25.24 | 4 |
|  | 2.80E+06 | 0.00E+00 | 2.04E+05 |  |  |  |  |  |  |  |
| 160 | 3 | 0 | 0 | hypothetical protein EX97_03480 | 26320 | 4.63 | [AIA27247.1](http://www.ncbi.nlm.nih.gov/protein/AIA27247.1) | [18](http://desktop-0t1rtqn/millbin/msdigest.cgi?missed_cleavages=2&msparams_dir=msparams_mill/&hide_protein_sequence=2&database=NCBIgb_SA300sequence.fasta&seqdb_dir=D:\SeqDB\&enzyme=Trypsin&access_method=Accession+Number&accession_num=AIA27247.1&coverage_map=0+83+22+112+21) | 25.14 | 3 |
|  | 6.17E+06 | 0.00E+00 | 0.00E+00 |  |  |  |  |  |  |  |
| 161 | 1 | 0 | 1 | capsular biosynthesis protein | 25284.4 | 8.81 | [AIA26740.1](http://www.ncbi.nlm.nih.gov/protein/AIA26740.1) | [16.6](http://desktop-0t1rtqn/millbin/msdigest.cgi?missed_cleavages=2&msparams_dir=msparams_mill/&hide_protein_sequence=2&database=NCBIgb_SA300sequence.fasta&seqdb_dir=D:\SeqDB\&enzyme=Trypsin&access_method=Accession+Number&accession_num=AIA26740.1&coverage_map=0+26+16+108+22+56) | 25.08 | 1 |
|  | 1.47E+05 | 0.00E+00 | 1.56E+04 |  |  |  |  |  |  |  |
| 162 | 3 | 0 | 0 | 50S ribosomal protein L25 | 23787.3 | 4.39 | [AIA27058.1](http://www.ncbi.nlm.nih.gov/protein/AIA27058.1) | [10.5](http://desktop-0t1rtqn/millbin/msdigest.cgi?missed_cleavages=2&msparams_dir=msparams_mill/&hide_protein_sequence=2&database=NCBIgb_SA300sequence.fasta&seqdb_dir=D:\SeqDB\&enzyme=Trypsin&access_method=Accession+Number&accession_num=AIA27058.1&coverage_map=0+57+11+3+12+134) | 24.99 | 3 |
|  | 1.40E+06 | 0.00E+00 | 0.00E+00 |  |  |  |  |  |  |  |
| 163 | 0 | 3 | 0 | peptigoglycan-binding protein LysM | 55949.8 | 5.58 | [ALK35437.1](http://www.ncbi.nlm.nih.gov/protein/ALK35437.1) | [8.7](http://desktop-0t1rtqn/millbin/msdigest.cgi?missed_cleavages=2&msparams_dir=msparams_mill/&hide_protein_sequence=2&database=NCBIgb_SA300sequence.fasta&seqdb_dir=D:\SeqDB\&enzyme=Trypsin&access_method=Accession+Number&accession_num=ALK35437.1&coverage_map=0+73+23+154+22+240) | 24.92 | 0 |
|  | 0.00E+00 | 5.51E+05 | 0.00E+00 |  |  |  |  |  |  |  |
| 164 | 0 | 2 | 0 | bi-component leukocidin LukGH subunit G | 38857.5 | 8.66 | [QCV68685.1](http://www.ncbi.nlm.nih.gov/protein/QCV68685.1) | [10](http://desktop-0t1rtqn/millbin/msdigest.cgi?missed_cleavages=2&msparams_dir=msparams_mill/&hide_protein_sequence=2&database=NCBIgb_SA300sequence.fasta&seqdb_dir=D:\SeqDB\&enzyme=Trypsin&access_method=Accession+Number&accession_num=QCV68685.1&coverage_map=0+245+24+43+10+16) | 24.85 | 0 |
|  | 0.00E+00 | 5.93E+04 | 0.00E+00 |  |  |  |  |  |  |  |
| 165 | 4 | 0 | 0 | UDP-N-acetylglucosamine 1-carboxyvinyltransferase | 45110 | 5.54 | [AIA28594.1](http://www.ncbi.nlm.nih.gov/protein/AIA28594.1) | [9.9](http://desktop-0t1rtqn/millbin/msdigest.cgi?missed_cleavages=2&msparams_dir=msparams_mill/&hide_protein_sequence=2&database=NCBIgb_SA300sequence.fasta&seqdb_dir=D:\SeqDB\&enzyme=Trypsin&access_method=Accession+Number&accession_num=AIA28594.1&coverage_map=0+41+27+306+15+32) | 24.83 | 4 |
|  | 1.46E+06 | 0.00E+00 | 0.00E+00 |  |  |  |  |  |  |  |
| 166 | 1 | 0 | 1 | UDP-glucose 4-epimerase | 38842.6 | 5.45 | [AIA26742.1](http://www.ncbi.nlm.nih.gov/protein/AIA26742.1) | [20.7](http://desktop-0t1rtqn/millbin/msdigest.cgi?missed_cleavages=2&msparams_dir=msparams_mill/&hide_protein_sequence=2&database=NCBIgb_SA300sequence.fasta&seqdb_dir=D:\SeqDB\&enzyme=Trypsin&access_method=Accession+Number&accession_num=AIA26742.1&coverage_map=0+183+71+88) | 24.29 | 1 |
|  | 5.28E+05 | 0.00E+00 | 1.29E+04 |  |  |  |  |  |  |  |
| 167 | 3 | 0 | 0 | phosphonate ABC transporter ATP-binding protein | 28195.1 | 8.65 | [AIA27212.1](http://www.ncbi.nlm.nih.gov/protein/AIA27212.1) | [17.8](http://desktop-0t1rtqn/millbin/msdigest.cgi?missed_cleavages=2&msparams_dir=msparams_mill/&hide_protein_sequence=2&database=NCBIgb_SA300sequence.fasta&seqdb_dir=D:\SeqDB\&enzyme=Trypsin&access_method=Accession+Number&accession_num=AIA27212.1&coverage_map=0+18+23+37+21+148) | 24.17 | 3 |
|  | 2.73E+06 | 0.00E+00 | 0.00E+00 |  |  |  |  |  |  |  |
| 168 | 3 | 0 | 0 | UDP-diphospho-muramoylpentapeptide beta-N- acetylglucosaminyltransferase | 39696.1 | 8.63 | [AIA27907.1](http://www.ncbi.nlm.nih.gov/protein/AIA27907.1) | [13.7](http://desktop-0t1rtqn/millbin/msdigest.cgi?missed_cleavages=2&msparams_dir=msparams_mill/&hide_protein_sequence=2&database=NCBIgb_SA300sequence.fasta&seqdb_dir=D:\SeqDB\&enzyme=Trypsin&access_method=Accession+Number&accession_num=AIA27907.1&coverage_map=0+234+26+44+23+29) | 23.88 | 3 |
|  | 1.42E+06 | 0.00E+00 | 0.00E+00 |  |  |  |  |  |  |  |
| 169 | 3 | 0 | 0 | UDP-N-acetylmuramoyl-L-alanyl-D-glutamate synthetase | 50119.1 | 5.51 | [KMR18188.1](http://www.ncbi.nlm.nih.gov/protein/KMR18188.1) | [11.5](http://desktop-0t1rtqn/millbin/msdigest.cgi?missed_cleavages=2&msparams_dir=msparams_mill/&hide_protein_sequence=2&database=NCBIgb_SA300sequence.fasta&seqdb_dir=D:\SeqDB\&enzyme=Trypsin&access_method=Accession+Number&accession_num=KMR18188.1&coverage_map=0+50+27+20+25+327) | 23.75 | 3 |
|  | 2.90E+06 | 0.00E+00 | 0.00E+00 |  |  |  |  |  |  |  |
| 170 | 2 | 0 | 0 | 3-hydroxy-3-methylglutaryl-CoA reductase | 46321.2 | 5.83 | [AQR32325.1](http://www.ncbi.nlm.nih.gov/protein/AQR32325.1) | [12.4](http://desktop-0t1rtqn/millbin/msdigest.cgi?missed_cleavages=2&msparams_dir=msparams_mill/&hide_protein_sequence=2&database=NCBIgb_SA300sequence.fasta&seqdb_dir=D:\SeqDB\&enzyme=Trypsin&access_method=Accession+Number&accession_num=AQR32325.1&coverage_map=0+108+22+206+31+58) | 23.66 | 2 |
|  | 1.67E+06 | 0.00E+00 | 0.00E+00 |  |  |  |  |  |  |  |
| 171 | 0 | 7 | 0 | phenol soluble modulin | 4496.1 | 4.78 | [AQQ83720.1](http://www.ncbi.nlm.nih.gov/protein/AQQ83720.1) | [40.9](http://desktop-0t1rtqn/millbin/msdigest.cgi?missed_cleavages=2&msparams_dir=msparams_mill/&hide_protein_sequence=2&database=NCBIgb_SA300sequence.fasta&seqdb_dir=D:\SeqDB\&enzyme=Trypsin&access_method=Accession+Number&accession_num=AQQ83720.1&coverage_map=0+22+18+4) | 15.26 | 0 |
|  | 0.00E+00 | 4.59E+07 | 0.00E+00 |  |  |  |  |  |  |  |

**References:**

Aires, A., Marrinhas, E., Carvalho, R., Dias, C., and Saavedra M. J. (2016). Phytochemical composition and antibacterial activity of hydroalcoholic extracts of *Pterospartum tridentatum* and *Mentha pulegium* against *Staphylococcus aureus* isolates. *BioMed Res. Inter.* 2016, 1-11. doi: [10.1155/2016/5201879](https://doi.org/10.1155/2016/5201879)

Alvarez, R. A., Blaylock, M. W., and Baseman, J. B. (2003). Surface localized glyceraldehyde-3-phosphate dehydrogenase of *Mycoplasma genitalium* binds mucin. *Mol. Microbiol.* 48, 1417-1425. doi: [10.1046/j.1365-2958.2003.03518.x](https://doi.org/10.1046/j.1365-2958.2003.03518.x)

Corrigan, R. M., Miajlovic, H., and Foster, T. J. (2009). Surface proteins that promote adherence of *Staphylococcus aureus* to human desquamated nasal epithelial cells. *BMC microbiology*, *9*, 22. doi: [10.1186/1471-2180-9-22](https://doi.org/10.1186/1471-2180-9-22)

Dallo, S. F., Kannan, T. R., Blaylock, M. W., and Baseman, J. B. (2002). Elongation factor Tu and E1 beta subunit of pyruvate dehydrogenase complex act as fibronectin binding proteins in *Mycoplasma pneumoniae*. *Mol. Microbiol*. 46, 1041-1051. doi: [10.1046/j.1365-2958.2002.03207.x](https://doi.org/10.1046/j.1365-2958.2002.03207.x)

Fulde, M., Bernardo-García, N., Rohde, M., Nachtigall, N., Frank, R., Preissner, K. T., et al. (2013). Pneumococcal phosphoglycerate kinase interacts with plasminogen and its tissue activator. *Thromb. Haemost*. 111, 401-416. doi: [10.1160/TH13-05-0421](https://doi.org/10.1160/TH13-05-0421)

Harraghy, N., Kormanec, J., Wolz, C., Homerova, D., Goerke, C., Ohlsen, K., et al. (2005). sae is essential for expression of the staphylococcal adhesins Eap and Emp*. Microbiology* 151, 1789-1800. doi: [10.1099/mic.0.27902-0](https://doi.org/10.1099/mic.0.27902-0)

Katakura, Y., Sano, R., Hashimoto, T., Ninomiya, K., and Shioya, S. (2010). Lactic acid bacteria display on the cell surface cytosolic proteins that recognize yeast mannan. *Appl. Microbiol. Biotechnol*. 86, 319-326. doi: [10.1007/s00253-009-2295-y](https://doi.org/10.1007/s00253-009-2295-y)

Kesimer, M., Kiliç, N., Mehrotra, R., Thornton, D. J., and Sheehan, J. K. (2009). Identification of salivary mucin MUC7 binding proteins from Streptococcus gordonii. *BMC microbiol.* 9, 163. Doi: [10.1186/1471-2180-9-163](https://doi.org/10.1186/1471-2180-9-163)

Kinoshita, H., Uchida, H., Kawai, Y., Kawasaki, T., Wakahara, N., Matsuo, H., et. al. (2008). Cell surface *Lactobacillus plantarum* LA 318 glyceraldehyde-3-phosphate dehydrogenase (GAPDH) adheres to human colonic mucin. J. Appl. Microbiol. 104, 1667-1674. doi: [10.1111/j.1365-2672.2007.03679.x](https://doi.org/10.1111/j.1365-2672.2007.03679.x)

Mongodin, E., Bajolet, O., Cutrona, J., Bonnet, N., Dupuit, F., Puchelle, E., et al. (2002). Fibronectin-binding proteins of Staphylococcus aureus are involved in adherence to human airway epithelium. *Infect. Immune.* 70, 620-630.

Oshida, T., and Tomasz, A. (1992). Isolation and characterization of a Tn551-autolysis mutant of *Staphylococcus aureus*.  *J Bacterial.* 174, 4952-4959. doi: [10.1128/jb.174.15.4952-4959.1992](https://dx.doi.org/10.1128%2Fjb.174.15.4952-4959.1992)

Siciliano, R. A., Cacace, G., Mazzeo, M. F., Morelli, L., Elli, M., Rossi, M., et al. (2008). Proteomic investigation of the aggregation phenomenon in *Lactobacillus crispatus*. *Biochim. Biophys. Acta* 1784, 335-342. doi: [10.1016/j.bbapap.2007.11.007](https://doi.org/10.1016/j.bbapap.2007.11.007)

Smith, E. J., Corrigan, R. M., van der Sluis, T., Gründling, A., Speziale, P., Geoghegan, J., et al. (2012). The immune evasion protein Sbi of *Staphylococcus aureus* occurs both extracellularly and anchored to the cell envelope by binding lipoteichoic acid. *Mol. Microbiol****.* 83,** 789-804. doi: [10.1111/j.1365-2958.2011.07966.x](https://dx.doi.org/10.1111%2Fj.1365-2958.2011.07966.x)

Xolalpa, W., Vallecillo, A. J., Lara, M., Mendoza-Hernandez, G., Comini, M., Spallek, R., et al. (2007). Identifcation of novel bacterial plasminogen binding proteins in the human pathogen *Mycobacterium tuberculosis*. *Proteomics* 7, 3332-3341 doi: [10.1002/pmic.200600876](https://doi.org/10.1002/pmic.200600876)
